# Hardware acceleration of simulated annealing for constraint satisfaction problems

**DOI:** 10.1038/s41467-026-74893-1

**Published:** 2026-07-21

**Authors:** Andrew Pannone, Rishikesh T. Nair, Pranav Krishnan, Fabio Carletti, Harikrishnan Ravichandran, Priyatam Gundapuneni, Arav Dutta, Subir Ghosh, Chen Chen, Joan M. Redwing, Saptarshi Das

**Affiliations:** 1 Engineering Science and Mechanics, Penn State University, University Park, PA USA; 2 Elettronica, Informazione e Bioingegneria, Politecnico di Milano, Milan, Italy; 3 Mechanical Engineering, Penn State University, University Park, PA USA; 4 2D Crystal Consortium Materials Innovation Platform, Penn State University, University Park, PA USA; 5 Materials Science and Engineering, Penn State University, University Park, PA USA; 6 Materials Research Institute, Penn State University, University Park, PA USA; 7 Electrical Engineering, Penn State University, University Park, PA USA

**Keywords:** Electronic devices, Two-dimensional materials

## Abstract

Simulated annealing (SA), a metaheuristic algorithm inspired by physical annealing processes, attempts to solve combinatorial optimization problems by performing a stochastic search that iteratively explores the solution space. Probabilistic solution evaluation allows the algorithm to escape local minima and progressively converge toward a global optimum, even within complex solution landscapes. In this work, we present the hardware acceleration of SA for spatial optimization within constrained environments, specifically targeting drone placement that maximizes area coverage while avoiding no-fly zones. Stochasticity is introduced through a true random number generator (TRNG) based on two-dimensional (2D) materials. The system energy is evaluated using a 2D logic circuit and the acceptance of candidate solutions is governed by a programmable 2D circuit with tunable threshold behavior. For a representative drone placement task, our hardware-based annealing accelerator circuit modules consume 43 nJ of energy per iteration and achieve an 1800-fold search acceleration relative to brute-force exploration of the solution space. This work also underscores the potential of 2D-material-enabled stochastic and programmable hardware for real-time constrained optimization.

## Abstract (web summary)

Simulated annealing outperforms brute-force search in finding optimal solutions for computationally intensive tasks. Pannone et al. report a simulated annealing accelerator using 2D-material-based CMOS transistors to achieve high energy efficiency, search acceleration, and increased scalability.

## Introduction

The development and efficient implementation of algorithms that search for solutions to combinatorial optimization problems is a fundamental challenge in computational science, with applications spanning autonomous robotics^1,2^, intelligent infrastructure^3,4^, and large-scale logistical systems^5,6^. The solution space of a given optimization problem can be mapped into an energy landscape where the quality of each potential solution is represented by an associated system energy (H). Within this framework, an optimal solution will correspond to the system’s ground state energy configuration. Brute-force search tree methods that obtain the ground state solution of a constrained system exhibit exponential time complexity, often scaling as $O({b}^{n})$ for a branching factor *b* and decision depth *n*^7^. Given the NP-hard nature of many combinatorial optimization problems, metaheuristic algorithms such as simulated annealing (SA)^8^, quantum annealing (QA)^9^, genetic algorithms (GAs)^10^, particle swarm optimization (PSO)^11^ and ant colony optimization (ACO)^12^ offer a dramatic reduction in computational overhead by probabilistically exploring high-impact regions of the solution space, often converging to near-optimal solutions^13,14^.

Simulated annealing is analogous to thermal annealing where the temperature of a crystalline solid is elevated and then gradually reduced to enhance its atomic order. The simulated annealing algorithm emulates this process by introducing a thermally modulated acceptance criterion. During each iteration, proposed solutions that reduce system energy are always accepted while “worse” solutions that increase system energy will be accepted at a rate determined by the thermally modulated acceptance criterion. Traversal of the energy landscape is dictated by an annealing schedule that defines the series of temperatures and the number of iterations spent at each temperature^15^. At high temperatures, proposed solutions with greater energy than the current solution have an increased likelihood of acceptance, allowing the algorithm to explore varied regions of the solution space without becoming trapped in local minima. As the temperature descends, worse solutions will be accepted less frequently allowing the algorithm to converge toward a global minima.

Due to its proven utility in providing solutions to constrained optimization problems such as the traveling salesman problem^16^, the graph coloring problem^17^, the max-cut problem^18^, or the eight queens problem^19^, recent works have attempted to accelerate SA in a variety of hardware platforms, including graphics processing units (GPUs), field-programmable gate arrays (FPGAs), application-specific integrated circuits (ASICs) based on Si complementary metal-oxide-semiconductor (CMOS) technology, and ASICs based on emerging non-volatile memories (eNVMs). While these underlying computation systems differ substantially in area and power consumption, it is still critical to analyze the advantages and drawbacks of using each technology for SA. While GPUs and FPGAs can be reconfigured to support diverse problem formulations, ASICs lack flexibility. Nonetheless, they typically surpass GPUs and FPGAs in performance and energy efficiency for a given optimization task. GPUs have shown promise in accelerating combinatorial optimization problems^20^, yet their power efficiency remains poor compared with that of ASICs. FPGAs offer a high degree of reconfigurability^21–24^, but their achievable clock frequency and block random-access memory (BRAM) size impose practical limits on scaling to high-dimensional problems.

These limitations have contributed to recent interest in near-memory and in-memory computing with memristive devices^25^. One representative example employs resistive random-access memory (RRAM) and conductive-bridge random-access memory (CBRAM) to build an application-specific accelerator^23^. While this approach efficiently addresses a spin glass problem via in-memory matrix–vector multiplication and stochastic switching, the relatively large operating currents of eNVMs lead to non-negligible voltage drops across crossbar parasitic resistances^26^, reducing effective device conductance and thus degrading computational accuracy and increasing power consumption. Combining two different eNVM technologies on the same chip also adds fabrication complexity and cost. Alternatively, memtransistors that utilize 2D MoS_2_ or WSe_2_ transition-metal dichalcogenide materials offer exceptional scalability, enabling atomically thin channels without the loss of carrier mobility or electrostatic control. Moreover, their growth has been demonstrated to be both back-end-of-line (BEOL) compatible^27^ and vertically integrable^28^. Taken together, these characteristics point toward the potential capability of 2D-material-based ASICs to address much larger optimization problems than current alternatives.

To better evaluate the strengths and limitations of the various technologies, we compiled a comparison shown in Supplementary Data Table 1. Although the underlying optimization tasks differ, making a direct energy or power comparison impractical, we can meaningfully contrast the typical voltage ranges of the corresponding hardware. 2D materials-based circuits utilized in this work operate at higher voltages than several of the aforementioned technologies. However, further reductions in operating voltage are achievable by decreasing the dielectric thickness. Emerging NVM technologies, including memtransistors and RRAM/CBRAM, offer the ability to scale computation into the third dimension. Both 2D memtransistors (as demonstrated in our previous works)^25–31^ and RRAM/CBRAM exhibit compelling advantages such as BEOL compatibility, vertical integration, radiation tolerance, and reduced per-device power consumption. It is also important to note that the SA algorithm requires a source of random numbers that are used for the generation and probabilistic acceptance of proposed solutions. True random number generators based on 2D memtransistors and RRAMs^32,33^ are highly energy- and area- efficient in comparison to true random number generators based on Si CMOS that exploit frequency collapse in multimodal ring oscillators^34,35^, jitter in ring oscillators^36^, or metastability in latches^37^. Although RRAM and CBRAM are mature technologies, they lack intrinsic logic capabilities. Non-volatile and reconfigurable logic gates based on RRAMs have been demonstrated^38,39^, but each RRAM cell requires a dedicated transistor for programming. This dedicated transistor necessitates additional metal routing, resulting in increased pitch and parasitic effects. 2D memtransistors, on the other hand, are still far from industry level standards. However, they enable the integration of random number generation, memory, and logic, an essential requirement for this class of applications.

In this work, we expand upon past demonstrations of hardware-based annealing accelerators by heterogeneously integrating n-type MoS_2_ and p-type WSe_2_ FETs to create on-chip CMOS circuit modules that implement the SA algorithm. This hardware-based SA accelerator is used to demonstrate constrained spatial optimization by generating solutions for optimal drone placement that maximize system coverage within an environment that contains obstacles or no-fly zones. In a 10 × 10 discrete spatial domain with three obstacles and four drones, our annealing accelerator converged to a solution at a minimal energy expenditure of approximately 43 nJ per iteration while utilizing the SA algorithm to provide a search acceleration of 1800× relative to a brute-force search method. Moreover, simulations of the annealing accelerator illustrate that the degree of search acceleration scales favorably with both system size and agent count, demonstrating enhanced efficiency in larger and more complex configurations. Overall, these results underscore the potential of 2D-material-enabled stochastic and programmable hardware for real-time constrained optimization.

## Results and discussion

### 2D material-based annealing accelerator

**Fig. 1:**
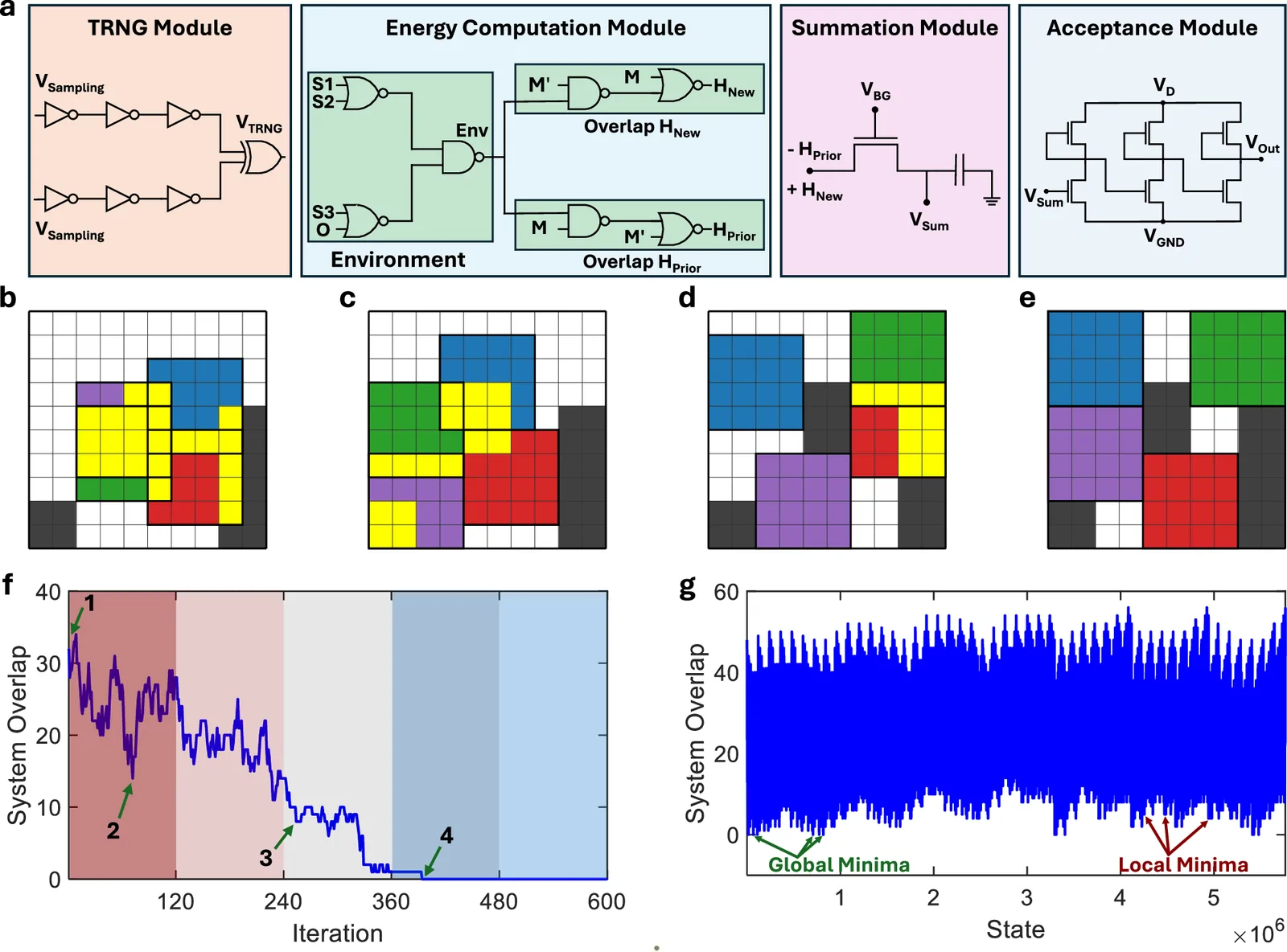
Simulated annealing overview. **a** Circuit schematic of all hardware modules utilized to accelerate simulated annealing (SA) for constrained optimization, including the true random number generator (TRNG) module, the energy computation module, the summation module, and the acceptance module. **b** Representation of a spatial optimization problem in which the objective is to deploy multiple agents, colored red, green, blue, and purple, within a grid to achieve maximum area coverage while minimizing system overlap and avoiding obstacles. Evolution of the SA optimization algorithm showing maps that correspond to (**c**, **d**) two local minima (iterations 72 and 254) and the (**e**) final optimized state reached at iteration 395. **f** System overlap as a function of annealing iteration. Successive reductions in system temperature after each 120 iterations as prescribed by an annealing schedule are depicted by the underlying color blocks. The marked points 1–4 correspond to the spatial configurations shown in (**b**–**e**). **g** Depiction of the energy landscape, revealing approximately 5.8 million possible system states and 24 distinct ground state energy (minimal overlap) configurations.

An overview of all hardware modules used to accelerate SA with 2D CMOS circuits is shown in Fig. 1a. This hardware-based annealing accelerator comprises a true random number generator (TRNG) circuit, an energy computation circuit, a summation circuit, and a thresholding circuit. For reference, a flowchart depicting the sequence of operations in a typical software-based SA algorithm is shown in Supplementary Fig. 1. Figure 1b illustrates a representation of a spatial optimization problem in which the objective is to deploy multiple agents within a grid to achieve maximum area coverage while adhering to constraints. This 10 × 10 system contains three static obstacle regions that must be avoided. The four 4 × 4 agents (red, green, blue, and purple) within the environment are permitted to move in any of four directions: up, down, left, or right. Overlap, highlighted in yellow, reflects redundant coverage either among agents or between agents and obstacles. The spatial occupancy of each agent is modeled as an independent map where cells corresponding to their occupied positions have a value of one and all other cells have a value of zero. Supplementary Fig. 2a-e shows the initial occupancy maps of all agents and obstacles separately.

The SA algorithm begins with a randomly assigned initial solution and a predefined annealing schedule. During each iteration, a proposed solution is created by sampling the TRNG module, which produces a stochastic bitstream that encodes random numbers corresponding to the random selection of an agent and its proposed movement direction. The energy computation module then uses Boolean logic to evaluate the overlap between the moving agent and other agents or obstacles. The overlap unique to the prior solution, ${H}_{{{{\rm{Prior}}}}}$, is subtracted from the overlap unique to the new solution, ${H}_{{{{\rm{New}}}}}$, using the summation module, which yields the energy difference, ΔH. Finally, the acceptance function module makes the decision whether to accept or reject the proposed solution based on the current system temperature and ΔH. Due to the programmable and nonvolatile nature of the constituent memtransistors, this module can be programmed to reflect the current temperature prescribed by the annealing schedule. This acceptance mechanism ensures a balance between exploration and exploitation, where the system is allowed to explore more freely at high temperatures before it converges toward one of the global minima as the temperature is reduced.

A simulation of the annealing accelerator was created to model the operations performed by each circuit module given an arbitrary system configuration and annealing schedule. More information on the simulation of the annealing accelerator can be found in the “Methods” section. This simulation was used to illustrate the progression of solution quality throughout the annealing process for the 10 × 10 system with 4 agents of size 4 × 4. The annealing schedule comprised 120 iterations per temperature at five descending temperatures for a total of 600 iterations. Figure 1c, d shows the occupancy map of the agents at iterations 72 and 254, respectively, while Fig. 1e shows the final optimized state of the system that was reached at iteration 395. Figure 1f depicts the total system overlap as a function of iteration with progressive reductions in temperature illustrated by the color of the underlying pane. Arrows 1, 2, 3, and 4 denote the system overlap corresponding to iterations 1, 72, 254, and 395, respectively. Clearly, iterations 72 and 254 correspond to local minima where the agents are required to move across high overlap solutions to eventually reach an optimal zero overlap solution. Bounding all agents strictly within the environment for simplicity, brute-force search would require 5,764,801 trials to guarantee an exhaustive exploration of the solution space. The overlap of all possible system states is shown in Fig. 1g. In comparison, the SA algorithm finds a solution in under 400 iterations. This annealing accelerator simulation serves as a testbed for evaluating the performance and convergence behavior of the hardware-based simulated annealing accelerator.

### Accelerator modules created with two-dimensional transistors

**Fig. 2:**
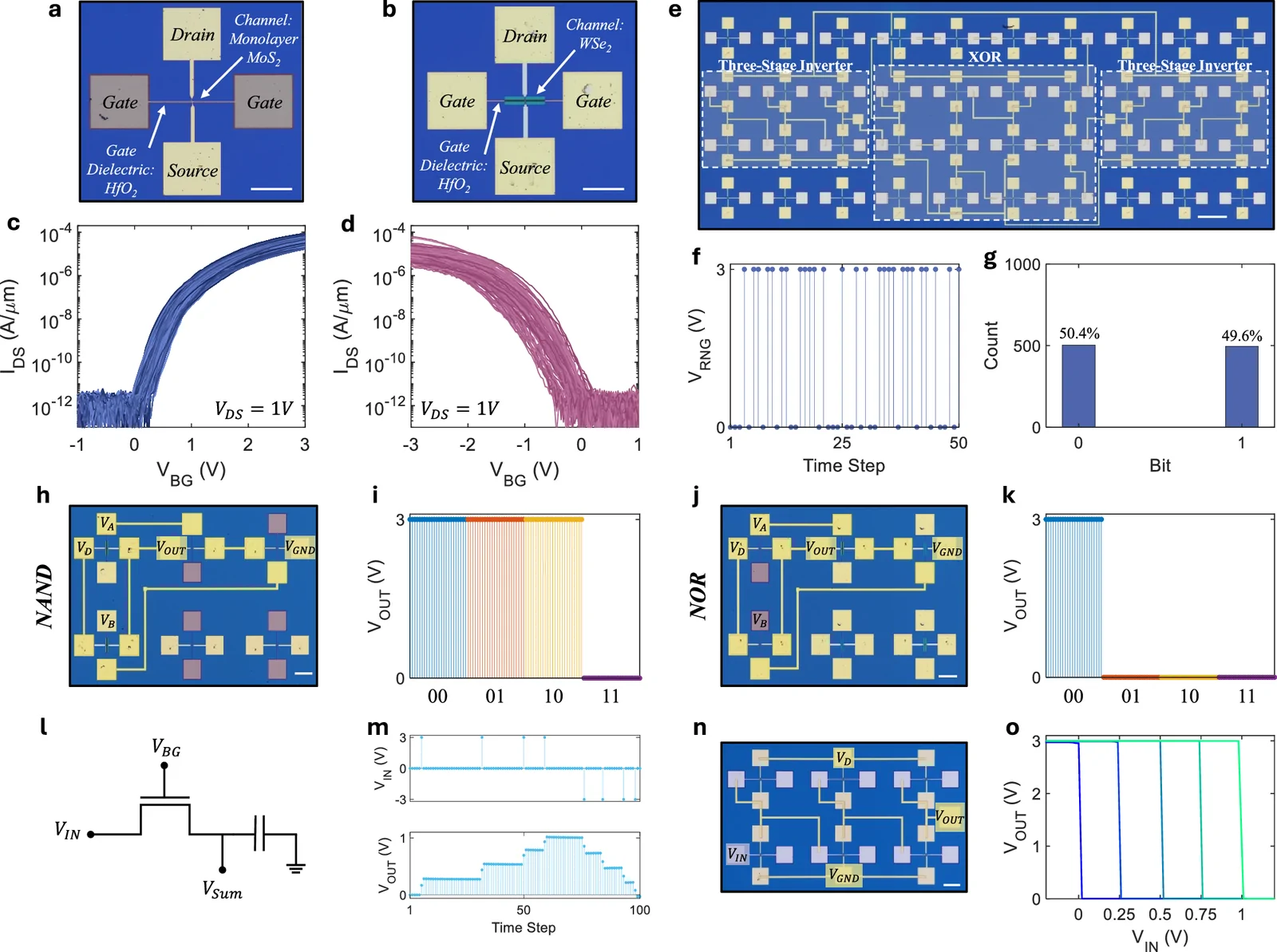
Logic gates enabled by 2D CMOS FETs. Optical image of FETs that utilize (**a**) MoS_2_ and (**b**) WSe_2_ as the semiconducting channel, respectively (scale bar: 30 μm). Transfer characteristics of (**c**) 100 MoS₂ and (**d**) 100 WSe₂ FETs, respectively. Optical image of (**e**) true random number generator (TRNG) module (scale bar: 100 μm). **f** Representative sample of a generated random bitstream and (**g**) histogram of 1000 output values showing an approximately equal distribution of 0’s and 1’s. **h** Optical image of NAND logic gate (scale bar: 40 μm) and (**i**) corresponding experimental output. **j** Optical image of NOR logic gate (scale bar: 40 μm) and (**k**) corresponding experimental output. **l** Circuit schematic of the summation module composed of a MoS₂ transistor and a capacitor and (**m**) analog output response given an input voltage stream *V*_IN_. **n** Optical image of MoS₂-based inverter circuit (scale bar: 40 μm) and (**o**) corresponding experimental outputs at several switching thresholds.

The prior demonstration of an annealing accelerator created with FETs based on two-dimensional materials utilized exfoliated flakes and externally connected logic circuits. Here, we integrate large area metal-organic chemical vapor deposition (MOCVD) grown MoS_2_ and WSe_2_ films as the semiconducting channel material for n-type and p-type FETs, respectively. Further details regarding the MOCVD film synthesis and transistor fabrication process can be found in the “Methods” section and previous reports^29,40^. A Raman spectrum of the MoS_2_ film used in this study is shown in Supplementary Fig. 3a. The ${E}_{2{{{\rm{g}}}}}^{1}$ and ${A}_{1{{{\rm{g}}}}}$ Raman active modes found in MoS_2_ are present at 384.2 cm^−1^ and 405.2 cm^−1^, respectively. A Raman spectrum of the WSe_2_ film used in this study is shown in Supplementary Fig. 3b. The ${E}_{2{\rm g}}^{1}\ {{\mbox{and}}} \ {A}_{1{\rm g}}$ Raman active modes create a peak located at 249.6 cm^−1^. The ${B}_{2{{{\rm{g}}}}}$ Raman active mode at ~310 cm^−1^ indicates that the grown WSe_2_ film is multilayer^41–43^. Optical images of representative MoS_2_ and WSe_2_ transistors are presented in Fig. 2a, b, respectively. Cross-sectional schematics of these transistors are depicted in Supplementary Fig. 4a-b. Figure 2c, d shows the transfer characteristics, i.e., the drain current (${I}_{{{{\rm{DS}}}}}$) as a function of the applied back-gate voltage (${V}_{{{{\rm{BG}}}}}$) at a constant drain voltage (${V}_{{{{\rm{DS}}}}}$), of 100 MoS_2_ and 100 WSe_2_ transistors, respectively. Supplementary Fig. 5 shows distributions of common FET performance metrics, including field-effect mobility (${\mu }_{{{{\rm{FE}}}}}$), subthreshold slope (${SS}$), threshold voltage (${V}_{{{{\rm{TH}}}}}$) and on-state current (${I}_{{{{\rm{ON}}}}}$) for the MoS_2_ and WSe_2_ transistors. These transistors act as the fundamental logic elements in the energy computation module of the hardware-based annealing accelerator.

The TRNG module utilizes two three-stage cascaded inverters and one XOR gate to generate true random numbers. An optical image of this circuit is depicted in Fig. 2e and a corresponding transistor-level circuit diagram is shown in Supplementary Fig. 6. The dual-sweep transfer characteristics of a representative three-stage cascaded inverter are shown in Supplementary Fig. 7. Random temporal fluctuations can be observed by sampling the three-stage inverter at an appropriate input voltage, ${V}_{{{{\rm{Sampling}}}}}$, within the hysteresis window between the forward and reverse voltage sweeps. The output of each three-stage cascaded inverter is given as in input to the XOR gate. This circuit leverages inherent material defects to generate stochastic bitstreams that randomly dictate the agent selection and movement direction during each annealing iteration. We sampled 10^6^ random bits and evaluated their randomness using the National Institute of Standards and Technology (NIST) SP 800-22 statistical test suite. Each test in this suite generates a *p*-value, which is evaluated at a 99% confidence level. Bitstreams with a *p*-value greater than 0.01 can be considered random. Supplementary Table 2 shows that the generated bit stream passes all 15 NIST SP 800-22 tests without any post-processing. We characterize the entropy source of this work using the NIST SP 800-90B statistical test suite. This test suite determines if an entropy source is independent and identically distributed (IID) or non-IID before estimating the minimum entropy. The results of the test suite after non-IID classification are shown in Supplementary Table 3. Here, the compression test calculates a minimum entropy of 0.8615. Further discussion on the true random nature of generated bits and the working principles of the TRNG module is present in our prior work^32^. The output of the TRNG module for 50 samples is represented as a time-domain stem plot, as seen in Fig. 2f, and a histogram depicting the frequency of 0 and 1 in a representative TRNG bitstream is shown in Fig. 2g.

After determination of the moving agent and its movement direction, the proposed agent location is updated and given to the energy computation module as a binary bitstream. This module is separated into three submodules; one environment computation submodule that determines the surrounding environment from the perspective of the moving drone and two overlap computation submodules that calculate the overlap unique to the prior solution and the overlap unique to the proposed solution. A transistor-level circuit diagram depicting the entire energy computation module is shown in Supplementary Fig. 8. Optical images of the environment computation submodule and two overlap computation submodules are shown in Supplementary Fig. 9a-c, respectively, with relevant input and output nodes labeled. The environment computation submodule comprises one NAND gate and two NOR gates, while the overlap computation modules comprise one NAND gate and one NOR gate. A representative NAND gate and its corresponding logic output are shown in Fig. 2h, i, respectively. Similarly, an optical image of a representative NOR gate and its corresponding logic output are shown in Fig. 2j, k, respectively. The Boolean logic functions implemented by the environment and overlap computation submodules will be discussed in the following section. After energy computation, the summation module computes ΔH using the circuit shown in Fig. 2l. This resistor–capacitor (RC) circuit utilizes an MoS_2_ transistor as a variable resistor to aid in the summation of output voltage values from the energy computation module. When logic high inputs are given to this circuit, the simultaneous application of a positive back-gate voltage, ${V}_{{{{\rm{BG}}}}}$, causes the MoS_2_ transistor to operate in a high conductance state, allowing the capacitor to charge. Negative inputs accompanied by a positive ${V}_{{{{\rm{BG}}}}}$ cause the capacitor to discharge. Negative ${V}_{{{{\rm{BG}}}}}$ is supplied to the MoS_2_ transistor alongside zero-valued inputs, causing the transistor to operate in its off state, increasing the RC constant and preventing capacitor discharge. The summation of several positive and negative input signals is shown in Fig. 2m.

Finally, a three-stage cascaded NMOS inverter comprising six memtransistors is used to implement the acceptance procedure found in simulated annealing algorithms. A cross-sectional schematic of a representative memtransistor is depicted in Supplementary Fig. 4c. An optical image of a three-stage NMOS inverter is shown in Fig. 2n. The switching threshold of the inverter, ${V}_{{{{\rm{SW}}}}}$, is configured through the programming of individual memtransistors using a voltage pulse. Supplementary Fig. 10a, b illustrates that positive and negative voltage pulses of varying magnitudes can be applied to change the analog conductance state of the MoS_2_ memtransistors, while Supplementary Fig. 11 shows the retention of programmed states over time. The endurance of a representative MoS_2_ memtransistor was also evaluated. We define a single endurance cycle as the sequential pulsing of –10 V and 10 V to the back-gate of the memtransistor for a time duration of 1 μs. These negative and positive voltage pulses program the memtransistor into a low-resistance state (LRS) and a high-resistance state (HRS), respectively. This procedure was repeated for 10^5^ cycles. The LRS and HRS were recorded at a back-gate voltage of −2 V and a drain voltage of 1 V after 10, 100, 1000, 10,000, and 100,000 programming cycles. Supplementary Fig. 12a shows the evolution of the LRS and HRS as a function of the cycle count. Both states remain well separated across the full endurance experiment, demonstrating stable programmability. Supplementary Fig. 12b presents retention characteristics at four programmed analog memory states following the endurance experiment. The retention of each analog memory state was monitored for 200 s. These results confirm that the stability and retention of programmed memory states are not significantly compromised by 10^5^ programming cycles. Figure 2o shows transfer characteristics of the NMOS inverter at different switching thresholds (${V}_{{{{\rm{SW}}}}}=$ 0 V, 0.25 V, 0.5 V, 0.75 V, and 1 V). Here, ${V}_{{{{\rm{SW}}}}}$ corresponds to the temperature of the annealing system and the output will either yield a logic 1 if the proposed solution is accepted or a logic 0 if the proposed solution is rejected. In this manner, the NMOS inverter circuit is able to accept worse solutions conditionally based on their relative energy increase. At any temperature, negative ΔH inputs that reduce system energy will always be accepted. At higher temperatures, positive ΔH inputs representing worse solutions will be accepted if ${V}_{{{{\rm{SW}}}}} > \Delta H$. At the minimum temperature represented by ${V}_{{{{\rm{SW}}}}}=\, 0 \, {{\rm{V}}}$, no proposed solutions with positive ΔH will be accepted. Together, these modules form a hardware implementation of the simulated annealing algorithm. The hardware realization of randomness, energy evaluation, summation, and thermally modulated acceptance enables a framework for solving spatial constraint optimization problems.

### Drone placement in a constrained environment

**Fig. 3:**
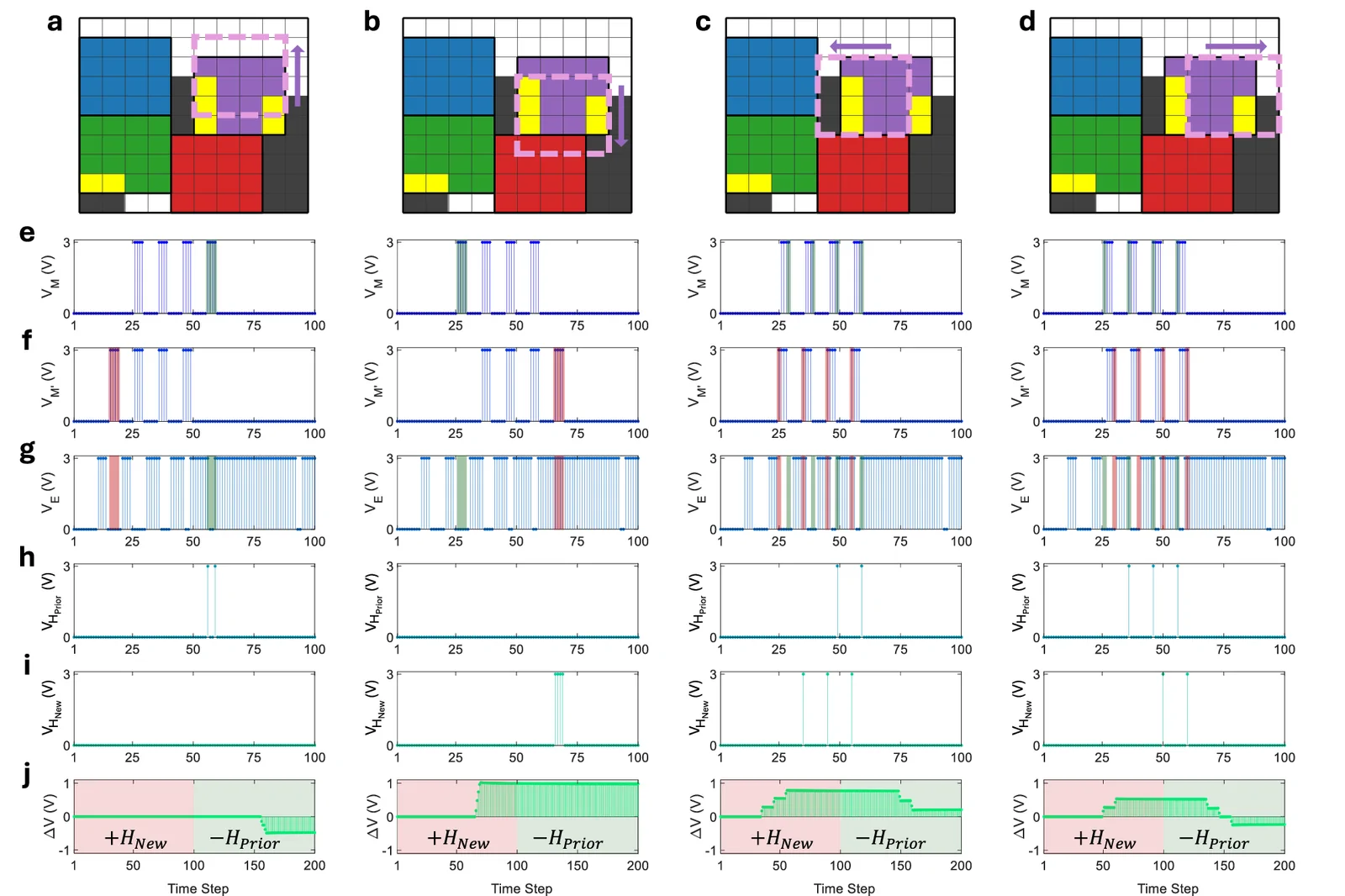
Implementation of SA using 2D hardware for constraint satisfaction. Illustration of four possible movement directions for the purple drone in a given four-drone, three-obstacle system: (**a**) up, (**b**) down, (**c**) left, and (**d**) right. Proposed movement positions are illustrated by the dashed pink lines. Bitstreams corresponding to the (**e**) initial occupancy of the moving drone, ${{{\rm{M}}}}$, and (**f**) the proposed occupancy of the moving drone, ${{{\rm{M}}}^{\prime}}$. **g** Bitstream corresponding to ${{{\rm{E}}}}$ for each movement direction. Stem plots of (**h**) ${{{{\rm{H}}}}}_{{{{\rm{Prior}}}}}$ and (**i**) ${{{{\rm{H}}}}}_{{{{\rm{New}}}}}$ for each movement direction. **j** The output of the summation module that is subsequently processed by the acceptance module.

Next, we utilize the hardware-based annealing accelerator in a spatial optimization problem where four drones of size 4 × 4 require placement with minimal overlap in a 10 × 10 spatial domain containing three obstacles. Figure 3a-d shows the randomly initialized state of the system and the proposed state of the system considering upward, downward, leftward, or rightward movement of the purple drone, respectively. The solid purple box is representative of the initial drone position and the dashed pink line indicates the proposed drone position chosen based on the output of the TRNG module. The first two bits of the TRNG module bitstream determine the identity of the moving drone (00, 01, 10, or 11 for drone 1, drone 2, drone 3, or drone 4) while the next two bits determine the agent movement direction (00, 01, 10, or 11 for upward, downward, leftward, or rightward movement). Next, the position of each drone is encoded into a binary occupancy map and flattened into a bitstream. The row-wise vectorization procedure that was used to transform binary occupancy maps into flattened bitstreams is illustrated in Supplementary Fig. 13a-c. The energy computation module evaluates the change in system energy by performing logic operations on the encoded bitstreams. Input bitstreams corresponding to the initial occupancy of the moving drone, M, and the proposed occupancy of the moving drone, $M^{\prime}$, are shown in Fig. 3e, f, respectively. The occupancies of all other drones and obstacles are compressed together by the environment computation submodule as seen in Eq. 1 below:

1 $$E={S}_{1}+{S}_{2}+{S}_{3}+O$$

Here, E is the environment seen by the moving drone, ${S}_{1}$ is the occupancy of the first stationary drone, ${S}_{2}$ is the occupancy of the second stationary drone, ${S}_{3}$ is the occupancy of the third stationary drone, and O is the occupancy of the system obstacles. Stem plots depicting the experimental output of the environment computation submodule are shown for each movement direction in Fig. 3g. The variable E is not dependent on movement direction, so each stem plot is identical. Supplementary Fig. 14a-d shows the occupancy maps of all stationary drones and obstacles individually, while Supplementary Fig. 14e shows the combined environment occupancy map.

Subsequently, the overlap computation submodules are used to quantify the system overlap unique to the prior solution, ${H}_{{{{\rm{Prior}}}}}$, and the system overlap unique to the proposed solution, ${H}_{{{{\rm{New}}}}}$, as seen in Eq. 2 and Eq. 3 below:

2 $${H}_{{{{\rm{Prior}}}}}=E*M*\bar{M^{\prime} }$$

3 $${H}_{{{{\rm{New}}}}}=E*\bar{M}*M^{\prime}$$

Within these equations, the terms $M*\bar{M^{\prime} }$ and $\bar{M}*M^{\prime}$ isolate the occupancy that is unique to the drone in the prior solution and the new solution, respectively, while multiplication with E identifies overlapping regions. Stem plots of ${H}_{{{{\rm{Prior}}}}}$ and ${H}_{{{{\rm{New}}}}}$ depicting the experimental output of the overlap computation submodules are shown for each movement direction in Fig. 3h, i, respectively. Next, ${H}_{{{{\rm{Prior}}}}}$ and ${H}_{{{{\rm{New}}}}}$ are given as inputs to the summation module that computes Eq. 4 listed below:

4 $$\Delta H={H}_{{{{\rm{New}}}}}-{H}_{{{{\rm{Prior}}}}}$$

This module sums the entire bitstream, as seen in Fig. 3j, compressing all information into a single-valued ΔH that is fed into the acceptance function module. As seen, upward motion yields a $\Delta H\approx -0.5\,{\mbox{ V}}$ because half of the drone area unique to the prior solution was overlapping with the environment while the drone area unique to the proposed solution completely lacks overlap. This proposed solution is better than the prior solution and will always be accepted. In contrast, rightward movement yields a nominal $\Delta H\approx -0.25\,{\mbox{ V}}$ since some unique overlap exists in both the prior and proposed solutions. Nonetheless, the proposed solution reduces overlap and will always be accepted. Leftward and downward movement yield ΔH values of ~0.25 V and ~1 V, respectively. Depending on the temperature prescribed by the annealing schedule, leftward movement may be accepted, allowing the algorithm to explore the solution space and potentially escape a local minimum. Nonetheless, a ΔH value of ~1 V indicates that the proposed solution is significantly worse than the prior solution. As a result, the proposed downward movement would be rejected at most temperatures.

**Fig. 4:**
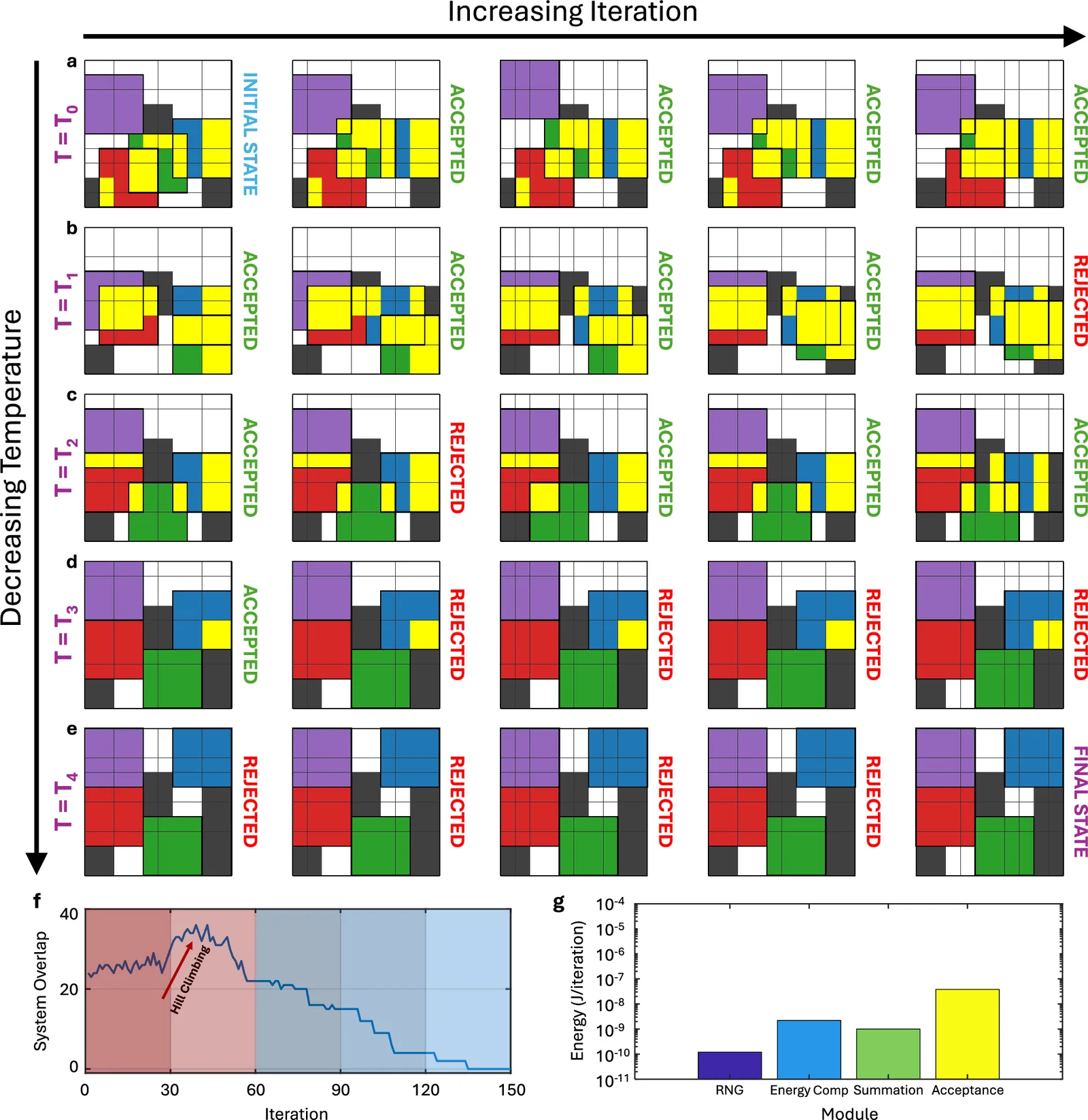
Evolution of the drone system. **a–e** Visualization of drone placement optimization using simulated annealing across five different annealing temperatures, demonstrating progressive improvement in coverage and overlap minimization. **f** Overlap as a function of iteration corresponding to the simulated annealing process shown in (**a**–**e**), highlighting convergence behavior over iterations. **g** Estimated module-wise energy consumption per individual drone movement.

After establishing the validity of the hardware-based annealing accelerator over one SA iteration, the same drone system was evaluated in hardware with an annealing schedule comprising 30 iterations per temperature across 5 descending temperatures for a total of 150 iterations. Figure 4a-e shows the state of the hardware-generated solution at five consecutive iterations for each of the five temperatures. Supplementary Video 1 shows the evolution of the system over all 150 iterations. Clearly, the annealing accelerator explores the solution space at higher temperatures before converging to an optimal solution by the 14^th^ iteration of the final temperature. The system overlap as a function of iteration is shown in Fig. 4f. The trend here confirms that the system overlap fluctuates at first as the solution space is explored, but drifts towards a global minimum at lower temperatures as the number of iterations increases.

The annealing accelerator simulation was utilized to quantify the error introduced by each hardware module. Ideal values for the output of the energy computation module, the summation module, and the acceptance module were determined through simulation utilizing an identical initialization of the four-drone, three-obstacle 10 × 10 system and the experimentally realized string of TRNG bits. The experimental and ideal values of ${H}_{{{{\rm{Prior}}}}}$ and ${H}_{{{{\rm{New}}}}}$ are shown for all 150 simulated annealing iterations in Supplementary Fig. 15a, b, respectively, alongside the module errors. Note that 15,000 total time steps are shown, corresponding to 100 time steps per iteration. Comparison of experimental and ideal values of ${H}_{{{{\rm{Prior}}}}}$ and ${H}_{{{{\rm{New}}}}}$ reveals that the 2D CMOS-based logic circuits exhibit negligible error. Next, the experimental and ideal outputs of the summation module are shown alongside the module error for all 150 simulated annealing iterations in Supplementary Fig. 15c. During each iteration, this module sums 200 values corresponding to -${H}_{{{{\rm{Prior}}}}}$ and ${H}_{{{{\rm{New}}}}}$. The mean absolute error (MAE) of this module is approximately 189.9 mV. Here, it is important to note that despite an MAE of 189.9 mV, the hardware-based annealing accelerator does not deviate from the ideal movement decisions determined by the annealing accelerator simulation. Subsequently, the experimental and ideal outputs of the acceptance module are shown alongside the module error for all 150 simulated annealing iterations in Supplementary Fig. 15d. The acceptance module error is negligible and will not impact subsequent logic operations.

To perform this optimization task, the hardware modules in the annealing accelerator consume approximately 43.36 nJ of energy per iteration, depending on the nature of the prior and proposed solutions. Figure 4g shows a bar plot depicting the energy consumption of each accelerator module. Additional details regarding the calculation of energy consumption are present in the “Methods” section. This hardware-based annealing accelerator consists of several modules with the following transistor counts: the TRNG module utilizes 22 transistors, the environment submodule utilizes﻿ 12 transistors, the overlap submodules each utilize﻿ 8 transistors, the summation module utilizes 1 transistor, and the thresholding module utilizes 6 transistors. The accelerator can be expanded to accommodate larger systems. The environment module is the only circuit that scales with the number of drones and obstacles. Specifically, for every 3n+1 drones and one obstacle input, the system requires an additional 3n two-input logic gates comprising 2n NOR and n NAND gates for environment computation. Here, n is an integer that describes the scaling factor. Functionally, this module performs an AND operation on the encoded representations of all stationary elements within a given iteration. Although the number of obstacles may increase, the entire obstacle configuration can be efficiently encoded as a single bitstream. It is also important to note that the hardware-based annealing accelerator demonstrated here could potentially be generalized to other optimization problems set in a discrete spatial domain with arbitrary constraints. For example, the eight queens problem could be solved by initializing agents of the shape described by the allowable movement regions of a queen chess piece. By minimizing overlap between agents, the system would potentially converge to a permissible solution. Nonetheless, the exact circuit implementation of our work cannot directly be generalized to optimization problems such as graph coloring, max-cut, sudoku, or the traveling salesman problem. However, only the energy computation and summation modules of the hardware-based annealing accelerator would require modification to create a hardware-based annealing accelerator capable of solving these problems. The remaining TRNG and acceptance modules would not require modification as they perform general-purpose annealing operations.

Next, circuit timing diagrams will be shown in response to a representative rightward movement of the green drone from the system depicted in Supplementary Fig. 16. Supplementary Fig. 17a shows the timing diagram of the TRNG module, where a random bit is sampled every 5 ms. Four total bits are required per simulated annealing iteration to select the drone and its proposed movement direction, resulting in a total of 20 ms per iteration for this module. In the example shown, the generated sequence is (0, 1, 0, 1). For illustrative purposes, we will assume that the first pair (0, 1) selects the green drone, while the second pair (0, 1) corresponds to a rightward proposed motion. Next, Supplementary Fig. 17b presents the timing response of the energy computation module, which comprises three NAND and four NOR operations. In this implementation, the first two NOR operations are evaluated in parallel to produce intermediate node voltages A and B within ~2 s operating at 20 ms per input over 100 inputs corresponding to the entire occupancy map. Intermediate voltages A and B are given to a final NAND gate, which computes E with identical timing properties. Next, the overlap unique to the prior solution, ${H}_{{{{\rm{Prior}}}}}$, and the overlap unique to the new solution, ${H}_{{{{\rm{New}}}}}$, are computed with combinational logic comprising cascaded NAND and NOR operations. Here, two parallel two-input NAND gates generate C and D in 2 s. Finally, two parallel NOR gates produce ${H}_{{{{\rm{Prior}}}}}$ and ${H}_{{{{\rm{New}}}}}$. Thus, the total time for the energy computation module is approximately 2 s per iteration. Next, Supplementary Fig. 17c shows the timing diagram of the summation module, which computes the net energy change by adding -${H}_{{{{\rm{Prior}}}}}$ and ${H}_{{{{\rm{New}}}}}$, yielding a positive ${V}_{{{{\rm{Sum}}}}}$ of ~1 V in the case shown due to the fact that ${H}_{{{{\rm{New}}}}}$ contains four high values and ${H}_{{{{\rm{Prior}}}}}$ does not contain any high values. This circuit is sampled every 30 ms and processes 200 inputs per iteration, resulting in a total duration of 6 s for the summation step. Supplementary Fig. 17d depicts the timing diagram of the acceptance module, which is implemented using an inverter operating at a 30 ms sampling rate. Assuming operation at a temperature corresponding to an inverter switching threshold of 750 mV, the input ${V}_{{\mathrm{Sum}}}=1\,{\mathrm{V}}$ results in a logical output $V_{{\mathrm{OUT}}}=0\,{\mathrm{V}}$ indicating that the proposed move was rejected.

The time required for each module to complete its respective operations during each iteration is summarized in Supplementary Fig. 18. At present, the overall computation speed is limited by the summation module, resulting in an effective computation rate of approximately 8.05 seconds per iteration. Although this speed is relatively low, it can be significantly improved by increasing the measurement speed. Our prior works have demonstrated logic operations at frequencies up to 25 kHz^44^, which would reduce the energy computation time per iteration to 4 ms. Furthermore, the summation time can be lowered by sampling at 25 kHz with an appropriate capacitor choice in conjunction with the MoS_2_ transistor, which would result in a summation time of approximately 8 ms per iteration. With these projected improvements, the per-iteration timing would be approximately 20 ms for the TRNG module, 4 ms for the energy computation module, 8 ms for the summation module, and 5 ms for the acceptance module, yielding a total of 37 ms per iteration (∼27 Hz). For the system demonstrated experimentally requiring 150 iterations, this corresponds to a convergence time on the order of 5.6 s. A typical microcontroller operates at MHz frequencies and therefore would have a negligible impact on the overall system speed. Additionally, the 5 ms/bit TRNG module sampling time does not reflect the fundamental sampling limit of the entropy source. In comparison, TRNGs based on RRAM technology have been reported with higher raw bit generation bandwidths in the range of kbps–Mbps^45^ depending on the scheme and readout. Higher bitrates for TRNGs based on MoS_2_ memtransistors are achievable at the circuit level by operating multiple TRNG modules concurrently or at the device level with defect/interface engineering that aims to accelerate the underlying stochastic dynamics at the semiconductor–dielectric interface^46–51^.

In our implementation of simulated annealing, all core operations, including random number generation, energy computation, summation, and solution evaluation, are performed by the TRNG, energy computation, summation, and acceptance modules, respectively. For this hardware demonstration, each module was measured separately using a probe station. Additional details regarding the measurement setup are present in the “Methods” section. While this modular hardware implementation serves as a proof-of-concept, further development is required to create a fully integrated hardware-based annealing accelerator. The only external peripheral required is a compact memory-and-control unit (e.g., a microcontroller with local memory), which is inserted between the TRNG module and the energy computation module and is in feedback with the output of energy computation and acceptance modules as illustrated in Supplementary Fig. 19. A representation of the memory configuration is shown in Supplementary Fig. 20a where 10 × 10 occupancy maps are stored for all four drones (D1–D4) at addresses 00, 01, 10, and 11. One additional map is stored at address X that encodes all stationary obstacles in the environment.

At each annealing iteration, four bits are generated by the TRNG module and written into the microcontroller memory. The first two bits select the moving drone by addressing its stored map. Supplementary Fig. 20b shows a representative example of this operation given random bits (0, 1) where the map at address 01 corresponding to D2 is designated as the active map, M, and a copy is written into the next available memory location, Y. The remaining two bits encode the proposed motion of the selected drone (00, 01, 10, or 11 for upward, downward, leftward, or rightward movement). This motion is applied to M to generate M′, which is stored in memory location Z, as illustrated in Supplementary Fig. 20c. At this stage, six maps are streamed to the energy computation module: M and M′ for the original and proposed occupancy maps of the moving drone, D1, D3, and D4 for the three stationary drones (S1, S2, and S3), and O for the static obstacles. Then, two additional memory addresses are created to store the outputs of the energy computation module, ${H}_{{{{\rm{Prior}}}}}$ and ${H}_{{{{\rm{New}}}}}$. Next, -${H}_{{{{\rm{Prior}}}}}$ and ${H}_{{{{\rm{New}}}}}$ vectors are sequentially given to the summation module. The output of the summation module is sent to the acceptance module. The resulting 1-bit acceptance signal is fed back to the microcontroller. Supplementary Fig. 20d shows the resulting memory update that occurs if the acceptance signal is 1, indicating that the proposed drone movement was accepted. In this case, the occupancy map at address 01 corresponding to D2 is updated with the approved movement, M′. Supplementary Fig. 20e illustrates that if the acceptance module output signal is 0, the proposal is rejected and the occupancy map at address 01 corresponding to D2 remains unchanged. In this manner, the full simulated annealing loop is realized with a single, well-defined memory/control peripheral tightly integrated around the existing modules. The proposed design requires only memory and simple control logic. These functionalities can be directly implemented on a custom printed circuit board with a basic microcontroller that minimizes additional energy overhead.

### Simulation of annealing accelerator

A simulation was developed to investigate the efficacy of our annealing accelerator in varied test cases. First, the performance of the simulation was analyzed on the same 10 × 10 four-drone system with three obstacles that was evaluated in hardware. The initial positions of all drones were kept constant across hardware and software initializations, but simulation utilized pseudo-random bits from the Python “random” module rather than bits generated by our TRNG module. We assess the convergence behavior of this system in software by evaluating 100 SA initializations. The system overlap as a function of iteration is shown for all 100 initializations in Supplementary Fig. 21a. We found that 66 out of the 100 initializations converged to an ideal solution, which corresponds to a failure rate of 34%. Supplementary Fig. 21b shows a cumulative distribution plot that illustrates the number of iterations required to reach an optimal solution for the 100 random initializations. For the subset of simulation initializations that converged, the mean and median number of iterations required to reach an optimal solution were 112.98 and 113.5, respectively, with a standard deviation of 18.54 iterations. The hardware-based annealing accelerator converged at iteration 134, which is slightly slower than the average software initialization by approximately one standard deviation. This discrepancy in convergence speed can be attributed to the inherent variability of the simulated annealing algorithm.

**Fig. 5:**
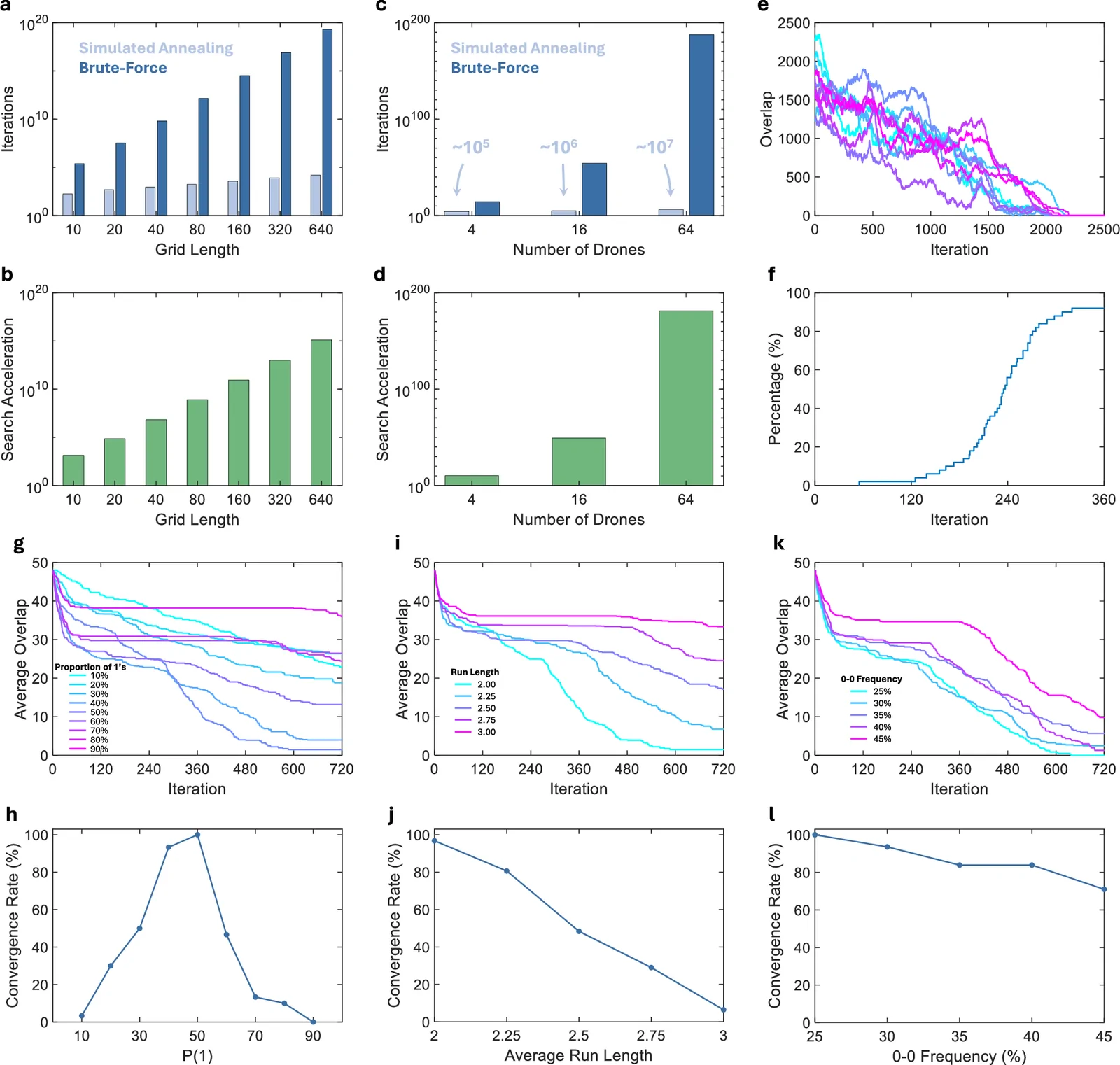
Simulation of annealing accelerator. **a** The number of iterations required to solve the base four-drone, three-obstacle constrained spatial optimization problem and (**b**) corresponding search acceleration at scaling factors of 1, 2, 4, 8, 16, 32, and 64 representing grid sizes of 10 × 10, 20 × 20, 40 × 40, 80 × 80, 160 × 160, 320 × 320, and 640 × 640. **c** The number of iterations required to solve 4, 16, and 64 drone optimization problems and (**d**) corresponding search acceleration. **e** System overlap as a function of iteration shown for 10 different random initializations of the four-drone, three-obstacle problem with an 80 × 80 grid size. **f** Cumulative distribution plot of the number of iterations required to reach an optimal solution for 50 initializations with randomized obstacle locations. Note that four randomized initializations failed to reach an optimal solution prior to the final iteration. Average system overlap and convergence rate, respectively, for bitstream variants with (**g**, **h**) different proportions of 1’s, (**i**, **j**) different average run lengths, and (**k**, **l**) different 0–0 combination frequencies. System overlap was averaged across 30 SA initializations for each bitstream variant.

Next, the scale of the spatial optimization problem was varied to evaluate the performance of the SA accelerator simulation in sequentially larger solution spaces. For a fair comparison between different-sized landscapes, the maximum system coverage, i.e., the proportion of unit cells filled assuming no overlap between drones and obstacles, was kept constant by scaling the size of the drones and obstacles along with the grid dimensions. Similarly, the relative starting positions of the drones and obstacles were also kept constant. Figure 5a shows the number of iterations required to solve the base four-drone, three-obstacle constrained spatial optimization problem at scaling factors of 1, 2, 4, 8, 16, 32, and 64, representing grid sizes of 10 × 10, 20 × 20, 40 × 40, 80 × 80, 160 × 160, 320 × 320, and 640 × 640. Clearly, larger grid sizes necessitate a more granular search that requires additional iterations to reach an optimal solution. For comparison, the expected number of brute-force trials required to reach an optimal solution was also calculated for each four-drone, three-obstacle spatial optimization problem. Supplementary Fig. 22 shows both the current solution and the best solution as a function of trial number for the 10 × 10 system in a representative brute-force search. The search acceleration, defined as the ratio between the expected number of brute-force trials required to reach an optimal solution and the number of SA iterations required to reach an optimal solution, is shown in Fig. 5b. Clearly, the SA algorithm provides a significant search acceleration that increases as the size of the system increases. For the 10 × 10 problem, the search acceleration is ~1800×, while in the 640 × 640 problem, SA provides a search acceleration of $1.27\times {10}^{15}$.

Next, we expand the scope of the constrained spatial optimization problem by varying several system parameters and evaluating their impact on the convergence speed of the SA accelerator simulation. First, we consider the number of agents in the system at a constant total system coverage. The system coverage for the four-drone, three-obstacle system was modified to 100%, meaning that the entire spatial domain remains covered with an ideal placement solution. Note that the grid size was kept constant at 160 × 160. The number of iterations required to solve the three-obstacle spatial optimization problem using the annealing accelerator simulation and the expected number of brute-force trials required to reach an optimal solution are shown in Fig. 5c for drone quantities of 4, 16, and 64. The search acceleration for each of these scenarios is shown in Fig. 5d. Clearly, scaling the number of drones expands the solution space drastically by increasing the decision depth. The expected number of brute-force trials required to reach an optimal solution is calculated using Eq. 5 shown below:

5 $$BFT=\frac{{\left({L}_{{{{\rm{E}}}}}-{L}_{{{{\rm{D}}}}}+1\right)}^{2{N}_{{{{\rm{D}}}}}}}{{N}_{{{{\rm{D}}}}}!}$$

Here, $L_{{\mathrm{E}}}$ is the side-length of the environment, $L_{{\mathrm{D}}}$ is the side-length of the drone, and $N_{{\mathrm{D}}}$ is the number of drones. Additional discussion regarding the derivation of Eq. 5 is present in the “Methods” section. Clearly, the large decision depth causes a drastic increase in the expected number of brute-force trials required to reach a solution and in turn causes the search acceleration to increase significantly.

To this point, all discussed SA algorithms have converged to the ground state solution given an annealing schedule with sufficient iterations and an appropriate set of temperatures. The system overlap as a function of iteration for 10 random initializations of the four-drone, three-obstacle problem with an 80 × 80 grid size is shown in Fig. 5e. Due to the stochastic nature of the SA algorithm, each initialization follows a different path through the solution space before converging to an optimal solution. We now investigate the impact of randomized obstacle locations on the convergence speed of the simulation. Here, three obstacles were placed randomly in a four 4 × 4 drone system of size 10 × 10. Figure 5f shows a cumulative distribution plot of the number of iterations required to reach an optimal solution for 50 random initializations. Supplementary Video 2 shows the evolution of the generated solution for 10 of the random initializations. Interestingly, the SA algorithm is able to approach the optimal solution in almost all cases. In four random initializations, the algorithm concluded its annealing schedule of 360 iterations without reaching one of the global minima. Supplementary Video 3 shows the system evolution over all iterations for the four initializations where the SA algorithm did not converge to an optimal solution. In the first two cases, the system was close to an ideal solution. Here, extended iterations at the lowest temperature would eventually allow the system to converge to an ideal solution. In the third and fourth cases, the system is clearly trapped in a local minimum and must traverse high energy states to reach an optimal solution. Here, an extended annealing schedule that includes higher temperatures would be required for the system to reach an optimal solution. Nonetheless, these results indicate that the hardware-based annealing accelerator can perform in many generalized spatial optimization scenarios.

Next, we quantitatively assess the influence of deviations from ideal randomness on the annealing dynamics by artificially modifying the random bitstream used to guide drone selection and movement. First, we constructed eight non-ideal bitstream ensembles with proportions of ones ranging from 10% to 90%. These biased bitstreams were generated by randomly flipping bits from the original balanced sequence with ~50% ones to achieve the desired imbalance. For each of the nine bitstreams with varying proportions of ones, the annealing accelerator simulation was used to evaluate 30 different SA initializations on a representative 20 × 20 four-drone, three-obstacle system with 24 permissible solutions. Each initialization followed an annealing schedule comprising 120 iterations per temperature at six descending temperatures for a total of 720 iterations. For a fair comparison, we have used identical initial drone positions across all different bitstreams such that only the random bits used for optimization change between initializations. Convergence trajectories illustrating the system overlap averaged across all 30 initializations as a function of iteration number for each bitstream variation are shown in Fig. 5g. The convergence rate, defined as the percentage of initializations that reach a permissible solution, is shown as a function of the proportion of ones in the random bitstream in Fig. 5h. As expected, the convergence rate is maximized when the random bitstream contains an ideal ~50% proportion of ones. These results demonstrate that an intrinsically balanced bit distribution provides the most effective source of stochasticity for the annealing circuit, thereby enhancing convergence behavior and overall algorithm efficiency.

The average run length (or duration) of 0’s and 1’s in a bitstream is another important figure of merit for a random bitstream. For our TRNG bitstream, the mean run length of both 0’s and 1’s was measured to be ~2. Starting from this experimentally obtained metric, we synthetically generated four additional bitstreams with controlled average run lengths of 2.25, 2.5, 2.75, and 3, thereby spanning five total bitstream configurations. For each of these run-length configurations, we repeated the same annealing performance analysis described above. Figure 5i shows the overlap averaged across all 30 initializations as a function of iteration number for all five run-length bitstream configurations while Fig. 5j plots the convergence rate as a function of average run length. The bitstream with an average run length of 2, corresponding to our unadulterated TRNG bitstream, yields the largest number of converged solutions across 30 independent random initializations. Larger run lengths systematically reduce the convergence rate. These results highlight that introducing longer contiguous strings of 0’s and 1’s degrades the performance of the simulated annealing algorithm.

Next, the serial test investigates the frequency of occurrence of the 2^m^ different m-bit sequences. When *m* = 2, there are four 2-bit combinations: 0-0, 0-1, 1-0, and 1-1. For our TRNG bitstream, the frequency of occurrence of each 2-bit combination was nearly ideal at ~25%. We synthetically generated four additional modified bitstreams by varying the frequency of occurrence of the 0-0 combination to 30%, 35%, 40%, and 45%. These bitstreams were generated by randomly converting 0-1, 1-0, and 1-1 combinations into 0-0 combinations at equal proportions. For each of these 2-bit combination frequency configurations, we repeated the same annealing performance analysis described above. Figure 5k shows the overlap averaged across all 30 initializations as a function of iteration number for the five bitstream configurations, while Fig. 5l plots the convergence rate as a function of 0-0 combination frequency. The bitstream with an average 0-0 frequency of 25%, corresponding to our unadulterated TRNG bitstream, yields the largest number of converged solutions across 30 independent random initializations. Clearly, higher frequencies of 0-0 combinations yield reduced convergence rates. It is evident that true random numbers enable higher convergence rates than the biased bitstream configurations. These results highlight the necessity of high-quality TRNG bits.

Finally, we evaluate the impact of experimental error on SA convergence in systems ranging from size 10 × 10 to 320 × 320. As demonstrated previously, the TRNG, energy computation, and acceptance modules exhibit negligible error in small-scale experiments. We do not expect the error in the TRNG and energy computation modules to increase in larger systems because these circuits are entirely digital. While the acceptance module receives analog inputs, the gain of the three-stage inverter is sufficiently large that at a given switching threshold, the output of this module should exhibit little-to-no error as the system size increases. The largest source of computation error in the annealing accelerator is the analog summation module. During addition operations, the input terminal acts as the drain of the transistor. As the capacitor charges, the effective gate-to-source voltage (${V}_{{\mathrm{GS}}}$) progressively decreases. Conversely, during subtraction, the application of a negative voltage causes the input terminal to act as the source, resulting in a fixed ${V}_{{\mathrm{GS}}}$. As a consequence, addition and subtraction do not occur symmetrically for fixed back-gate voltages and input-voltage magnitudes.

The impact of summation error on the solution acceptance process largely depends on the ideal ΔH and the annealing temperature. In some cases, the ideal ΔH value is not close to the ${V}_{{\mathrm{SW}}}$ corresponding to the temperature prescribed by the annealing schedule. For example, if ${V}_{{\mathrm{SW}}}$ is 500 mV and the ideal ΔH is −250 mV, then a significant summation error of 750 mV would be required to alter the acceptance decision. More generally, in cases where the ideal ΔH is negative, error is not likely to affect the decision process. However, if the ideal ΔH is positive, it is more likely that the ideal ΔH and ${V}_{{\mathrm{SW}}}$ are similar in magnitude. Consequently, this increases the possibility that a proposed solution is incorrectly accepted or rejected.

We utilize the SA accelerator simulation to further investigate the impact of experimental summation error on solution acceptance and SA convergence. We consider summation error in the SA accelerator simulation by adding a summation variability term to the ideal ΔH. In our previously discussed simulation results, the probability of acceptance (${P}_{{{{\rm{Acceptance}}}}}$) for a proposed solution was ~100% if ${V}_{{\mathrm{SW}}} > \Delta H$ and ~ 0% if ${V}_{{\mathrm{SW}}}\le \Delta H$ following the thresholding function applied by the acceptance module. Considering finite summation error, ${P}_{{\mathrm{Acceptance}}}$ is now a function of ΔH, the system temperature, and the summation variability term, which is sampled from a normal distribution with a mean and standard deviation of ${\mu }_{{\mathrm{Sum}}}$ and ${\sigma }_{{\mathrm{Sum}}}$, respectively. Supplementary Fig. 23 shows ${P}_{{{{\rm{Acceptance}}}}}$ as a function ΔH for five different temperatures (${T}_{0}$ to ${T}_{4}$) with ${\mu }_{{{{\rm{Sum}}}}}=-0.041 \,{\mathrm{V}}$ and ${\sigma }_{{{{\rm{Sum}}}}}=0.24 \, {\mathrm{V}}$. Note that ${P}_{{{{\rm{Acceptance}}}}}$ at each ΔH and system temperature was determined numerically by collecting 10,000 samples from the variability distribution. ${P}_{{{{\rm{Acceptance}}}}}$ represents the proportion of solutions that satisfy ${V}_{{{\rm{SW}}}} > \Delta H+N({\mu }_{{{\rm{Sum}}}},{\sigma }_{{{\rm{Sum}}}}^{2})$. As expected, ${P}_{{{{\rm{Acceptance}}}}}$ reduces with increasing ΔH and changes in the system temperature cause a shift in the ${P}_{{{{\rm{Acceptance}}}}}$ curve.

The modified SA accelerator simulation was evaluated in a variety of system sizes (10 × 10, 20 × 20, 40 × 40, 80 ×  80, 160 × 160, and 320 × 320) with different magnitudes of ${\sigma }_{{{{\rm{Sum}}}}}$ ranging from 0 V to 1 V. The convergence rate, i.e., the proportion of SA initializations that converge to an ideal solution, was determined for a set of 100 SA initializations at each system size and ${\sigma }_{{\mathrm{Sum}}}$ combination. Supplementary Fig. 24a shows the convergence rate as a function of ${\sigma }_{{{{\rm{Sum}}}}}$. Clearly, the convergence rate decreases rapidly when ${\sigma }_{{{{\rm{Sum}}}}}$ surpasses ~0.3 V. Interestingly, there is modest variation in convergence rate across system sizes at a given ${\sigma }_{{\mathrm{Sum}}}$. In our hardware summation circuit, the experimental ${\sigma }_{{{{\rm{Sum}}}}}$ was 0.24 V, indicating that the experimental summation error should not significantly degrade the SA convergence rate. However, increased levels of summation error beyond ~0.3 V effectively degrade the convergence rate for all system sizes. The system coverage, defined as the ratio between the highest realized system coverage and the max possible system coverage, is a metric that can be utilized to evaluate the solution quality for a given SA initialization. The mean system coverage of the 100 SA initializations is shown for each system size and ${\sigma }_{{{{\rm{Sum}}}}}$ in Supplementary Fig. 24b. Here, increasing ${\sigma }_{{{{\rm{Sum}}}}}$ has a modest effect on mean system coverage. Mean system coverage also exhibits a weak dependence on system scale, remaining above ~95% across all grid sizes. Although large summation error can prevent convergence to the global maximum coverage, the best solution generated by the SA accelerator simulation is still likely to achieve high coverage.

Despite the minimal impact of experimental summation error on convergence rate, error mitigation techniques can be employed that may increase SA convergence rate. Here, we discuss an algorithmic error-mitigation strategy before considering a circuit-level mitigation strategy. First, the annealing schedule can be modified to increase the convergence rate. For an (A × A) system, we set the number of iterations per temperature to A*I, where I is a multiplicative factor. The 10 × 10 four-drone system with three obstacles that was evaluated in hardware was utilized to investigate the impact of I on convergence rate. The initial positions of all drones were kept constant across hardware and software initializations. The convergence rate is shown as a function of I in Supplementary Fig. 24c. The previously reported median convergence rate of 66% was obtained using I=3, corresponding to 30 iterations per temperature. Clearly, the convergence rate increases monotonically with I, indicating that higher convergence rates can be achieved by increasing the number of iterations per temperature. However, this increase in convergence rate comes at the expense of additional computation time and resources. Also note that the set of temperatures prescribed by the annealing schedule is another critical SA parameter. We utilized five descending temperatures for this analysis. Different temperature sets would likely yield varying results. For very large systems, a digital counter could be utilized as a more robust and scalable summation circuit. However, a digital counter would require an increase in transistor count relative to the current summation circuit.

In summary, we demonstrated hardware acceleration of SA for spatial optimization of constrained systems using CMOS circuits created with n-type MoS_2_ FETs and p-type WSe_2_ FETs. The four SA modules were used to implement random number generation, Boolean logic evaluation, summation, and tunable application of acceptance criteria. This hardware implementation solved a four-drone, three-obstacle optimization problem in 134 iterations with an energy expenditure of ~43.36 nJ/iteration, resulting in a search acceleration of ~1800×. Simulations corroborated these results by showing that the degree of search acceleration scales favorably with both system size and agent count, indicating greater efficiency in larger and more complex configurations.

## Methods

### Large area WSe_2_ film growth

The growth of monolayer WSe_2_ on 2 in. diameter c-plane sapphire was carried out in a metal-organic chemical vapor deposition (MOCVD) system equipped with a cold-wall horizontal reactor with an inductively heated graphite susceptor with gas-foil wafer rotation^52^. Tungsten hexacarbonyl (W(CO)_6_) was used as the metal precursor, while hydrogen selenide (H_2_Se) was the chalcogen source with H_2_ as the carrier gas. The W(CO)_6_ powder was maintained at 30 °C and 400 Torr in a stainless-steel bubbler. The synthesis of WSe_2_ is based on a multi-step process, consisting of nucleation, ripening, and lateral growth steps, which was described previously^53^. In general, the WSe_2_ sample was nucleated for 30 s at 850 °C, then ripened for 10 min at 850 °C and 10 min at 1000 °C, and then grown for 30 min at 1000 °C, which gives rise to a coalesced film across the entire 2″ wafer. During the lateral growth, the tungsten flow rate was set as 3.8 × 10^−3^ sccm, and the chalcogen flow rate was set as 75 sccm while the reactor pressure was kept at 200 Torr. After growth, the substrate was cooled in H_2_Se to 300 °C to inhibit the decomposition of the obtained WSe_2_ films.

### Large area monolayer MoS_2_ film growth

The growth of MoS_2_ monolayer film on 2 in. diameter c-plane sapphire substrates was carried out in a metal-organic chemical vapor deposition (MOCVD) system (10.60551/znh3-mj13) equipped with a cold-wall horizontal reactor with an inductively heated graphite susceptor with gas-foil wafer rotation^52^. The Molybdenum hexacarbonyl (Mo(CO)_6_) (99.99%, Sigma-Aldrich) was used as the metal precursor, while hydrogen sulfide (H_2_S) was the chalcogen source with H_2_ as the carrier gas. The Mo(CO)_6_ powder was maintained inside a stainless-steel bubbler where the temperature and pressure of the bubbler were held at 20 °C and 625 Torr, respectively. The MoS_2_ monolayer was grown in a single-step process described previously^54^. Before the growth, the sapphire was ramped up under H_2_ to the growth temperature at 950 °C and pre-annealed for 10 min. During the growth, H_2_S and Mo(CO)_6_ were introduced to the reactor for a designated time to complete MoS_2_ monolayer growth in a single step. The molybdenum flow rate was set as 3.4 × 10^−3^ sccm and the chalcogen (H_2_S) flow rate was set as 400 sccm while the reactor pressure was maintained at 50 Torr. Then, the MoS_2_ monolayer was annealed under H_2_ and H_2_S ambient for 10 min at 950 °C before cooling down to inhibit the decomposition of the obtained MoS_2_ film. Using this condition, the growth of a fully coalesced monolayer MoS_2_ was achieved in 10–30 min across the 2 in. sapphire substrate.

### Fabrication of MoS_2_ and WSe_2_ transistors

Arrays of back-gate electrodes consisting of 2 nm Ti and 18 nm Pt were patterned and deposited on a p^++^-Si/SiO_2_ substrate. Next, a back-gate dielectric comprising 10 nm HfO_2_ was deposited via atomic layer deposition (ALD) for both p-type WSe_2_ FETs and n-type MoS_2_ FETs. Note that for memtransistors in the TRNG and acceptance function modules, a back-gate dielectric comprising 50 nm Al_2_O_3_ was utilized. For the fabrication of WSe_2_ FETs, a MOCVD-grown multilayer WSe_2_ film was first wet-transferred^55^ onto the substrate. The WSe_2_ channels were then patterned using electron beam (ebeam) lithography, followed by a dry etch using sulfur hexafluoride (SF_6_). Subsequently, source and drain metal contacts, consisting of 20 nm Pd and 30 nm Pt, were deposited using ebeam evaporation. A 50 nm thick Al_2_O_3_ capping layer was then ebeam-evaporated to physically isolate the WSe_2_ channels from the MoS_2_ channels during the fabrication of MoS_2_ FETs, ensuring proper device functionality and performance. The fabrication process for MoS_2_ FETs is similar to that of the WSe_2_ FETs, differing mainly in the material for wet transfer and the source/drain contact metals, which are MOCVD-grown monolayer MoS_2_ film and 30 nm Ni/15 nm Au metal contacts, respectively. After successfully fabricating both PMOS and NMOS devices on the same p^++^-Si/SiO_2_ substrate, circuit connections (Ti/Au) and an Al_2_O_3_ insulating layer at the crosspoints were deposited via e-beam evaporation.

### Electrical characterization

A Lake Shore CRX-VF probe station and a Keysight B1500A parameter analyzer were used for all electrical characterization of fabricated transistors and circuits. Electrical characterization was performed in atmospheric conditions.

### Raman spectroscopy

A Horiba LabRAM HR Evolution confocal Raman microscope with a 532 nm laser was used for Raman spectroscopy. The power of the laser was 34 mW filtered at 1% to 0.34 mW. The objective magnification was 100×. A grating with 1800 g/mm was used.

### Energy calculation

Energy consumption was calculated per movement of a single drone for each module. For the TRNG module, the energy consumption is 30 pJ/bit^32^. We require 4 random bits for each movement of the drone: 2 bits for selecting the moving drone [D1–D4] and 2 bits for the suggested movement [up, down, left or right]. Thus, the overall energy required for one movement of the drone is ${E}_{{\rm{TRNG}}}=120 \,{\rm{{pJ}}/{movement}}$.

In the energy computation module energy was calculated using $E=V\times I\times t$ where V is the supply voltage applied to the logic gate, I is the average drain current for one operation of the logic gate, and t is the time in seconds required for one operation. From hardware experiments, it was estimated that NAND gates consume approximately 5 pJ per operation and NOR gates consume approximately 1.6 pJ per operation at an operating voltage of 3 V as per the previous equation. The approximate energy consumption for the environment computation submodule which has 1 NAND and 2 NORs is ${E}_{{{{\rm{Env}}}}\_{{{\rm{NAND}}}}}=1 \times {\mbox{5\,pJ}}={5\,pJ}/{{\rm{operation}}}$ and ${E}_{{{{\rm{Env}}}}\_{{{\rm{NOR}}}}}=2 \times {\mbox{1.6\,pJ}}={\mbox{3.2\,pJ}}/{{\rm{operation}}}$. Thus, for one motion of the drone we require 100 such operations in the environment computation submodule, which is ${E}_{{{{\rm{Env}}}}}=100\times \left({\mbox{5\,pJ}}+{\mbox{3.2\,pJ}}\right)={\mbox{0.82 \,nJ}}/{\rm{movement}}.$ The final overlap computation submodule comprises 2 NANDs and 2 NORs. Thus, ${E}_{{{{\rm{Overlap}}}}\_{{{\rm{NAND}}}}}=2 \times {\mbox{5\,pJ}}={\mbox{10\,pJ}}/{{\rm{operation}}}$ and ${E}_{{{{\rm{Overlap}}}}\_{{{\rm{NOR}}}}}=2 \times {\mbox{1.6\,pJ}}={\mbox{3.2\,pJ}}/{{\rm{operation}}}$. The overlap computation gates will consume a total of ${E}_{{{{\rm{Overlap}}}}}=100\times \left({\mbox{10\,pJ}}+{\mbox{3.2\,pJ}}\right)={\mbox{1.32\,nJ}}/{{\rm{movement}}}$. The overall energy consumption of the energy computation module is ${E}_{{{{\rm{Energy}}}}\_{{{\rm{Computation}}}}}=E_{{{{\rm{Env}}}}}+{E}_{{{{\rm{Overlap}}}}} \approx {\mbox{2.14\,nJ}}/{{\rm{movement}}}$.

The summation module uses a combination of a capacitor and an ${\rm{MoS}}_{2}$ device. The average energy consumption of the capacitor is calculated using the equation ${E}_{{{{\rm{Capacitor}}}}}=C{{V}_{{\rm{Capacitor}}}}{{V}_{{\rm{DD}}}}$.

${E}_{{{{\rm{Capacitor}}}}}={\mbox{3\,nJ}}$ for a ${\mbox{1 \,nF}}$ capacitor charged to 1 V with an operating voltage of 3 V. Thus, the maximum overall energy consumed by the capacitor is ${\mbox{3\,nJ}}/{{\rm{movement}}}$. The overall energy consumption of the summation module is ${E}_{{{{\rm{Summation}}}}}={E}_{{{{\rm{Capacitor}}}}}={\mbox{3\,nJ}}/{{\rm{movement}}}$.

The acceptance module uses a three-stage NMOS inverter with an energy consumption of ${E}_{{\rm{Inv}}}\approx {\mbox{38.1\,nJ}}/{{\rm{operation}}}$. One operation is required per drone movement. The overall energy consumption of the acceptance module is ${E}_{{{{\rm{Accept}}}}}\approx{\mbox{38.1\,nJ}}/{{\rm{movement}}}$.

### Simulation of annealing accelerator

The SA accelerator simulation models the operations performed by each circuit module. First, pseudo-random numbers dictating the moving drone and its movement direction are generated to replicate the TRNG module. Next, logic Eqs. 1–3 are used to perform the energy computation. Subsequently, Eq. 4 is used to represent the summation module. Finally, the output of the acceptance module, ${V}_{{\rm{AM}}}$, is modeled using the following equation:

6 $${V}_{{{{\rm{AM}}}}}=\frac{{V}_{{{{\rm{DD}}}}}}{1+{e}^{{a}({V}_{{{{\rm{IN}}}}}-{V}_{{{{\rm{SW}}}}})}}$$

Here, ${V}_{{{{\rm{DD}}}}}$ is the supply voltage, ${V}_{{{{\rm{IN}}}}}$ is input voltage, ${V}_{{{{\rm{SW}}}}}$ is the switching threshold of the inverter, and a is a fitting parameter that is modified to emulate the approximate gain of a three-stage inverter. The annealing schedule was modified to allow the agents to adequately explore solutions in each system. For scaling of the four-drone, three-obstacle system, six temperatures were utilized, with six times the side length of the grid dictating the number of iterations per temperature. The same annealing schedule rule was used for the 10 random initializations of the four-drone, three-obstacle system at a system size of 80 × 80 and for the 50 randomly placed obstacle initializations in the four-drone, three-obstacle system. While scaling the number of drones, six temperatures were utilized, with the product of 6, the side length of the grid, the grid size scale factor, and the number of drones dictating the number of iterations per temperature.

### Derivation of Eq. 5

Here, we provide additional commentary regarding the derivation of Eq. 5. First, we estimate the number of brute-force trials required to fully explore the solution space of the combinatorial problem under consideration. For clarity, we focus on the simplest scenario: a square grid populated by square drones. Let ${L}_{{{{\rm{E}}}}}$ denote the side-length of the environment, L_D_ the side-length of a drone, and ${N}_{{{{\rm{D}}}}}$ the total number of drones. These quantities are connected through the straightforward geometric relation:

7 $${L}_{{{{\rm{D}}}}}=\lfloor\frac{{L}_{{{{\rm{E}}}}}}{\sqrt{{N}_{{{{\rm{D}}}}}}}\rfloor$$

Moreover, we enforce three additional constraints: (1) drones are aligned with the grid axes, i.e. no rotation, (2) no obstacles in the grid, and (3) only integer positions can be assigned. These rules enforce the possible positions of the drones to the following coordinates:

8 $$\left\{\begin{array}{c}0 < x \leq {L}_{{{{\rm{E}}}}}-{L}_{{{{\rm{D}}}}}+1\\ 0 < y \leq {L}_{{{{\rm{E}}}}}-{L}_{{{{\rm{D}}}}}+1\end{array}\right.$$

We can conclude that we have $({L}_{{{{\rm{E}}}}}-{L}_{{{{\rm{D}}}}}+1)({L}_{{{{\rm{E}}}}}-{L}_{{{{\rm{D}}}}}+1)$ possible positions in the grid for each drone. Considering N_D_ different drones, the number of combinations becomes:

9 $${\prod }_{1}^{{N}_{{{{\rm{D}}}}}}{\left({L}_{{{{\rm{E}}}}}-{L}_{{{{\rm{D}}}}}+1\right)}^{2}={\left({L}_{{{{\rm{E}}}}}-{L}_{{{{\rm{D}}}}}+1\right)}^{2N_{{\rm{D}}}}$$

Next, dividing by the number of degenerate solutions, i.e., differing permutations of ${N}_{{{{\rm{D}}}}}$ drones exchanging positions, we get $\frac{{\left({L}_{{{{\rm{E}}}}}-{L}_{{{{\rm{D}}}}}+1\right)}^{2{N}_{{{{\rm{D}}}}}}}{{N}_{{{{\rm{D}}}}}!}$, which is the expected number of brute force trials required to reach an optimal solution. This formula does not account for collisions (overlaps) between drones, so it represents the total space of *possible* configurations rather than the space of *valid* solutions. Supplementary Fig. 25 shows the expected number of brute-force trials required to find an optimal solution for different allowable combinations of ${L}_{{{{\rm{E}}}}}$, ${L}_{{{{\rm{D}}}}}$ and ${N}_{{{{\rm{D}}}}}$. We clearly observe a rapidly growing trend as the number of drones increases while the grid side-length remains fixed. The discontinuities in the plot arise from numerical overflow in MATLAB. To understand the behavior in that region, we can simplify the expression by noting that when the drone size, ${L}_{{{{\rm{D}}}}}=1$, the maximum number of drones that can fit in an unobstructed grid (no obstacles) corresponds to the grid’s side length. Under this assumption, the equation reduces to:

10 $$\frac{{\left({L}_{{{{\rm{E}}}}}-1+1\right)}^{2{L}_{{{{\rm{E}}}}}^2}}{{L}_{{{{\rm{E}}}}}^2!}=\frac{{x}^{2x^2}}{x^2!}\sim \frac{{e}^{x^2}}{x\surd (2\pi )}$$

Which clearly demonstrates an unacceptable increase in the expected number of brute-force trials required to find an optimal solution.

## Supplementary information

Supplementary Information

Supplementary Video 1

Supplementary Video 2

Supplementary Video 3

Transparent Peer Review file

## Data Availability

Data on samples produced in the 2DCC-MIP facility, including growth recipes and characterization data, are available at 10.26207/f9fx-db95. Other data generated during and/or analyzed during the current study are available from the corresponding author on request.

## Code availability

The codes used for data analysis are available from the corresponding author on request.
