# Supplementary material for: Hardware acceleration of simulated annealing for constraint satisfaction problems
Source: Nat Commun. 2026 Jul 21;17:8905. doi: 10.1038/s41467-026-74893-1 (PMC13503799; doi:10.1038/s41467-026-74893-1)
Supplement: Supplementary file 1 [file 41467_2026_74893_MOESM1_ESM.pdf]

Hardware Acceleration of Simulated Annealing for Constraint Satisfaction Problems

Supplementary Information

Andrew Pannone[1#], Rishikesh T. Nair[1#], Pranav Krishnan[1], Fabio Carletti[2], Harikrishnan Ravichandran[1], Priyatam Gundapuneni[3], Arav Dutta[1], Subir Ghosh[1], Chen Chen[4], Joan M. Redwing[4,5,6,7] & Saptarshi Das[1,4,5,6,7]*

[1]*Engineering Science and Mechanics, Penn State University, University Park, PA 16802, USA*

[2]*Elettronica, Informazione e Bioingegneria, Politecnico di Milano, Milan 20133, Italy*

[3]*Mechanical Engineering, Penn State University, University Park, PA 16802, USA*

[4]*2D Crystal Consortium Materials Innovation Platform, Penn State University, PA 16802, USA*

[5]*Materials Science and Engineering, Penn State University, University Park, PA 16802, USA*

[6]*Materials Research Institute, Penn State University, University Park, PA 16802, USA*

[7]*Electrical Engineering, Penn State University, University Park, PA 16802, USA*

[*]*Corresponding author: email – sud70@psu.edu*

[#]*These authors contributed equally*

Supplementary Figure 1

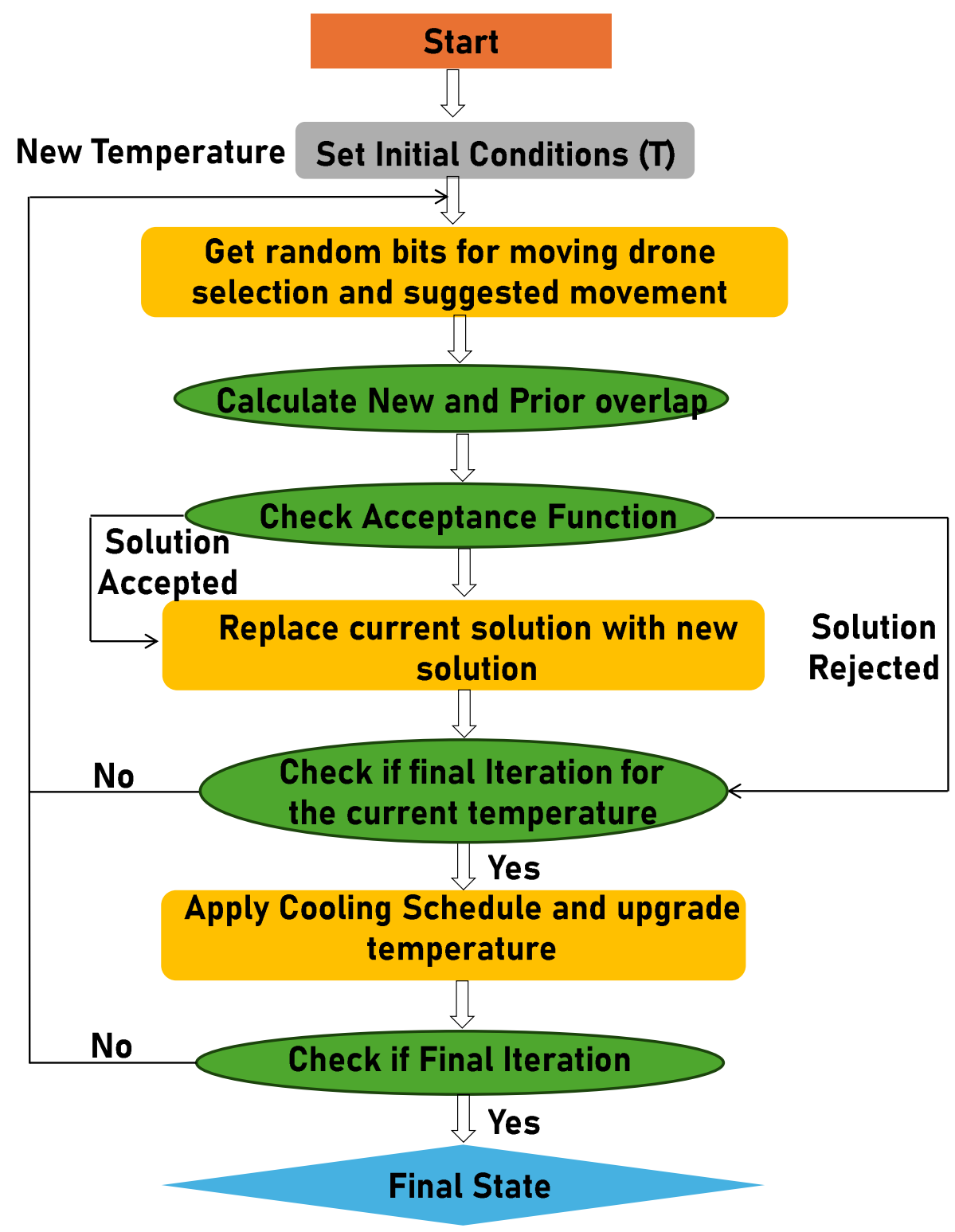

Supplementary ***Fig. 1. SA algorithm flowchart.*** *Flowchart illustrating the progression of the simulated annealing algorithm from the initial state to the final optimized solution. The process includes iterative exploration, probabilistic acceptance of suboptimal solutions to escape local minima, and gradual temperature reduction to ensure convergence toward the global optimum.*

Supplementary Figure 2

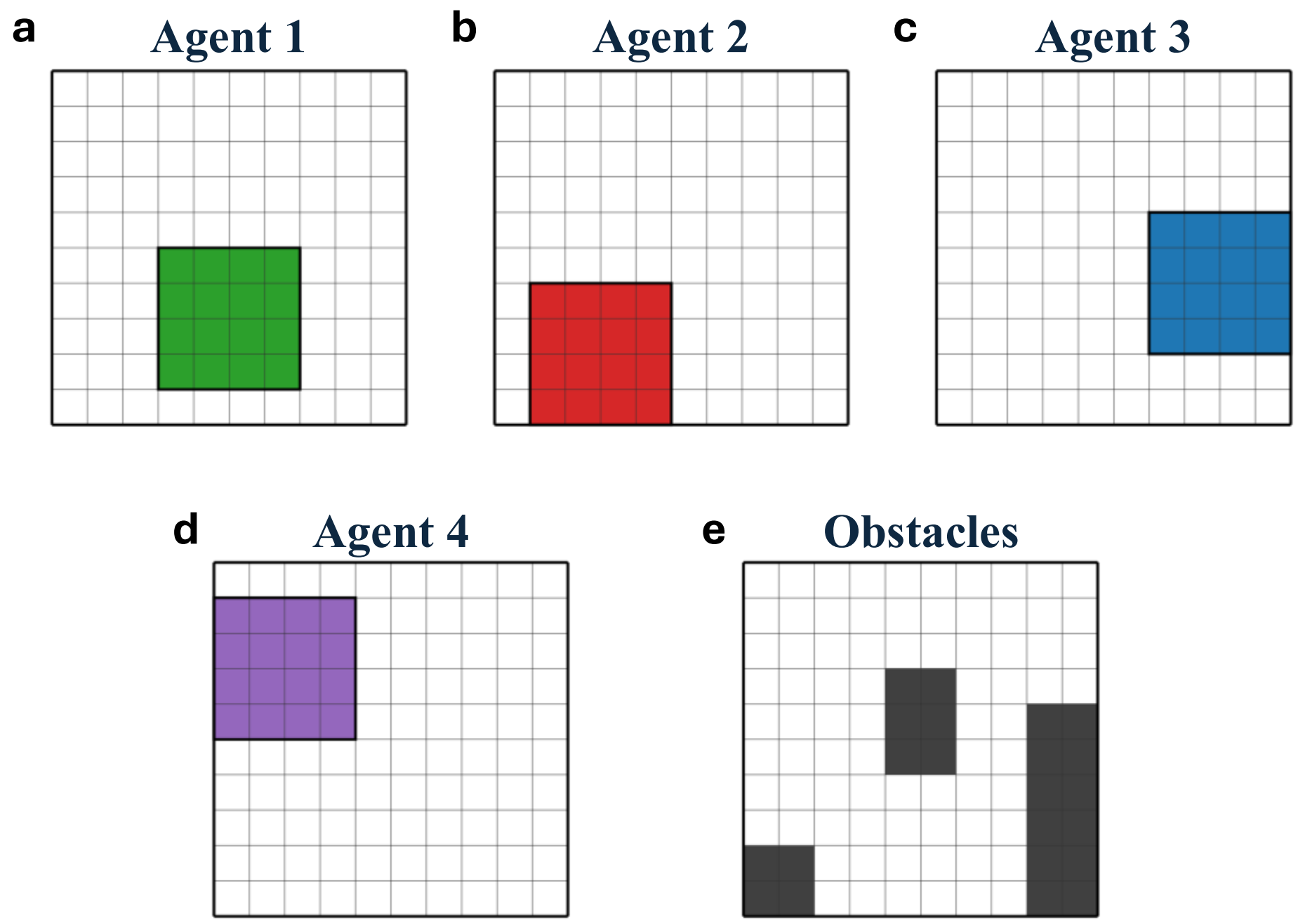

Supplementary ***Fig. 2. Occupancy maps of agents and obstacles. (a-d)*** *Occupancy maps of agents 1, 2, 3, and 4.* ***(e)*** *Occupancy map of three obstacles.*

Supplementary Figure 3

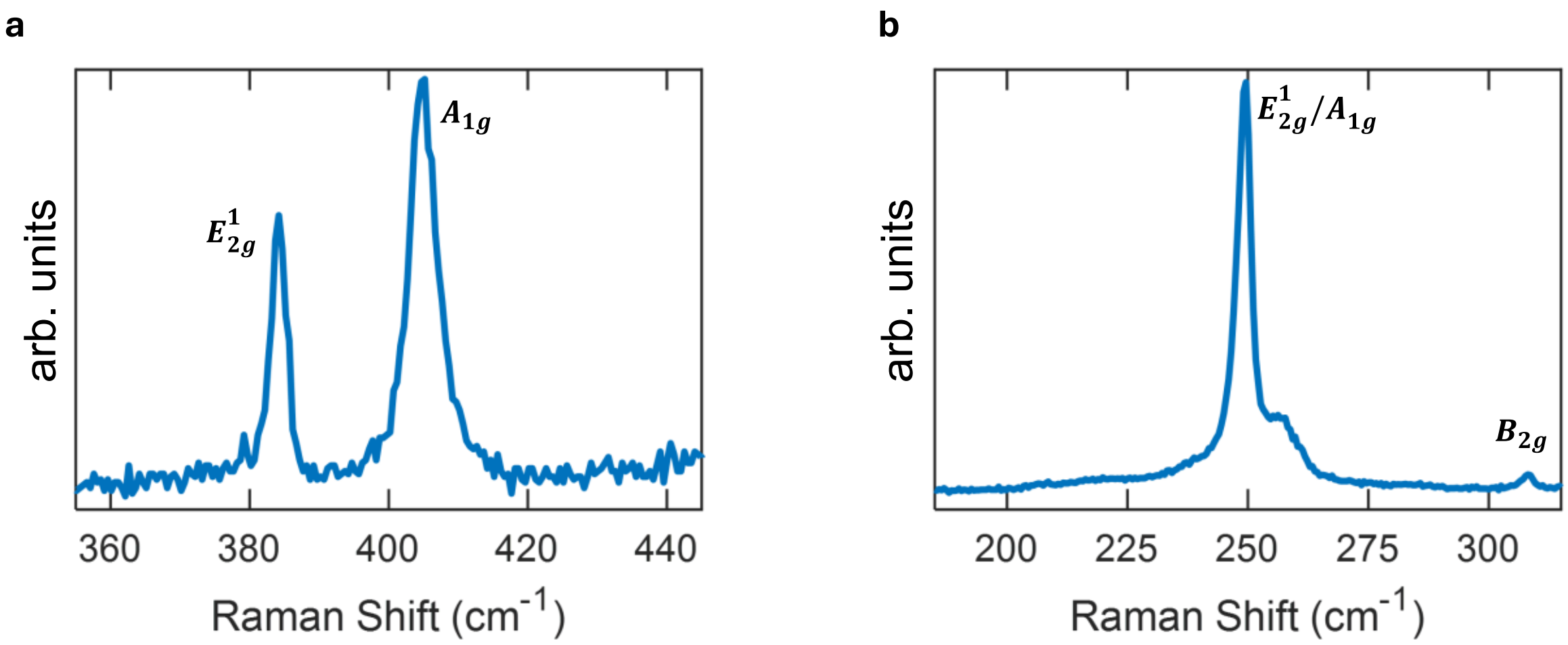

Supplementary ***Fig. 3. Raman Spectra.*** *Raman spectra of* ***(a)*** *MoS_2 and* ***(b)*** *WSe_2 films.*

Supplementary Figure 4

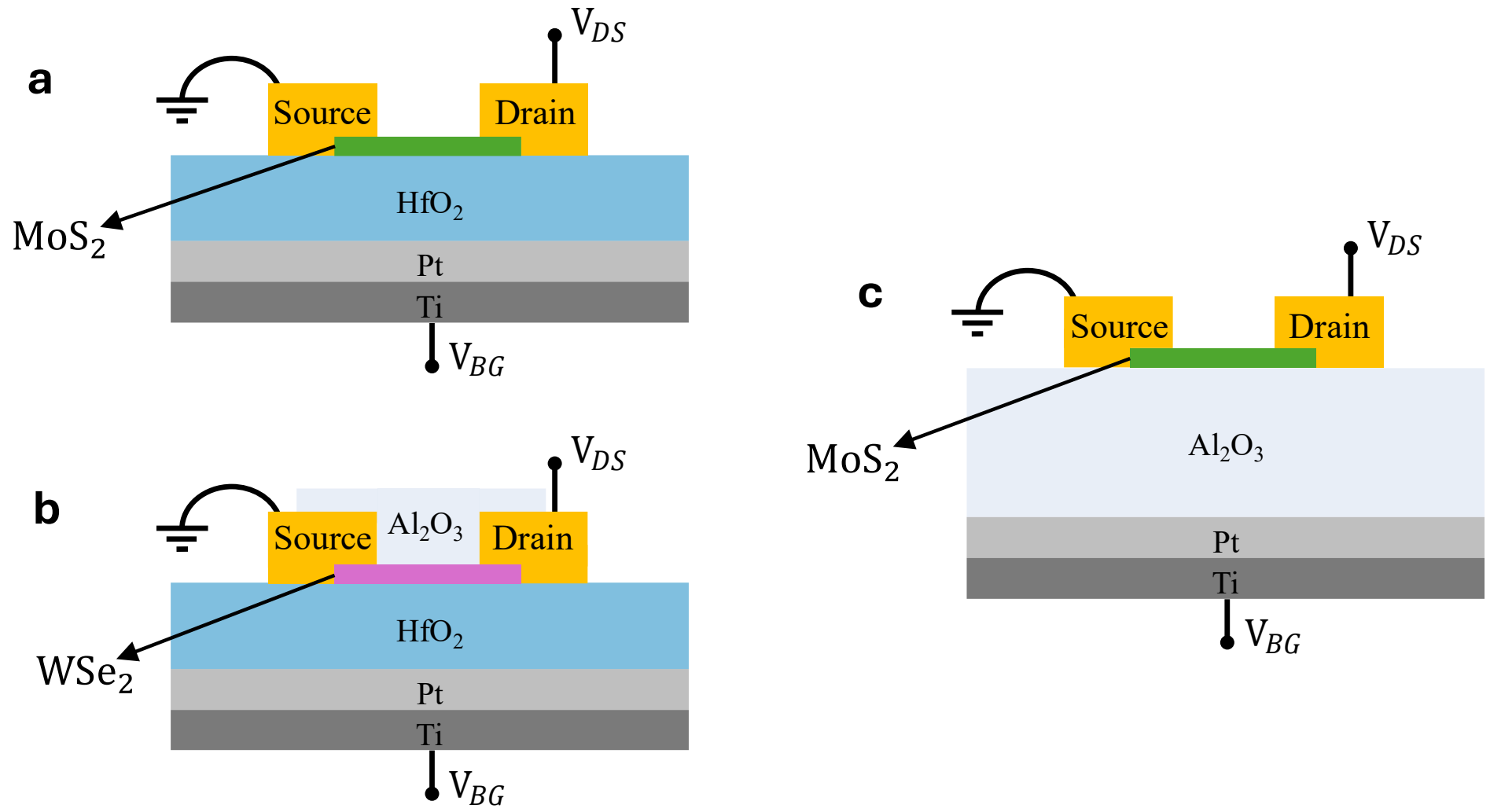

Supplementary *Fig. 4. Cross-Sectional Schematics of memtransistors.* *Schematic diagrams of **(a)** MoS_2 and **(b)** capped WSe_2 FETs used for the CMOS logic gates. **(c)** Schematic diagram of MoS_2 memtransistors used for the acceptance module. Programmability of the MoS_2 memtransistors is attributed to charge trapping at or near the dielectric-MoS_2 interface.*

Supplementary Figure 5

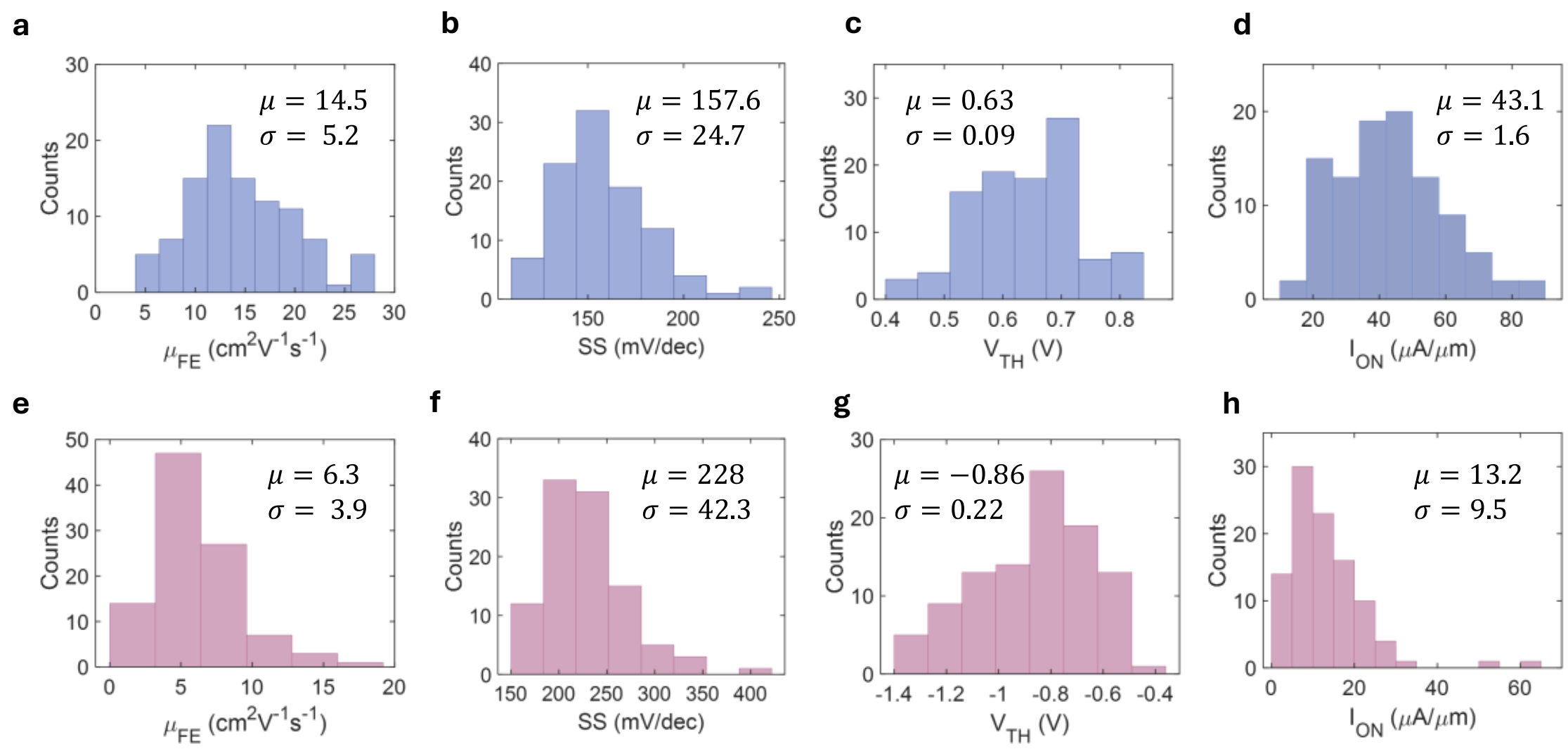

Supplementary Fig. 5. FET performance statistics. (a) *Carrier mobility,* ***(b)*** *subthreshold slope,* ***(c)*** *threshold voltage and* ***(d)*** *ON-current of 100 MoS_2 FETs.* ***(e)*** *Carrier mobility,* ***(f)*** *subthreshold slope,* ***(g)*** *threshold voltage and* ***(h)*** *ON-current of 100 WSe_2 FETs.*

Supplementary Figure 6

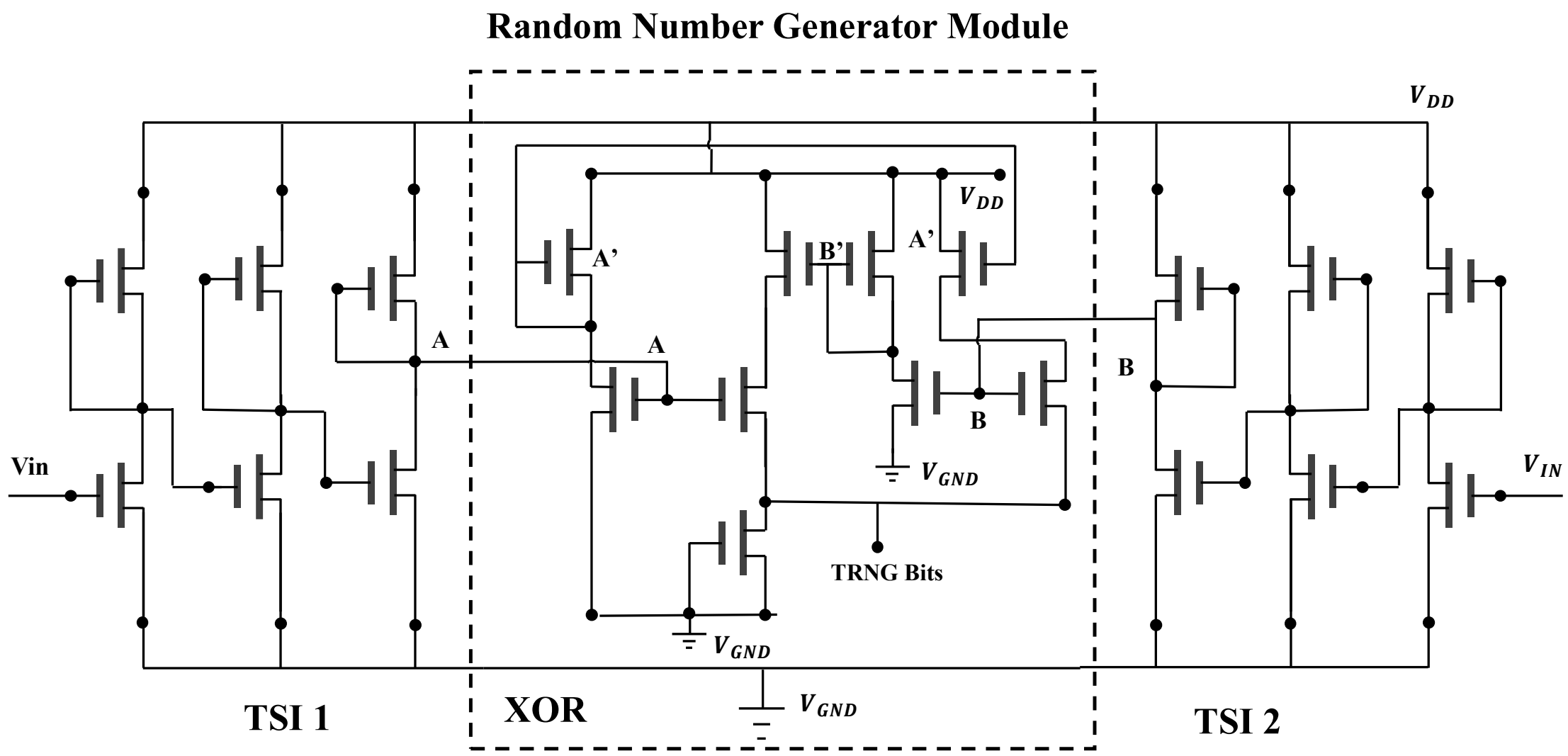

Supplementary ***Fig. 6. Transistor level Circuit Diagram of TRNG Module.*** *Transistor-level schematic of the* MoS_2*-based true random number generator (TRNG) module. The circuit comprises two three-stage inverters and a single XOR gate.*

Supplementary Figure 7

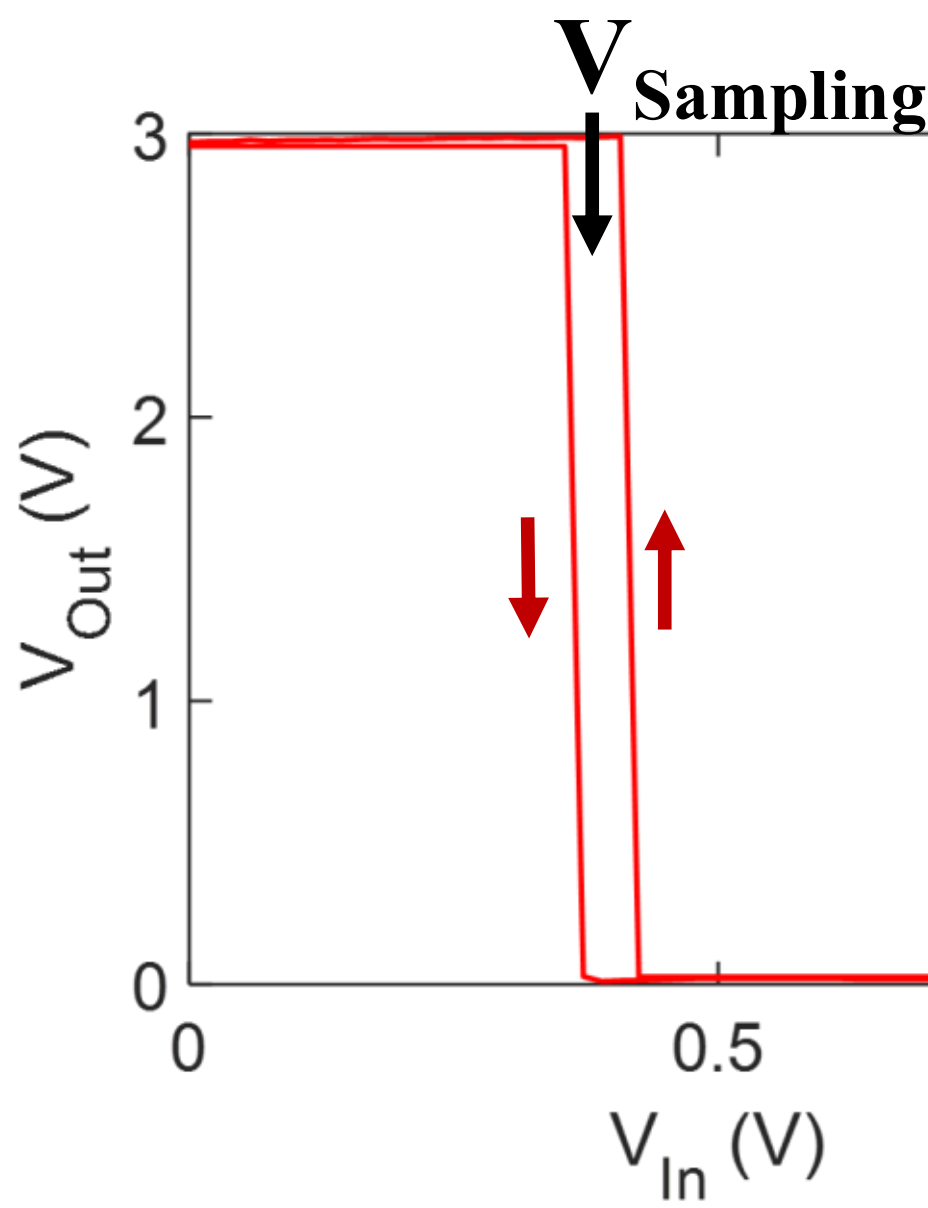

Supplementary *Fig. 7. Voltage transfer characteristics of a three-stage inverter.* *Output voltage, V_{Out}, measured as a function of input voltage, V_{In}, for a three-stage inverter. Random temporal fluctuations can be observed by sampling the three-stage inverter at an appropriate input voltage, $V_{Sampling}$, within the hysteresis window between the forward and reverse voltage sweeps.*

Supplementary Figure 8

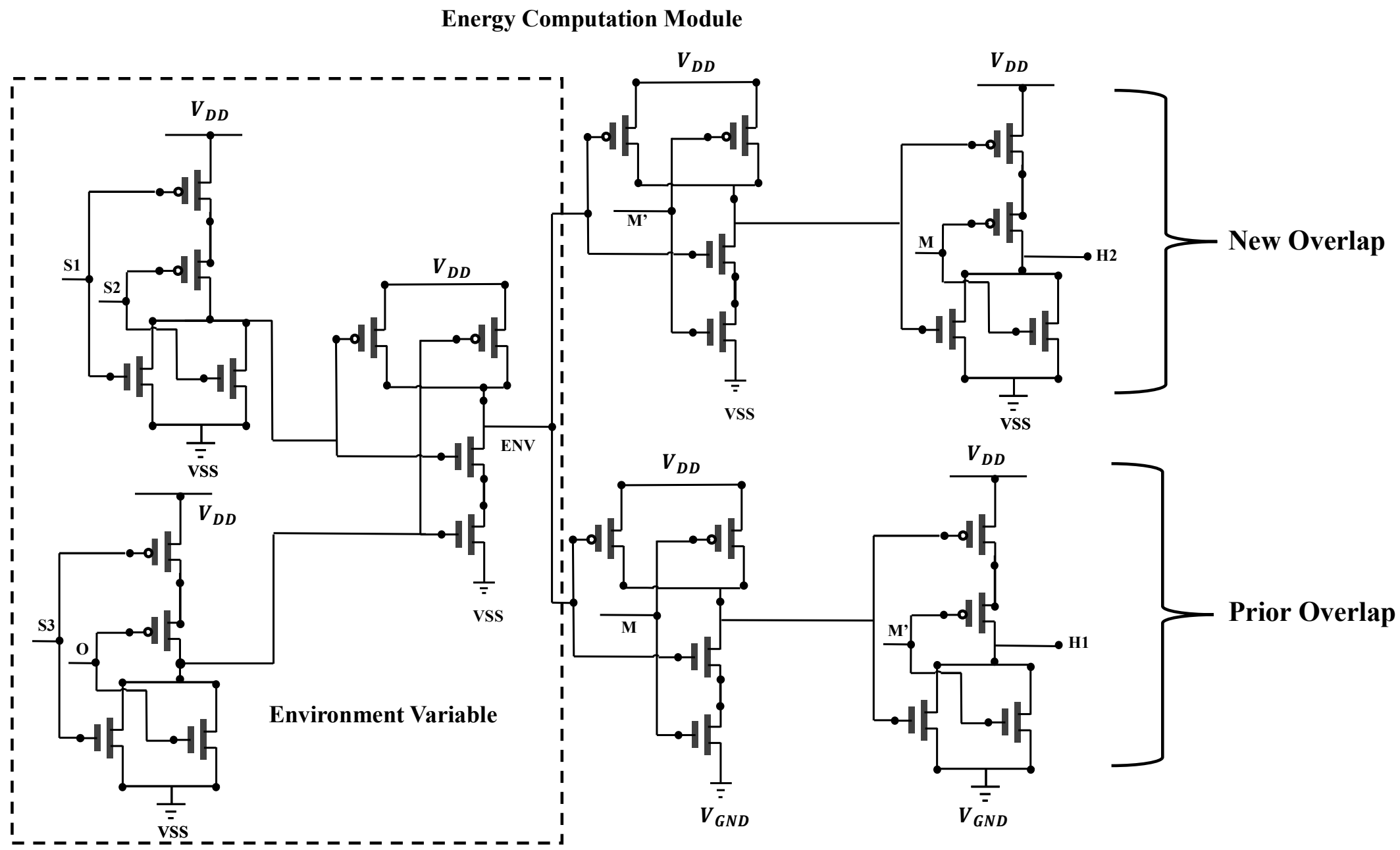

Supplementary *Fig. 8. Transistor level Circuit Diagram of energy computation module.* *Transistor-level schematic of the energy computation module, comprising exclusively NAND and NOR gates.*

Supplementary Figure 9

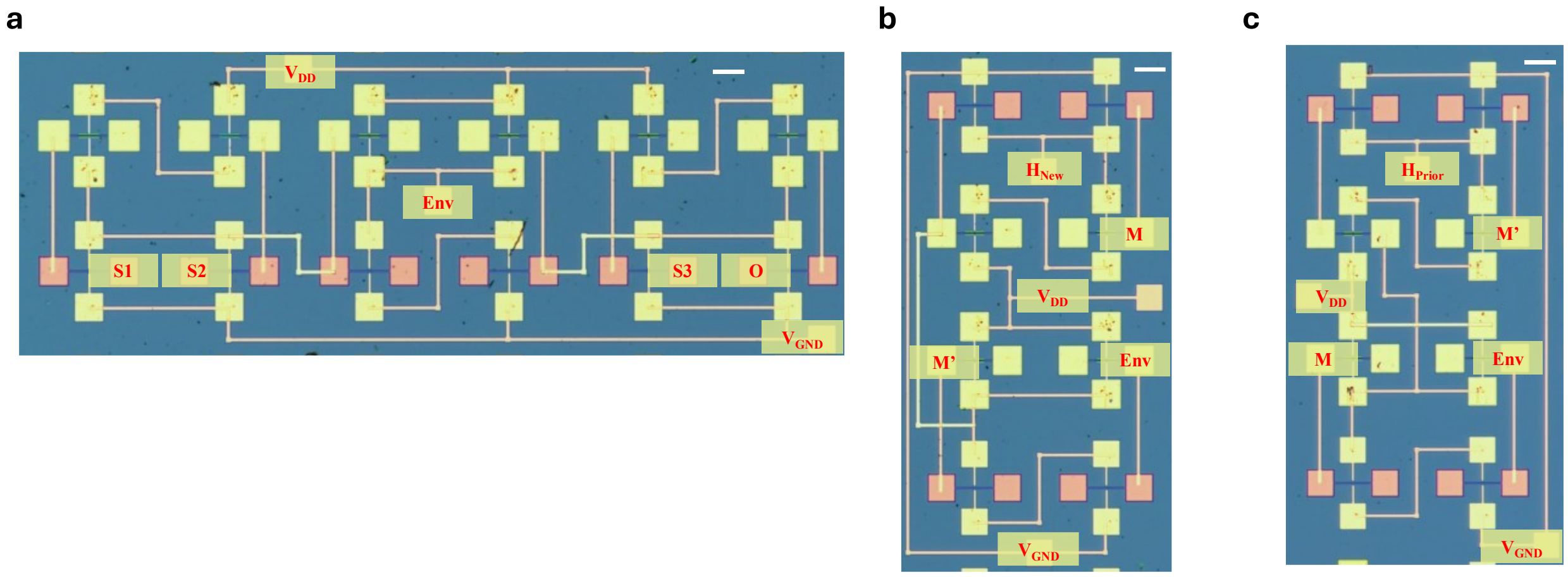

Supplementary *Fig. 9 Optical images of environment and overlap computation submodules.* ***(a)*** *An optical image of the environment computation submodule that consists of two NOR gates and one NAND gate which receives* S_1, S_2, S_3, *and* O *as its inputs and generates* Env *as its output. (scale bar: 40 µm). Optical images of the* ***(b)*** *new and* ***(c)*** *prior overlap computation submodules both consisting of one NAND and one NOR gate. The new overlap and prior overlap submodules both take* M, M', *and* Env *as inputs and compute* H_{New} *and* H_{Prior}, *respectively (scale bar: 40 µm).*

Supplementary Figure 10

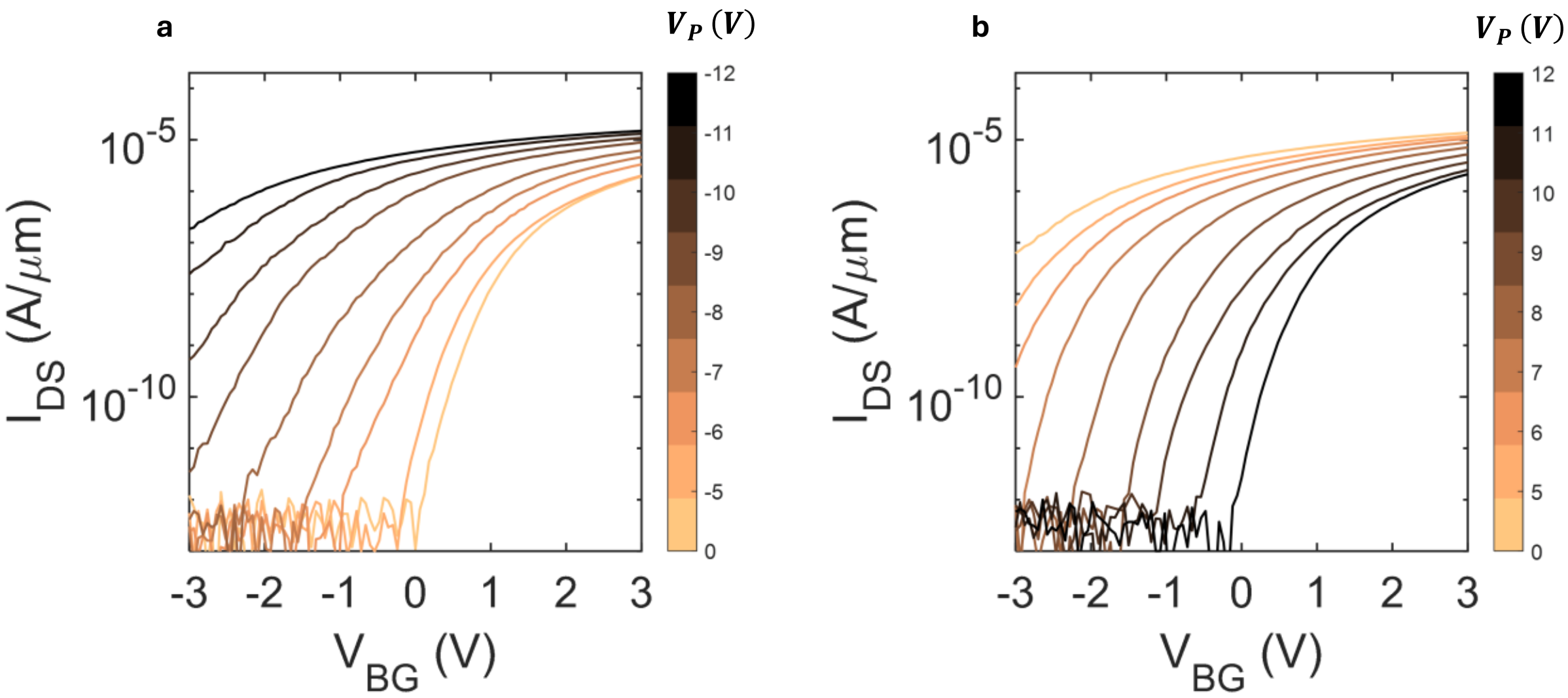

Supplementary ***Fig. 10. Programmability of MoS_2 Memtransistors. (a)*** *Transfer characteristics of a representative MoS_2 memtransistor in an initial state (denoted by $V_P = 0\,V$) and after the application of negative programming voltage pulses (V_P) of varying integer magnitudes.* ***(b)*** *Transfer characteristics of a representative MoS_2 memtransistor in an initial state (denoted by $V_P = 0\,V$) and after the application of positive programming voltage pulses (V_P) of varying integer magnitudes. All pulses were applied for a constant duration of 100 ms.*

Supplementary Figure 11

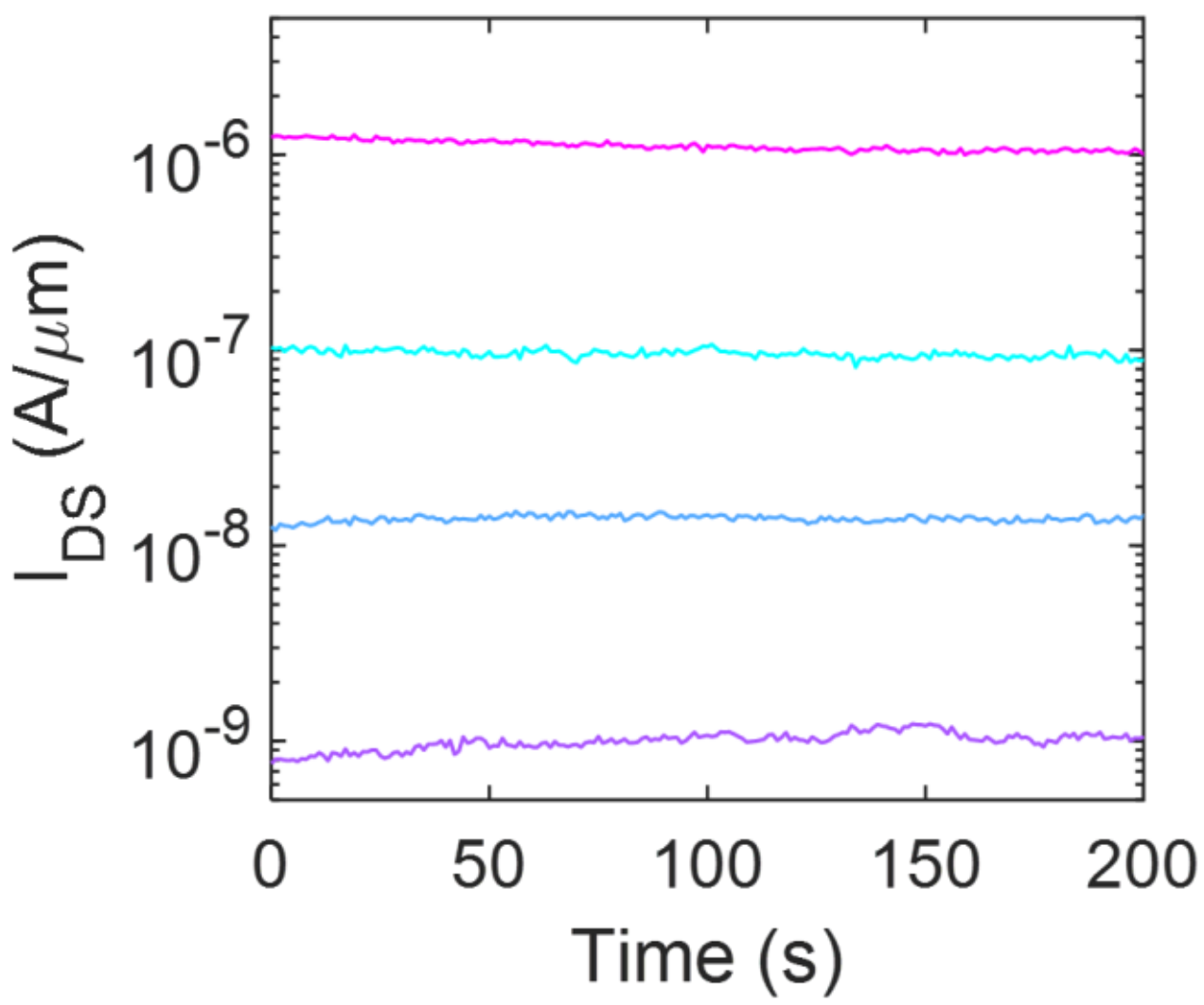

Supplementary ***Fig. 11. Retention of programmed states in MoS_2 Memtransistors.*** *Drain-to-source current of a representative MoS_2 memtransistor measured at a back-gate voltage of 0 V for 200 s after it was programmed into four different conductance states.*

Supplementary Figure 12

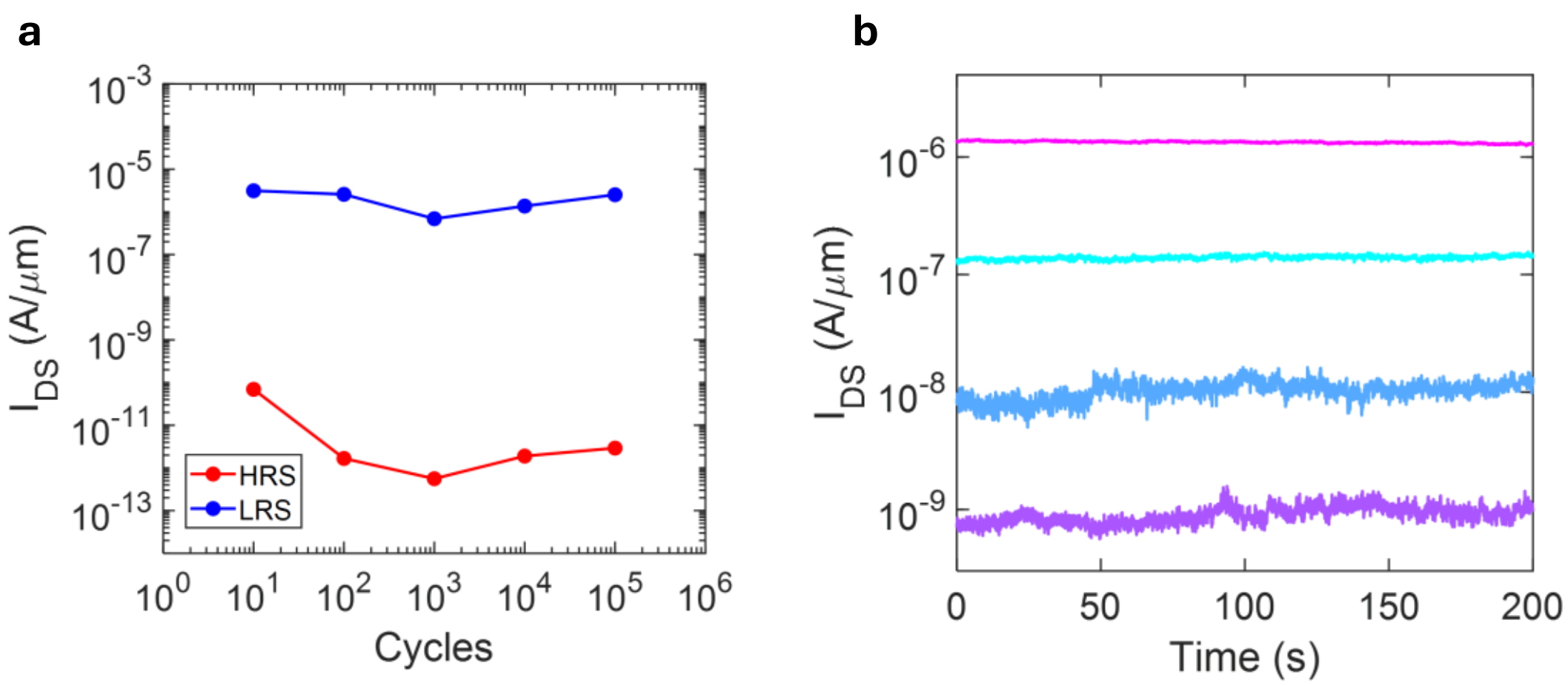

Supplementary *Fig. 12. Endurance and post-endurance retention of programmed states in MoS_2 Memtransistors.* *(a)* *Evolution of low-resistance state (LRS) and a high-resistance State (HRS) over 10^5 programming cycles.* ***(b)*** *Drain-to-source current of a representative MoS_2 memtransistor measured at a back-gate voltage of 0 V for 200 s after it was programmed into four different conductance states. Retention of memory states was measured after the endurance experiment.*

Supplementary Figure 13

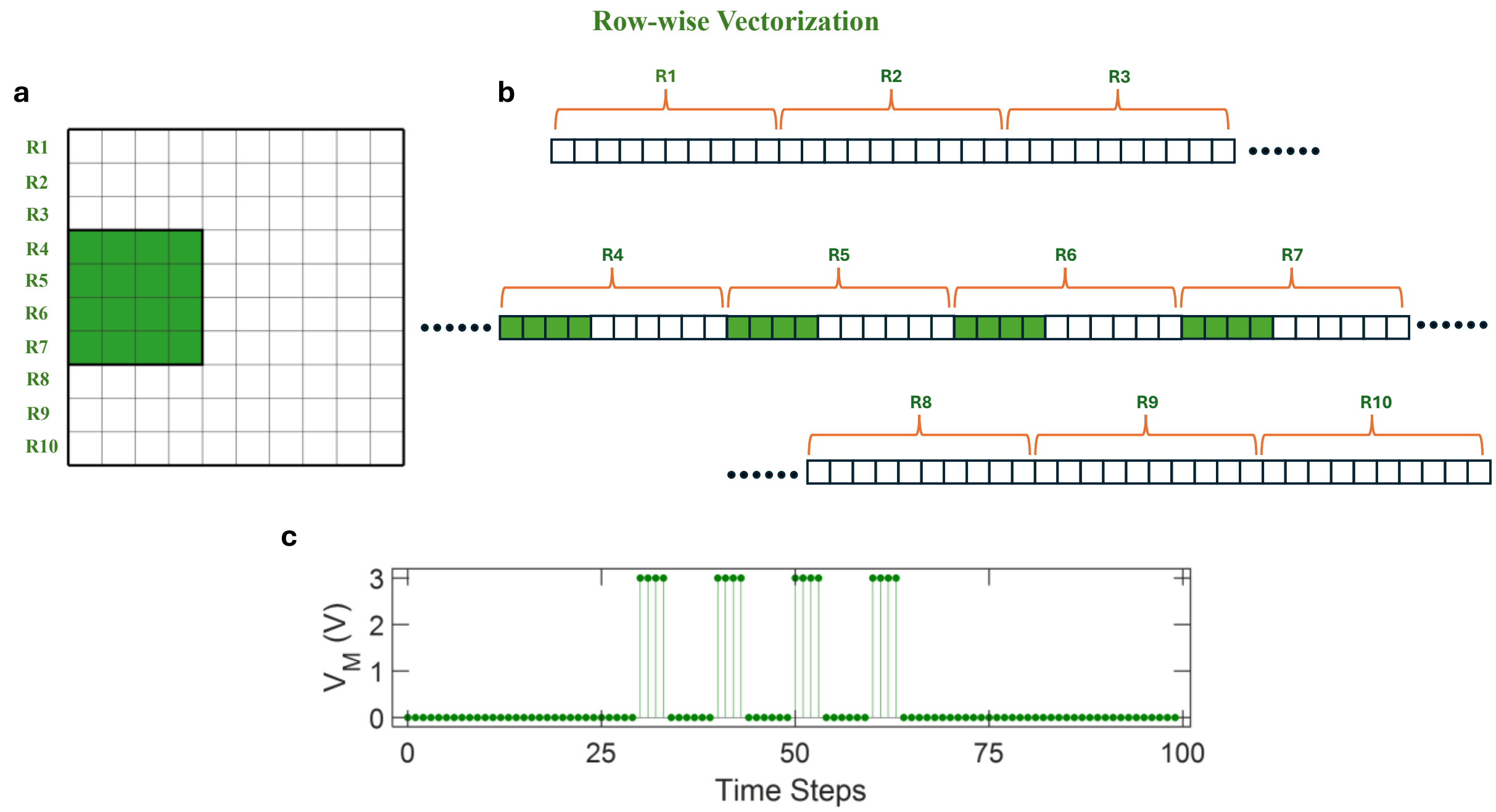

Supplementary ***Fig. 13. Drone map vectorization. (a)*** *10×10 map of a representative 4×4 drone.* ***(b)*** *Row-wise vectorization of the 10×10 map.* ***(c)*** *Stem plots corresponding to row-wise vectorized drone map.*

Supplementary Figure 14

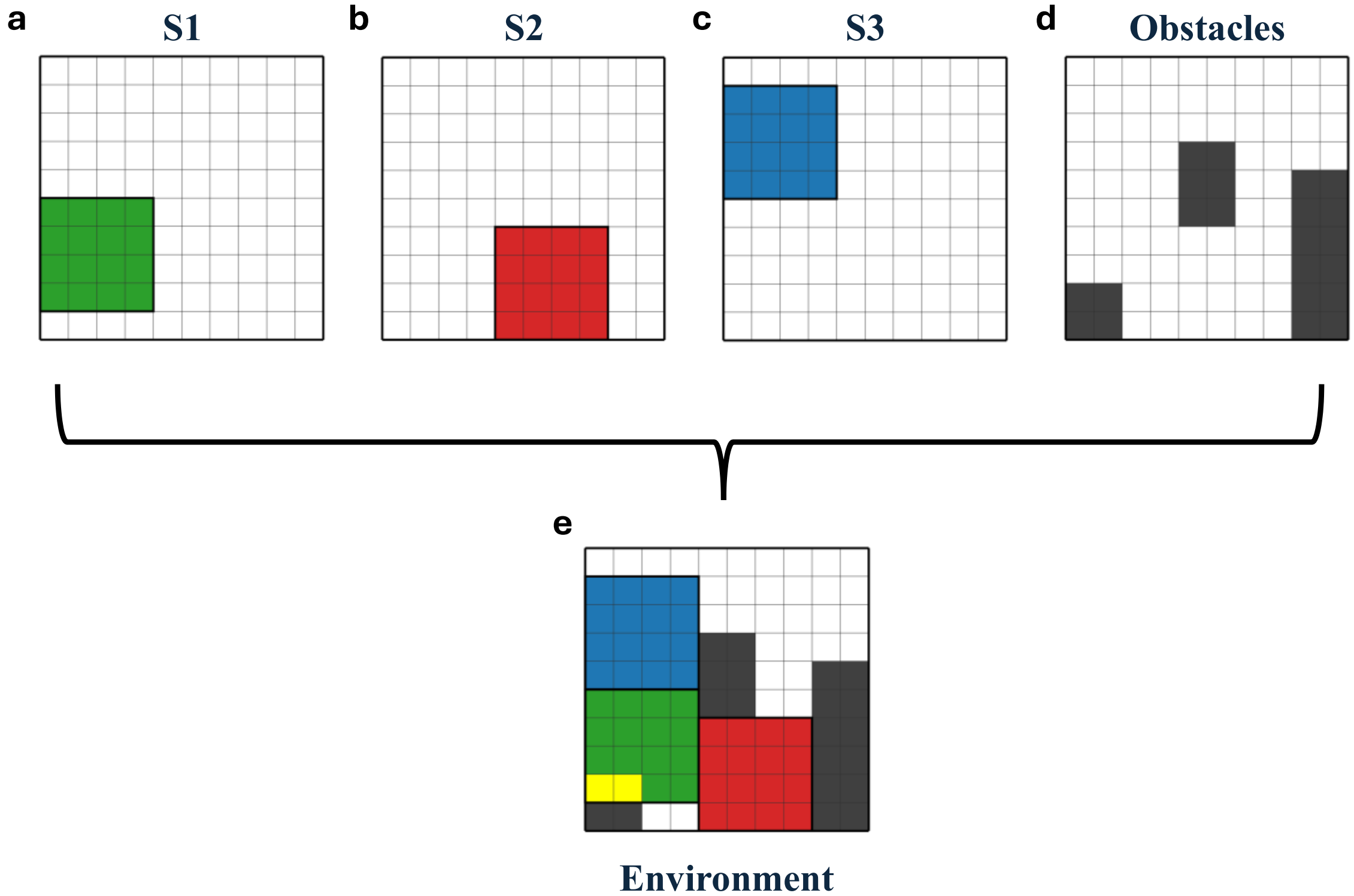

Supplementary ***Fig. 14. Calculation of Environment. (a–c)*** *Maps of the three stationary drones.* ***(d)*** *Obstacle map.* ***(e)*** *Environment map obtained by combining the stationary drone and obstacle maps.*

Supplementary Figure 15

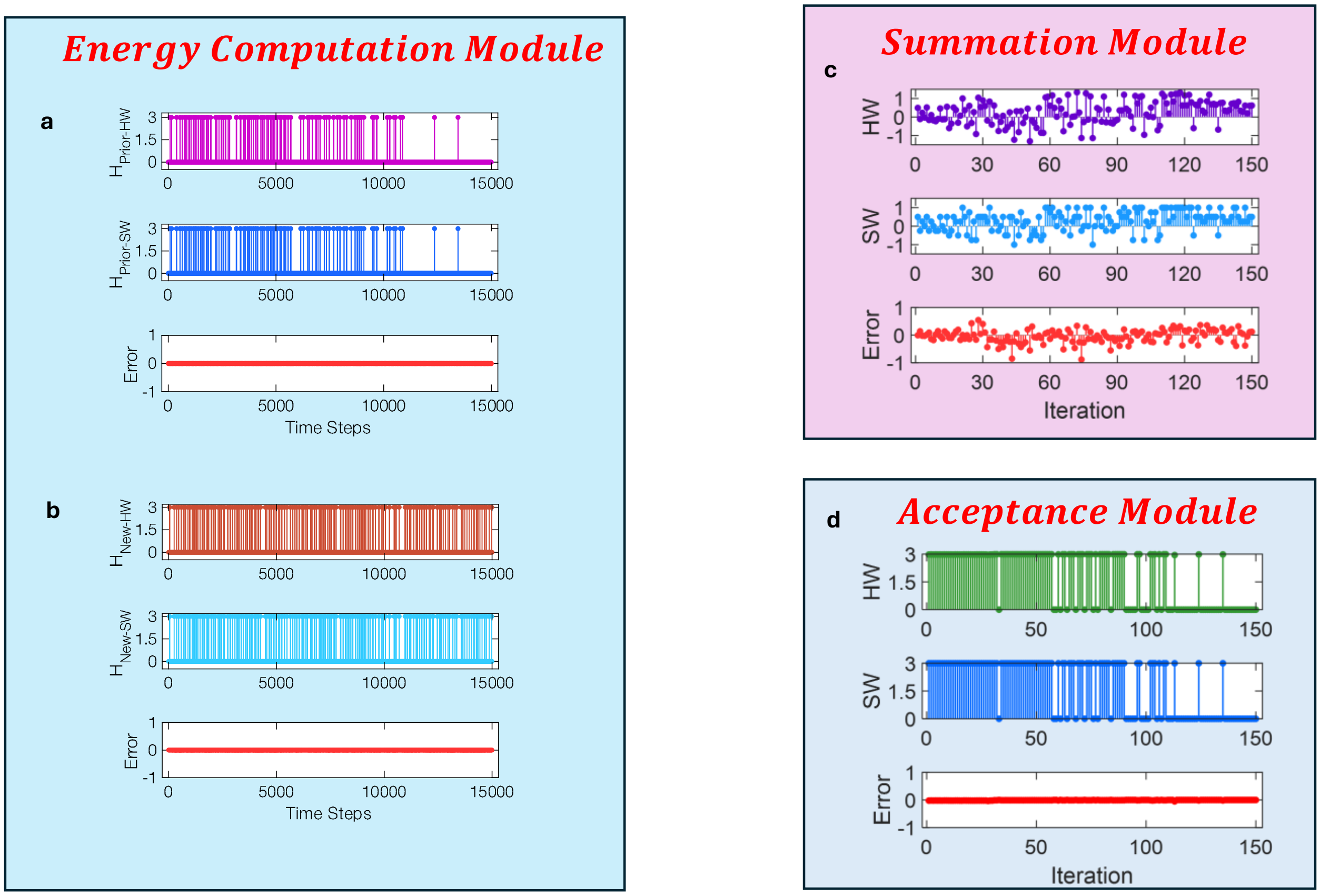

Supplementary ***Fig. 15. Characterization of experimental error.*** *Experimental and ideal values of* ***(a)*** H_{Prior} *and* ***(b)*** H_{New} *shown alongside the module errors for all 150 simulated annealing iterations. Experimental and ideal outputs of the* ***(c)*** *summation and* ***(d)*** *acceptance modules alongside the module errors for all 150 simulated annealing iterations.*

Supplementary Figure 16

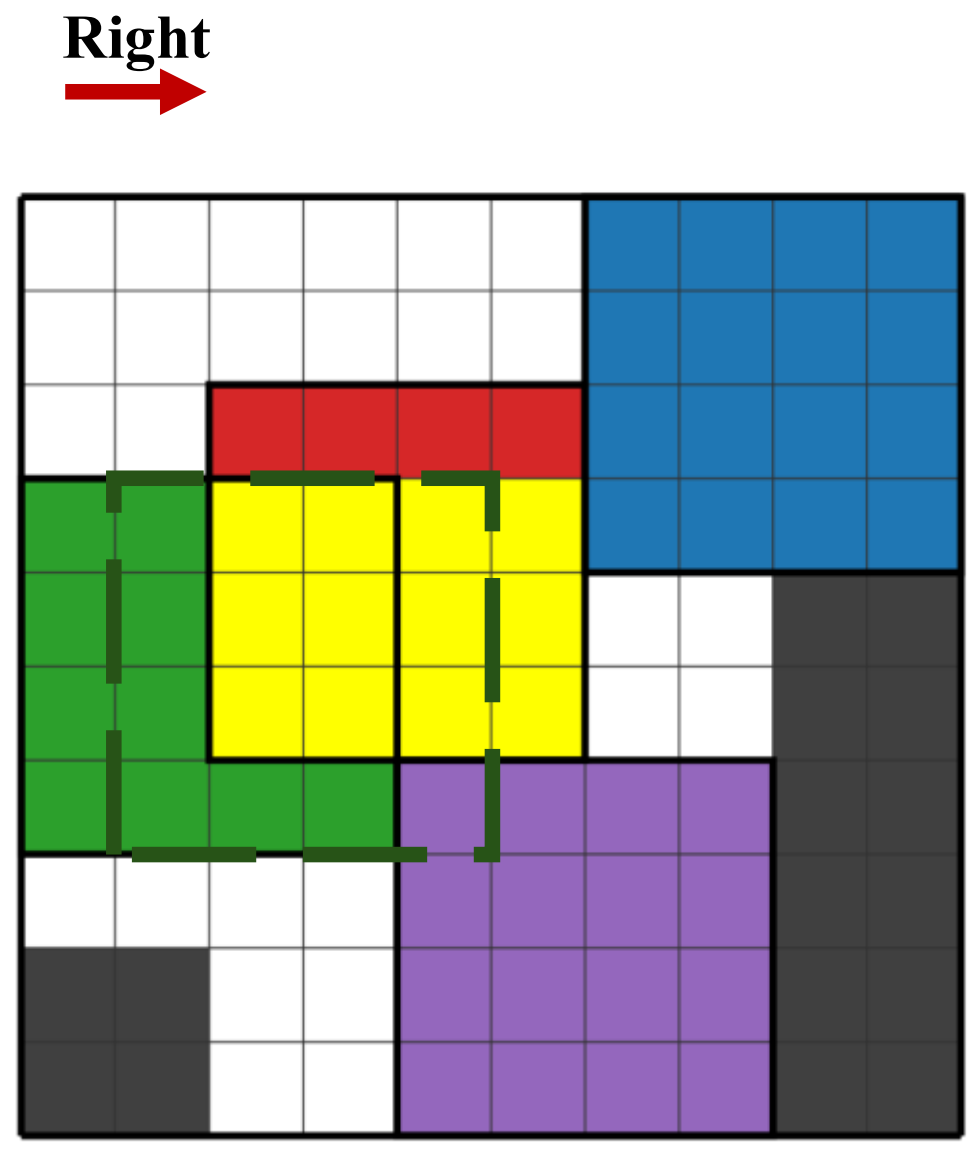

Supplementary ***Fig. 16. Proposed motion of green drone used for timing analysis.*** *The initial and proposed states used for evaluating the individual circuit timings which exhibit a prior overlap of 0 and new overlap of 4.*

Supplementary Figure 17

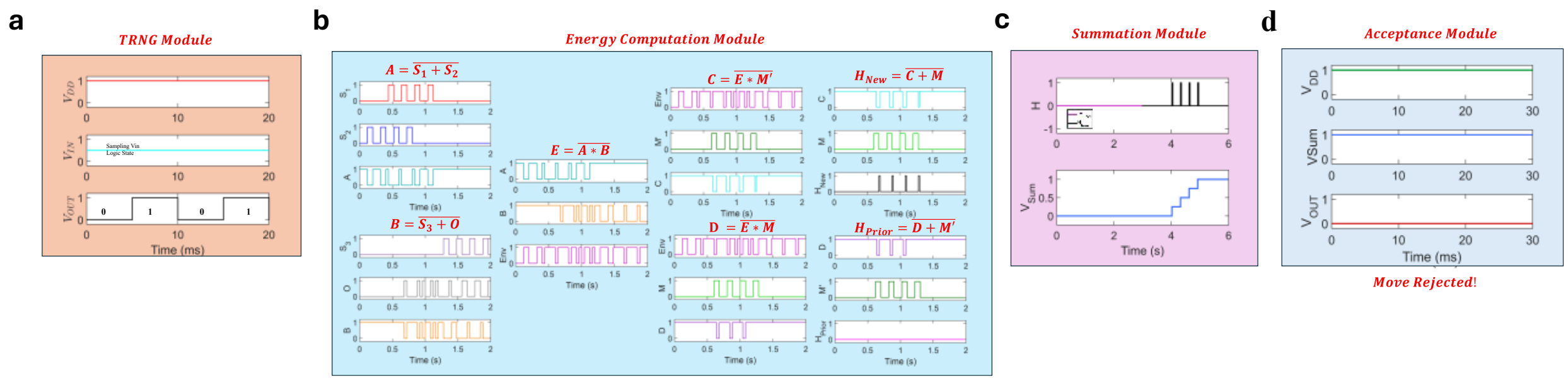

Supplementary ***Fig. 17. Timing response of all circuit modules used for SA.*** *Timing responses for one proposed rightward movement of the green drone for the* ***(a)*** *TRNG module sampled at 5 ms,* ***(b)*** *energy computation module sampled at 20 ms,* ***(c)*** *summation module sampled at 30 ms, and the* ***(d)*** *acceptance module sampled at 30 ms.*

Supplementary Figure 18

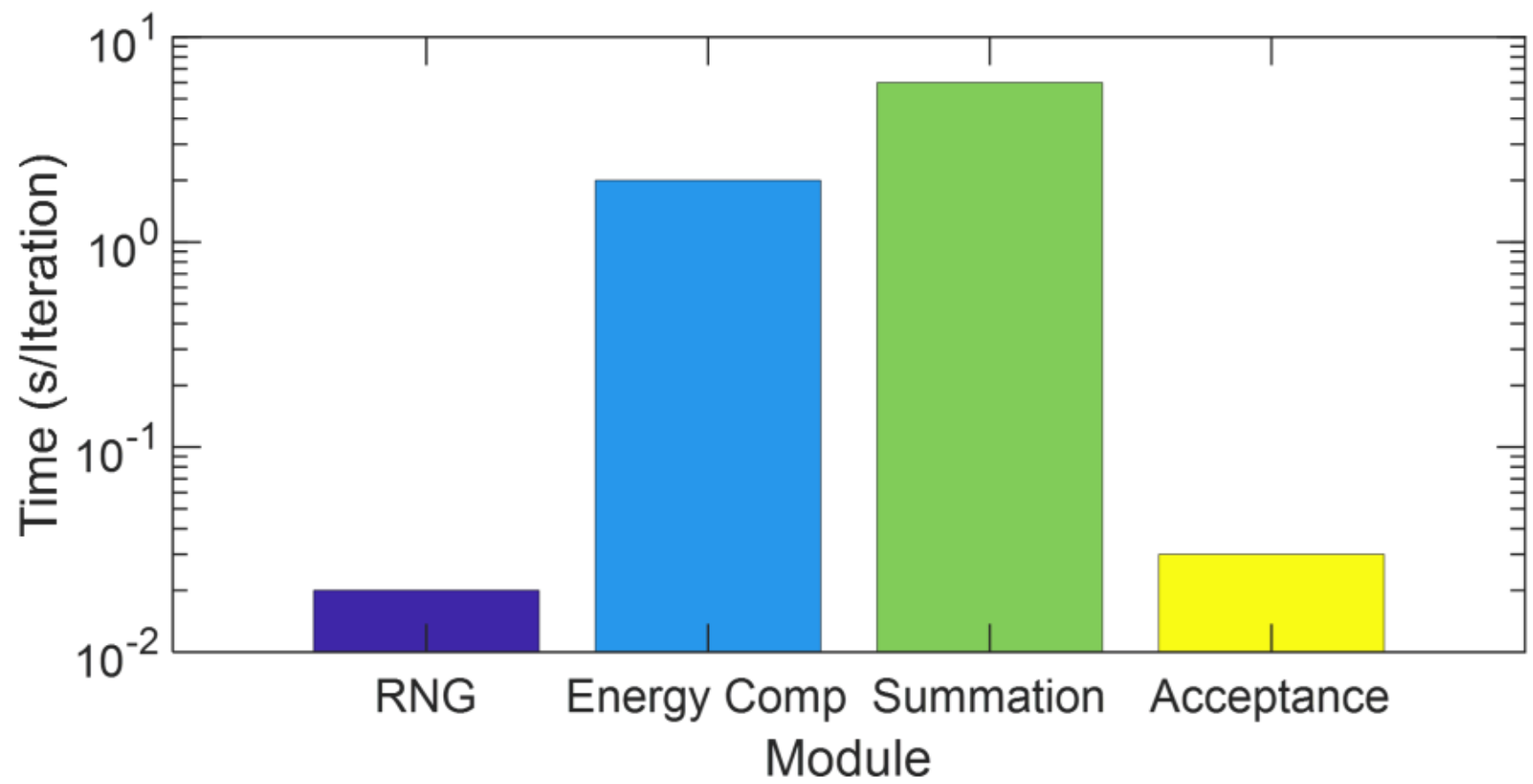

Supplementary *Fig. 18. Timing comparison of circuit modules used for SA.* *Bar plot of total time taken per module for one iteration of the simulated annealing algorithm.*

Supplementary Figure 19

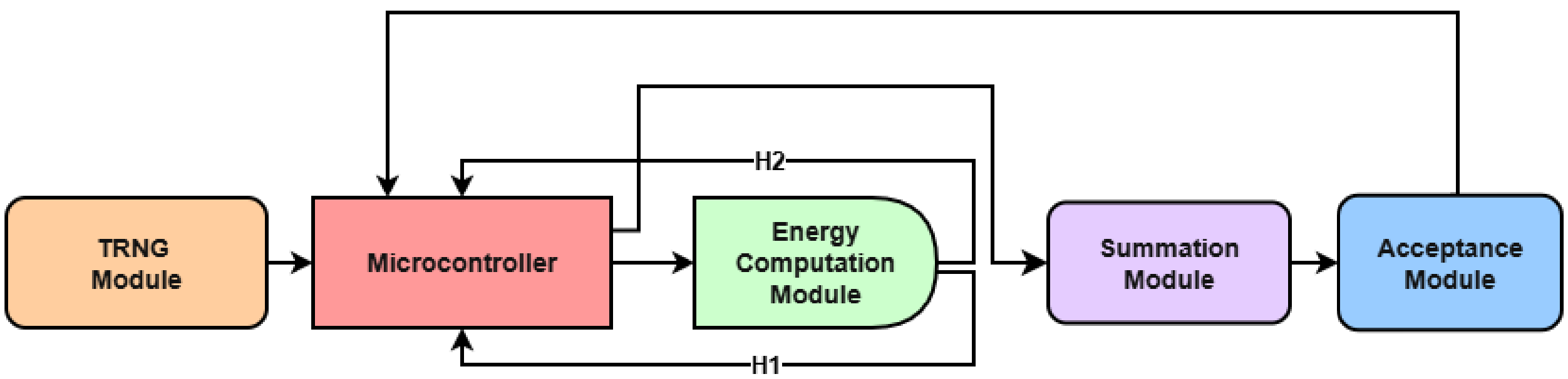

Supplementary ***Fig. 19. Interaction of SA Accelerator modules with peripheral circuitry.*** *Block diagram of the SA accelerator core modules together with the peripheral microcontroller that provides memory and system-level control.*

Supplementary Figure 20

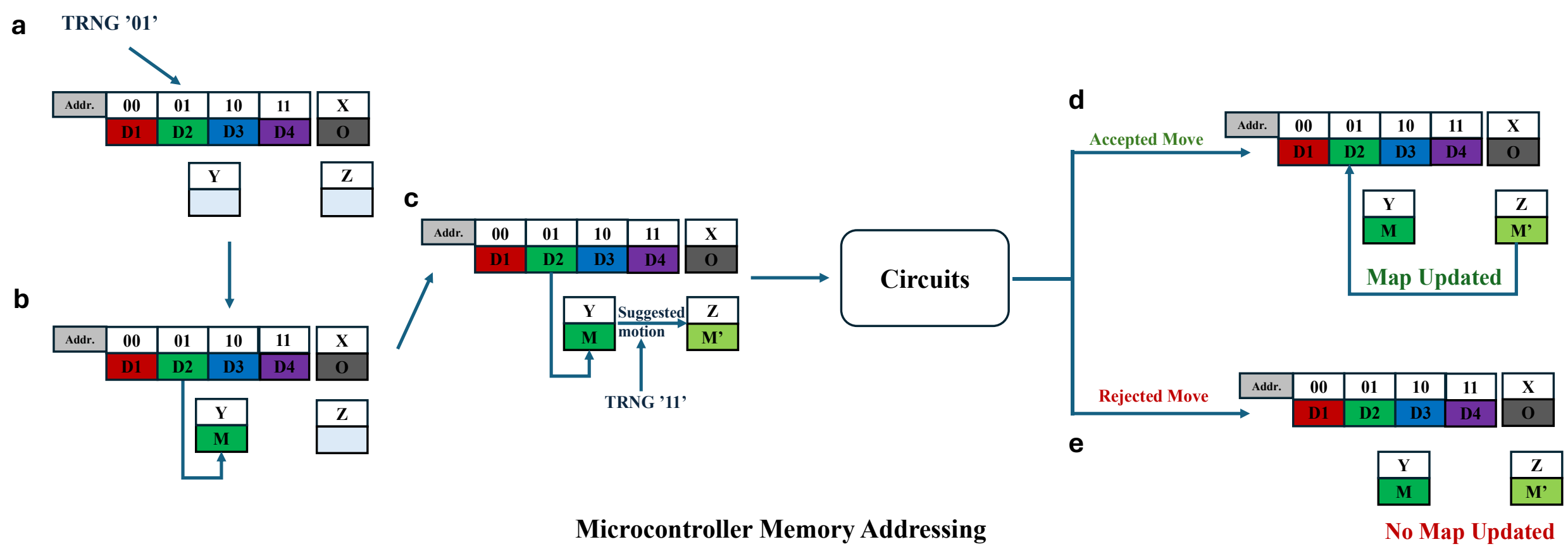

Supplementary *Fig. 20. Microcontroller Memory Addressing.* *Memory configuration of the microcontroller given the **(a)** initial state of the system comprising 4 drones and 1 obstacle map. **(b)** Microcontroller memory operation where a map at address 01 corresponding to D2 is designated as the active map, M, and a copy is written into the next available memory location, Y. **(c)** Storage of M' in memory location Z. Microcontroller memory operation **(d)** if the proposed move is accepted and **(e)** if the proposed move is rejected.*

Supplementary Figure 21

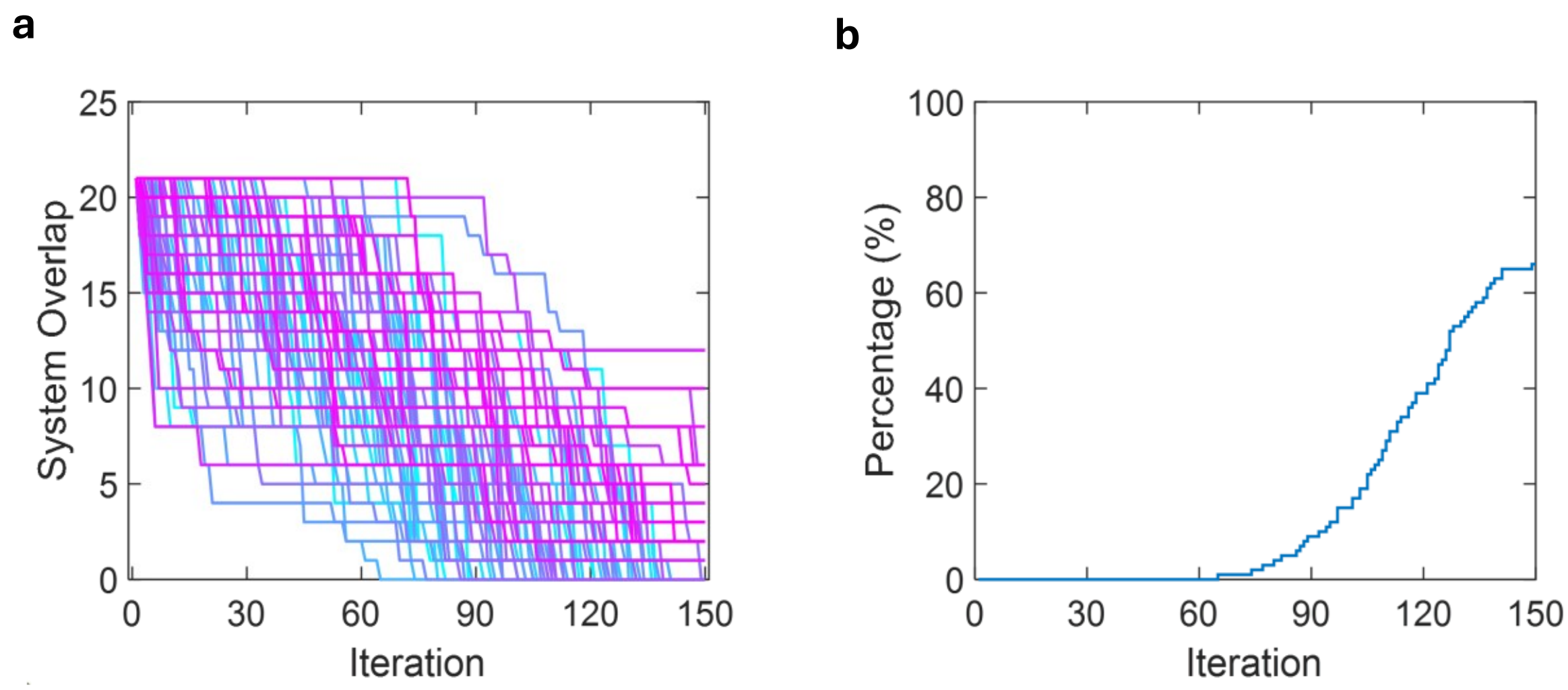

Supplementary ***Fig. 21. 100 initializations of experimental drone system. (a)*** *System overlap as a function of iteration for 100 SA initializations.* ***(b)*** *Cumulative distribution plot illustrating the percentage of converged initializations at each iteration.*

Supplementary Figure 22

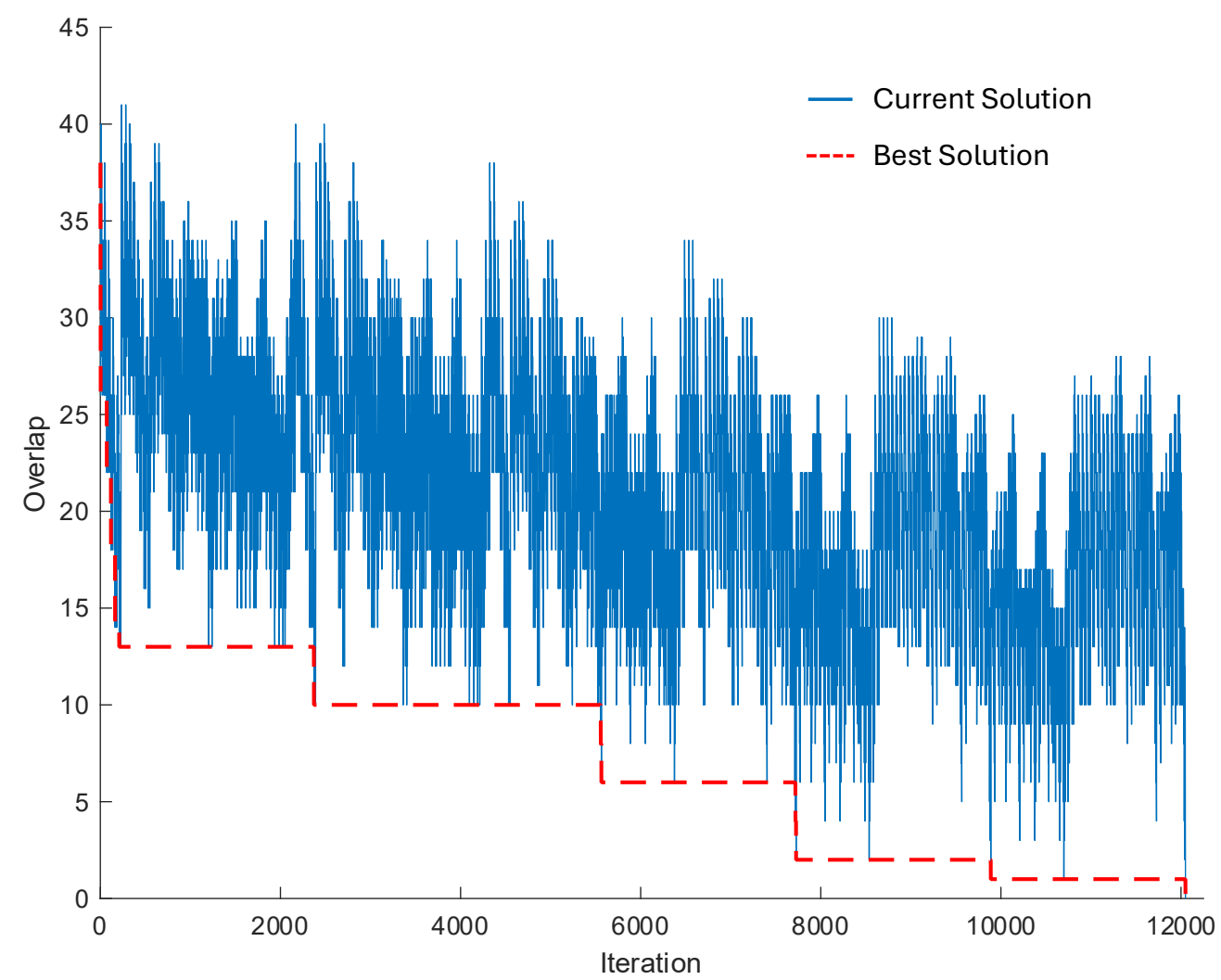

Supplementary *Fig. 22. Current solution vs best solution for a brute force search.* *The current solution and the best solution as a function of brute force search trial number for the 10×10 system.*

Supplementary Figure 23

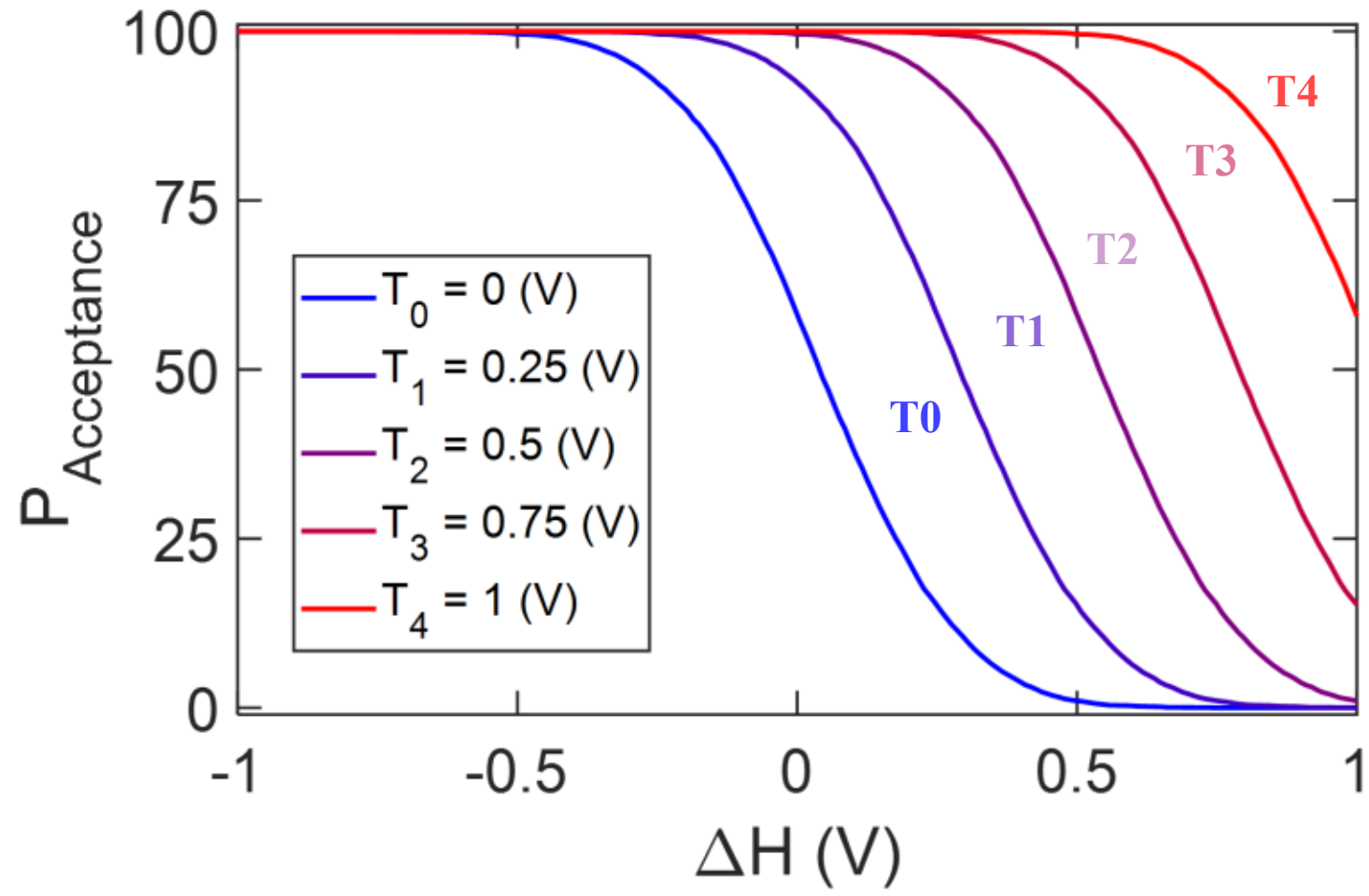

Supplementary ***Fig 23. Probability of solution acceptance with summation error.*** *The probability of acceptance as a function of summation output at several annealing temperatures with a* σ_{Sum} *of 0.2 V. System temperatures* T_0*,* T_1*,* T_2*,* T_3*, and* T_4 *correspond to acceptance module switching thresholds of 0 V, 0.25 V, 0.5 V, 0.75 V, and 1 V, respectively.*

Supplementary Figure 24

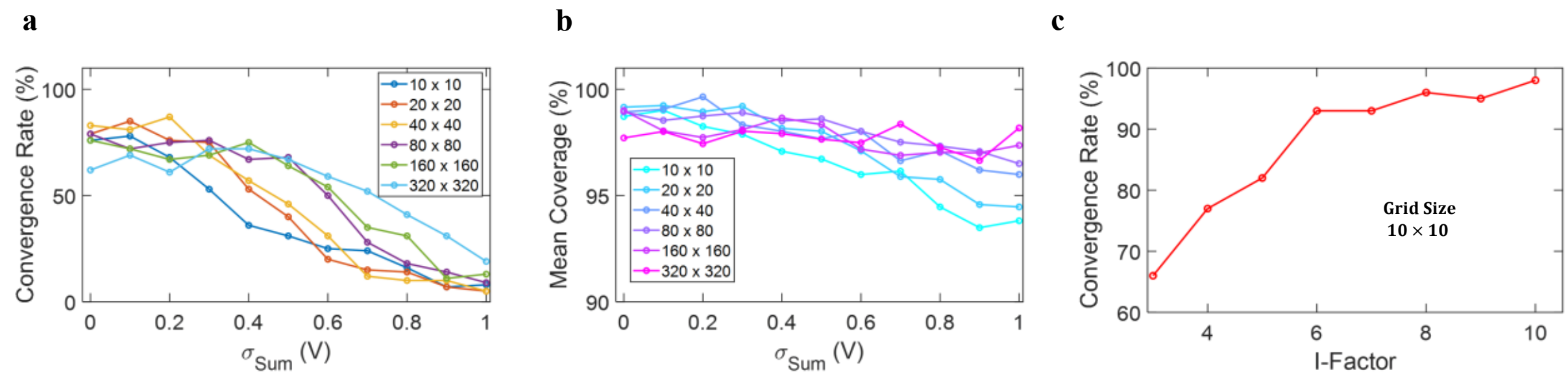

Supplementary ***Fig. 24. Effect of summation error on SA convergence. (a)*** *Convergence rate as a function of σ_{Sum} for 100 SA initializations at grid sizes of 10×10, 20×20, 40×40, 80×80, 160×160, and 320×320.* ***(b)*** *Mean coverage across 100 SA initializations as a function of σ_{Sum}. Coverage is above 92% for all system size and σ_{Sum} combinations.* ***(c)*** *Convergence rate as a function of the iterations per temperature factor (I) for 100 SA initializations of the 10×10 four drone, three obstacle system.*

Supplementary Figure 25

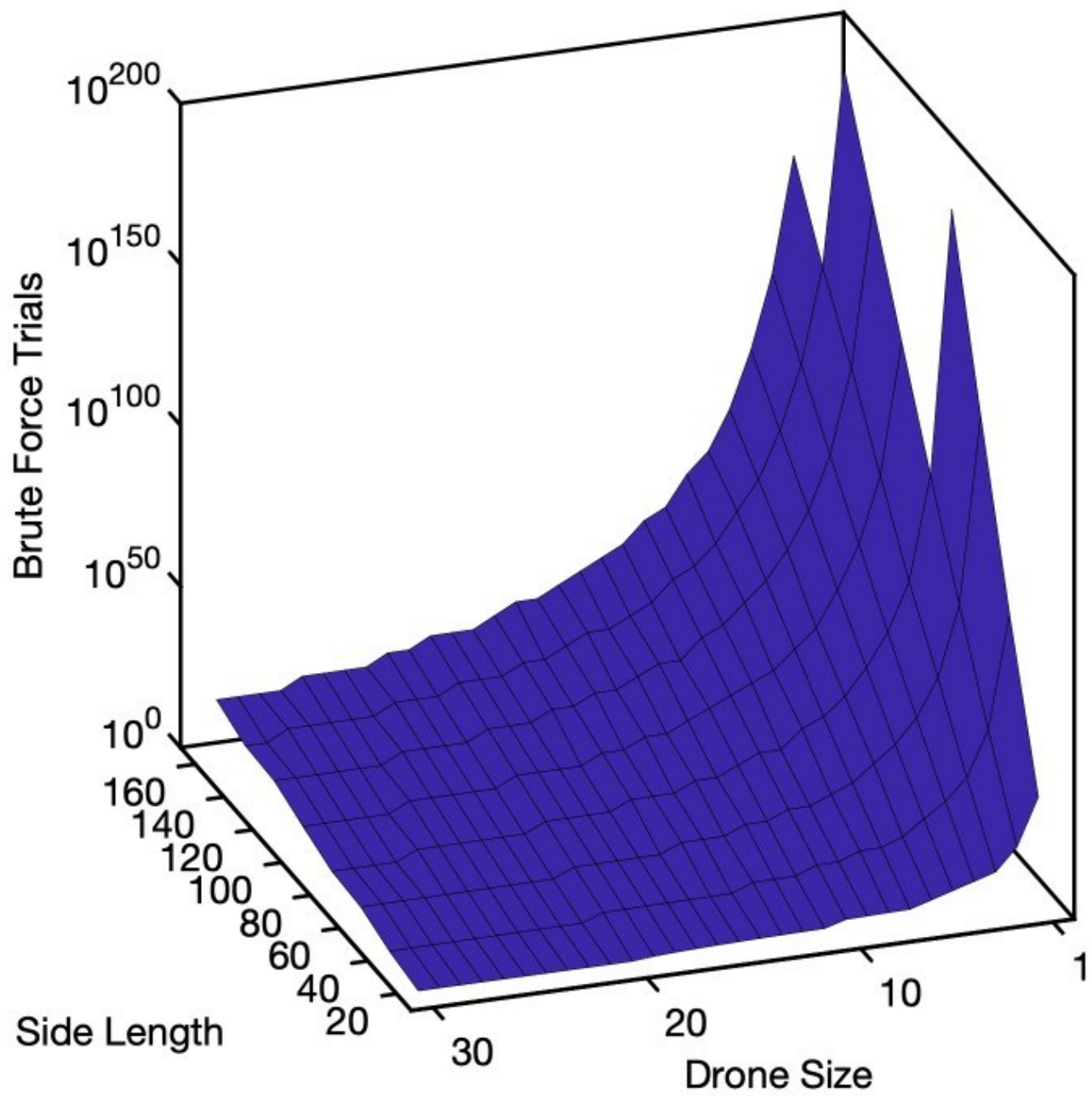

Supplementary ***Fig. 25. Problem complexity as a function of problem size.*** *The expected number of brute force trials required to find the optimal solution assuming a squared obstacle free grid at different drone sizes and lateral grid dimensions.*

Supplementary Table 1

Tech. / Param.	GPU [1]	FPGA [2]	CMOS [3]	RRAM & CBRAM [4]	2D Memtransistor (This Work)
Problem	Max Cut	QUBO	Ising Spin	Spin Glass	Spatial Optimization
I_{max} [mA]	N.A.	N.A.	6	0.2	0.01
I_{min} [mA]	N.A.	N.A.	N.A.	1e-6	10e-12
Operating Range [V]	N.A.	(0, 0.85)	(0, 0.1)	(-2, 2)	(-3, 3)
BEOL Integration	No	No	No	Yes	Yes
Vertical Integration	No	No	No	Yes	Yes
Radiation Hardness	No	Medium	No	High	High
Scalability	Limited	Low	Very Low	High	Very High
Drawback	Cost, Power	Limited Scalability	Limited Scalability	Process Complexity, IR-drop	Technology Maturity

Supplementary *Table 1. Benchmarking of SA hardware accelerator technologies.* *Benchmarking of SA hardware accelerator technologies that solve various combinatorial problems.*

Supplementary Table 2

NIST SP 800-22		
Statistical test	Sequence 1	
	p-value	Result
Frequency monobit	0.321	PASS
Block frequency	0.579	PASS
Cumulative sums (forward)	0.526	PASS
Cumulative sums (backward)	0.397	PASS
Runs	0.626	PASS
Longest run of ones	0.412	PASS
Rank	0.349	PASS
FFT	0.103	PASS
Non-overlapping Template	0.511	PASS
Overlapping Template	0.116	PASS
Approximate entropy	0.519	PASS
Universal	0.107	PASS
Random excursion	0.469	PASS
Random excursion variant	0.550	PASS
Serial	0.314	PASS
Linear complexity	0.273	PASS

Supplementary ***Table 2. NIST SP 800-22.*** *NIST SP 800-22 comprises 16 tests that were used to evaluate a set of one million randomly generated bits. All tests result in a p-value greater than 0.01 which confirms the true randomness of the evaluated bits.*

Supplementary Table 3

NIST SP 800-90B	
Non-IID Test	Entropy Rate Sequence 1
Most common value	0.9949
Collision	0.9248
Markov	0.9979
Compression	0.8615
t-Tuple	0.9381
Longest repeated substring	0.9921
Multi most common	0.9943
Lag prediction	0.9951
Multi MMC prediction	0.9977
LZ78Y prediction	0.9988
Minimum Entropy	***0.8615***

Supplementary ***Table 3. NIST SP 800-90B.*** *NIST SP 800-90B test used for characterization of the entropy source.*

References

[1] C. Cook, H. Zhao, T. Sato, M. Hiromoto, and S. X.-D. Tan, "GPU-based ising computing for solving max-cut combinatorial optimization problems," *Integration,* vol. 69, pp. 335–344, 2019.

[2] H. Kagawa *et al.*, "Fully-pipelined architecture for simulated annealing-based QUBO solver on the FPGA," in *2020 Eighth International Symposium on Computing and Networking (CANDAR)*, 2020: IEEE, pp. 39–48.

[3] M. Yamaoka, C. Yoshimura, M. Hayashi, T. Okuyama, H. Aoki, and H. Mizuno, "24.3 20k-spin Ising chip for combinational optimization problem with CMOS annealing," in *2015 IEEE International Solid-State Circuits Conference-(ISSCC) Digest of Technical Papers*, 2015: IEEE, pp. 1–3.

[4] J. H. Shin, Y. J. Jeong, M. A. Zidan, Q. Wang, and W. D. Lu, "Hardware acceleration of simulated annealing of spin glass by RRAM crossbar array," in *2018 IEEE International Electron Devices Meeting (IEDM)*, 2018: IEEE, pp. 3.3. 1–3.3. 4.
