# Supplementary material for: Hardware acceleration of simulated annealing for constraint satisfaction problems
Source: Nat Commun. 2026 Jul 21;17:8905. doi: 10.1038/s41467-026-74893-1 (PMC13503799; doi:10.1038/s41467-026-74893-1)
Supplement: Supplementary file 2 [file 41467_2026_74893_MOESM5_ESM.pdf]

nature portfolio

Peer Review File

Hardware Acceleration of Simulated Annealing for Constraint Satisfaction Problems

Corresponding Author: Professor Saptarshi Das

This file contains all reviewer reports in order by version, followed by all author rebuttals in order by version.

Version 1:

Reviewer comments:

Reviewer #1

(Remarks to the Author)
This work presents an effective approach to implementing hardware simulated annealing using 2D CMOS circuits. It provides experimental results on 2D transistors and circuits. This work also introduces hardware acceleration on spatial optimization tasks. However, the organization of this paper requires improvement, and more explanations of the system are needed. My detailed comments are listed as follows:

1. Please add more explanations of the problem formulation and obstacle representation. Is there a dataset available for this problem, and are there any other works that solve the same problem? If so, please also compare this work with former works.

2. Please clarify the contribution of this work. What is the main advantage of this work, compared with brute-force searching? Does the advantage mainly come from the algorithm or the circuit implementation? Can approaches based on the conventional CMOS circuits or other memtransistors achieve the same advantages?

3. The circuits proposed in this work might require peripheral circuits to perform simulated annealing computation. How do the 2D CMOS circuits interact with off-chip peripheral circuits and the controlling modules?

4. This work presents the TRNG results in Figure 2. Could the TRNG module in this work pass the NIST test? How do these characteristics influence the simulated annealing results?

5. Please illustrate the bandwidth or generating speed of the TRNG module, since it could influence the computing speed of the system. It would also help if the authors could characterize the entropy source of this work.

6. It requires a timing diagram that shows how TRNG and other circuit components are incorporated in the system. The bitstreams shown in Figure 3 might be confusing to the readers. It would be better if there were more explanations.

7. Please also illustrate whether this work can generalize to other constraint satisfaction problems.

8. The authors show the evolution process in this work regarding the iterations or time steps. The readers may also be concerned about the real-time computing speed of the proposed circuits. It would be helpful if the authors could provide more characteristics of the proposed transistors and circuits on timing response.

Reviewer #2

(Remarks to the Author)
This work demonstrates a hardware accelerator for simulated annealing (SA) using 2D-material-based CMOS circuits. It demonstrates spatial optimization for drone placement with obstacles problems efficiently. The system achieves ~1800x speedup over brute-force search with low energy consumption, leveraging stochasticity and programmability of 2D devices. However, there are still several critical comments that need to be addressed:

1) The key drawback of this work is that the implementation of SA for constraint satisfaction problems appears to be done by

pure simulation without experimental verifications. It is unclear to what extent the actual device behaviors (including the non-ideal characteristics) and peripheral circuits are taken into consideration in the system-level benchmark.

2) The Introduction mentions prior acceleration efforts using GPUs, FPGAs, and RRAM/CBRAM devices but remains largely qualitative. A quantitative comparison table summarizing key metrics (e.g., energy efficiency, processing speed, area efficiency) across these platforms would provide valuable context and highlight the advantages of the proposed approach more clearly.

3) The manuscript highlights the performance advantage of the hardware accelerator over an exhaustive brute-force search. However, a comparison with a software-based SA implementation running on a general-purpose processor would be highly informative. It is recommended to supplement the analysis, for instance by adding a cumulative distribution curve for a software SA baseline in Figure 5f, comparing metrics like the median number of iterations to convergence and failure rates on identical problem instances.

4) Fig. 5f shows that a small subset of initializations failed to reach the global optimum. An analysis of the underlying reasons for these failures would be insightful. Furthermore, beyond the TRNG, it would be valuable to discuss whether non-idealities in the 2D devices (e.g., variability, fluctuations) may influence convergence behavior, especially as the system scales in the number of drones or grid size. Do these hardware characteristics help escape local minima or impede convergence?

5) The acceptance module relies on programmable memtransistors whose retention was shown for 200 seconds (Extended Data Fig. 8). Since the annealing process involves 5 times temperature changes and 50 on-chip experiments, it is important to evaluate endurance through repeated programming cycles (e.g., >250 cycles) and measure retention properties after endurance testing.

6) The current work focuses on a specific coverage-overlap model with an energy functon(H) implemented via relatively simple logic operations. The authors should comment in the Conclusion or Discussion on the generalizability of this architecture to other optimization problems (e.g., TSP), including any necessary modifications at the hardware or algorithm level.

7) There are some labeling issues in the figures. For example, the y-axis in Fig. 2c is missing partial labels (numbers 2 and 3), and the y-axis label in Fig. 5d should be "Number of Drones".

Reviewer #3

(Remarks to the Author)
The title of this paper initially led me to believe that the authors had developed a complete system to solve the constraint satisfaction problem using quantum annealing. However, it appears that they merely simulated a potential hardware implementation without combining a few basic circuits they report into a system. The simulations are relatively straightforward, and the experimental aspect feels incomplete. Consequently, I do not think this work is ready for publication in Nature Communications. So, my recommendation is to reject.

Version 2:

Reviewer comments:

Reviewer #1

(Remarks to the Author)
The authors have significantly expanded the results and have made substantial improvements to the manuscript overall. Most of the responses seem to be satisfactory, while I still remain one concern:

In the response to comment 5, the authors indicate that the TRNG speed is 200 Hz. This suggests that the random number generation rate of the 2D memtransistors in this work could become a bottleneck for hardware acceleration.
Existing works have shown that RRAM could be integrated in monolithic 3D technology and this technology supports certain extent of logic capabilities. Moreover, RRAM-based TRNGs have been reported to achieve bandwidths at the Mbps level. Therefore, it is important for the authors to clarify the reason of choosing 2D memtrasistors over RRAM, in light of the 3D integration capability and the fast TRNG of RRAM.
It is understandable that 2D memtransistor technology is still at an early stage. It would be valuable if the authors could discuss the operating speed comparison on RRAM and this work. An explanation on the potential advantages of 2D memtransistors would also be helpful.

Reviewer #2

(Remarks to the Author)
I appreciate the efforts that the authors have made to address the previous comments, and the revisions have improved the manuscript. However, there are still several critical concerns that need to be addressed:

1) The authors should explicitly specify the exact locations where revisions have been incorporated in the revised manuscript. Vague statements such as "The above comparisons have been added to the revised manuscript" are insufficient, as they place an undue burden on reviewers to find the changes.

2) While the authors provide images of individual circuit components (e.g., TRNG and inverters), there is a lack of experimental evidence for the integrated system. Specifically, a physical photograph of the complete annealing accelerator (Fig. 1a) is essential to validate the hardware implementation. This should include visible interconnections between modules (e.g., TRNG, energy computation, summation, and acceptance units) to demonstrate practical integration and scalability.

3) The term "hardware-realistic simulation" is used ambiguously throughout the manuscript. The authors must clearly delineate which results are derived from experimental measurements and which are based on simulations. Explicit evidence should be provided.

4) The claimed 1800-fold acceleration over brute-force search requires clearer context. The authors should clarify whether this improvement stems primarily from the simulated annealing algorithm itself or the hardware implementation. If the acceleration is algorithmic, the novelty of the hardware implementation must be emphasized without overstating its role.

5) The median convergence iteration count for software-based SA (113.5) differs from the hardware-reported value. The authors should explain this discrepancy.

6) Although hardware errors are shown to be negligible in small-scale experiments, their cumulative effect in larger systems (e.g., higher-dimensional grids or more drones) must be evaluated. The authors should provide simulations or theoretical estimates of how error propagation could affect convergence reliability, along with potential mitigation strategies.

Version 3:

Reviewer comments:

Reviewer #1

(Remarks to the Author)
I thank the authors for their effort in revising the manuscript and addressing previous concerns. My questions have been addressed. The revised manuscript is in good shape and I would like to recommend it for publication.

Reviewer #2

(Remarks to the Author)
I have carefully reviewed the authors' rebuttal and the revised manuscript. The authors have addressed some of the previous concerns, particularly regarding error analysis and terminology clarification. However, several critical issues related to the hardware implementation and its characterization remain unresolved.

1) The authors have provided an image of the two chips in the probe station (Fig. R2). However, the manuscript still lacks detailed optical images of the key functional modules (Energy Computation, Summation, and Acceptance modules) as physically implemented on these chips. Currently, only the TRNG module and isolated logic gates (NAND, NOR and Inverter) are shown. More importantly, the authors confirm that "all measurements were performed modularly" with external components. This acknowledges that a fully integrated, single-chip, end-to-end hardware demonstration was not achieved. I would recommend that the revised manuscript should at least provide images of the actual Energy Computation and Acceptance circuit blocks as fabricated. Furthermore, to avoid any misleading, the manuscript should explicitly describe the modular hardware implementation and discuss the limitation of such modular demonstration.

2) The reported energy consumption of ~76 nJ per iteration is a key metric. However, it is still unclear if this final figure includes all relevant energy overheads, such as these from the external microcontroller (Extended Data Fig. 18).

3) The authors note a discrepancy between the hardware convergence iteration (134) and the median software simulation convergence (113.5), attributing it to algorithmic stochasticity. To perform a more controlled and insightful comparison, I would recommend the authors run their software simulations using the exact sequence of random bits generated and used by the physical TRNG module during the hardware experiment.

4) While the authors state they have replaced "hardware-realistic simulation" with "simulation of the annealing accelerator", I note that the term "hardware-realistic" still appears in the revised manuscript (e.g., Page 27 Figure title: " Hardware-realistic simulation of SA algorithm."). The authors should ensure consistent terminology throughout the manuscript, replacing all instances with the clarified description.

Version 4:

Reviewer comments:

Reviewer #2

(Remarks to the Author)
The authors have addressed the remaining comments in the revision and I believe this work can now be accepted for publication.

Open Access This Peer Review File is licensed under a Creative Commons Attribution 4.0 International License, which permits use, sharing, adaptation, distribution and reproduction in any medium or format, as long as you give appropriate credit to the original author(s) and the source, provide a link to the Creative Commons license, and indicate if changes were made.
In cases where reviewers are anonymous, credit should be given to 'Anonymous Referee' and the source.
The images or other third party material in this Peer Review File are included in the article's Creative Commons license, unless indicated otherwise in a credit line to the material. If material is not included in the article's Creative Commons license and your intended use is not permitted by statutory regulation or exceeds the permitted use, you will need to obtain permission directly from the copyright holder.
To view a copy of this license, visit https://creativecommons.org/licenses/by/4.0/

Reviewer #1 (Remarks to the Author):

This work presents an effective approach to implementing hardware simulated annealing using 2D CMOS circuits. It provides experimental results on 2D transistors and circuits. This work also introduces hardware acceleration on spatial optimization tasks. However, the organization of this paper requires improvement, and more explanations of the system are needed. My detailed comments are listed as follows:

1. Please add more explanations of the problem formulation and obstacle representation. Is there a dataset available for this problem, and are there any other works that solve the same problem? If so, please also compare this work with former works

We thank the reviewer for their thorough and constructive comments. For ease of reference and discussion, we have separated this comment into two different sections.

1a. Please add more explanations of the problem formulation and obstacle representation.

The hardware-based simulated annealing (SA) accelerator proposed in our manuscript solves a constrained spatial optimization problem wherein a set of agents must be placed within an environment while adhering to several constraints. The goal is to generate a solution that achieves maximum system coverage while avoiding obstacle regions. System coverage is defined as the total area occupied by agents within the environment excluding regions designated as obstacles. This problem can be related to two-dimensional (2D) packing and placement tasks, but we chose to describe the problem formulation in the context of drone placement. In this case, a 10×10 grid is utilized as the discrete spatial domain occupied by drones and obstacles. The position of each drone is encoded into a binary occupancy map that describes its location within the system. Occupancy maps for four representative drones of size 4x4 are shown in **Fig. R1a-d**. The randomly initialized position of each drone is modeled as an independent map where cells corresponding to their occupied positions have a value of one while all other cells remain at zero. The occupancy map corresponding to a set of three randomly initialized obstacles is shown in **Fig. R1e**. Here, obstacles represent either physical structures within the environment that restrict drone movement or no-fly zones. Obstacles are randomly initialized across the grid and remain stationary throughout the optimization process effectively functioning as immobile drones. The initial state of the system with all drones and obstacles combined into the same occupancy map is shown in **Fig. R1f**. Overlap regions, highlighted in yellow, reflect redundant coverage either among agents or between agents and obstacles.

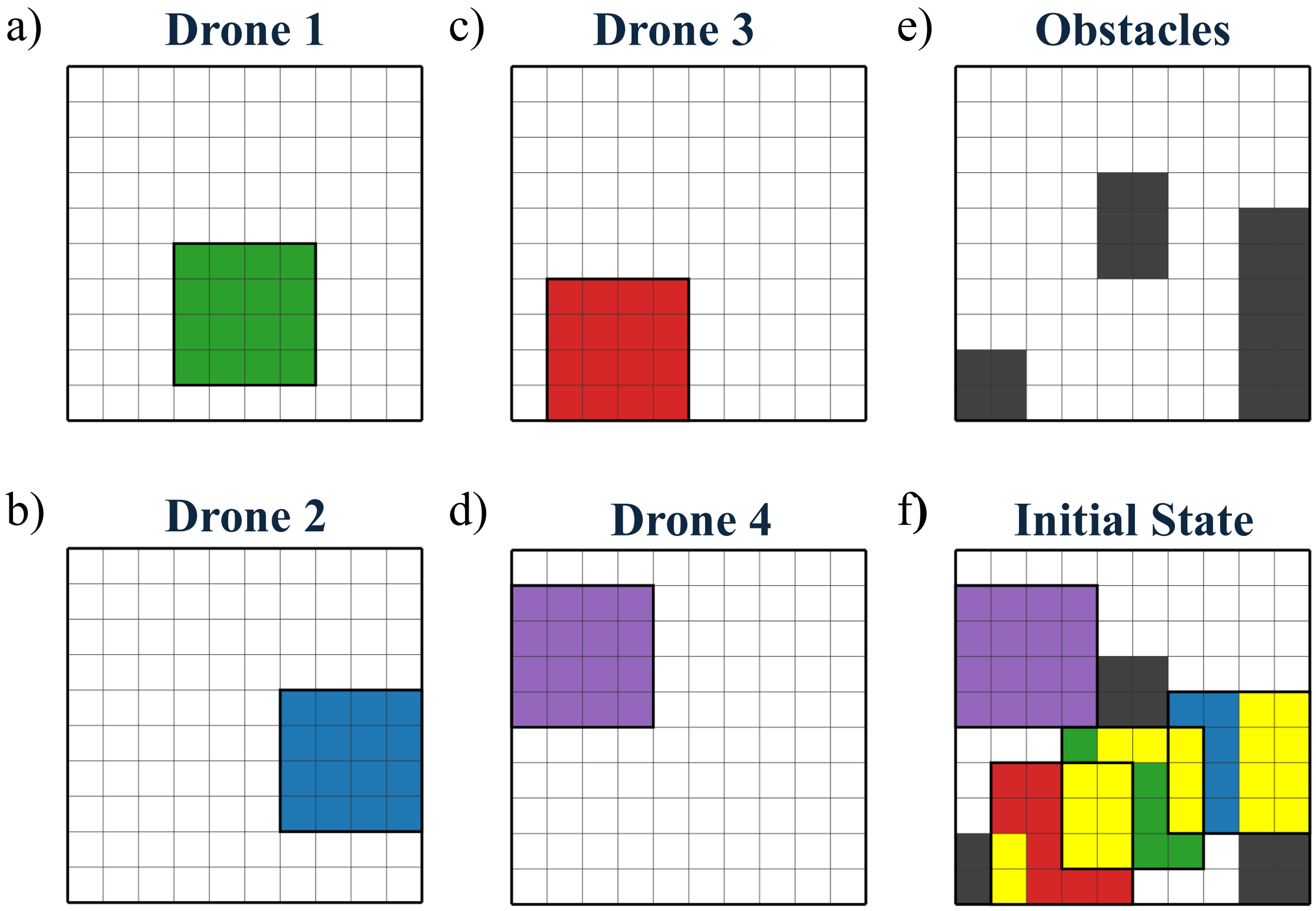

Fig. R1. Occupancy maps of Drones and obstacles. (a-d) *Occupancy maps of drones 1, 2, 3, and 4.* ***(e)*** *Occupancy map of three obstacles.* ***(f)*** *Initial state of the system with all drones and obstacles combined into the same occupancy map.*

Our SA accelerator aims to maximize coverage by minimizing the system overlap. During each iteration, a proposed solution is created by sampling the true random number generator (TRNG) module which produces a stochastic bitstream corresponding to the random selection of an agent and its proposed movement direction. In this example with four drones, two bits of the TRNG module bitstream determine the identity of the moving drone (00, 01, 10, or 11 for drone 1, drone 2, drone 3, or drone 4) while two additional bits determine the movement direction (00, 01, 10, or 11 for upward, downward, leftward, or rightward movement). To ensure valid drone movements, several constraints are imposed such that drones remain within the permissible bounds of the grid. Each drone's motion must adhere to these global restrictions, which are enforced by the microcontroller to prevent out-of-boundary behavior. The top-left corner of each 4×4 drone footprint serves as its anchor point. Suppose the anchor of a moving drone is located at position (R, C) on the 10×10 grid. When a movement is proposed, one of four actions occurs with equal probability:

$$\text{Up:} \qquad R' = R_o - 1, \quad C' = C_0$$

$$\text{Down:}\quad R' = R_o + 1, \quad C' = C_0$$

$$\text{Right:}\quad R' = R_o, \quad C' = C_0 + 1$$

$$\text{Left:}\quad R' = R_o, \quad C' = C_0 - 1$$

Where R_o, R', C_0, and C' correspond to the prior row of the moving drone anchor, the proposed row of the moving drone anchor, the prior column of the moving drone anchor, and the proposed column of the moving drone anchor, respectively. For each proposed drone movement, the new anchor coordinates must satisfy the following boundary conditions:

$$R' > 0 \tag{1}$$

$$C' > 0 \tag{2}$$

$$R' + L_D - 1 \leq 10 \tag{3}$$

$$C' + L_D - 1 \leq 10 \tag{4}$$

Where L_D corresponds to the drone's side length (4 in this case). These constraints ensure that the entire 4×4 drone always remains within the limits of the 10×10 grid. If this constraint is satisfied, the overlap computation is carried out at the circuit level. **Fig. R2a** shows a map of the initial occupancy of the moving drone, M, and the proposed occupancy of the moving drone, M'. The solid blue box is representative of the initial drone position, and the dashed blue line indicates the proposed drone position chosen based on the output of the TRNG module. The occupancies of all stationary drones and obstacles are compressed together with the environment calculation submodule as seen in **Equation R1** below:

$$E = S_1 + S_2 + S_3 + O \tag{R1}$$

Here, E is the environment seen by the moving drone, S_1 is the occupancy of the first stationary drone, S_2 is the occupancy of the second stationary drone, S_3 is the occupancy of the third stationary drone, and O is the occupancy of the system obstacles.

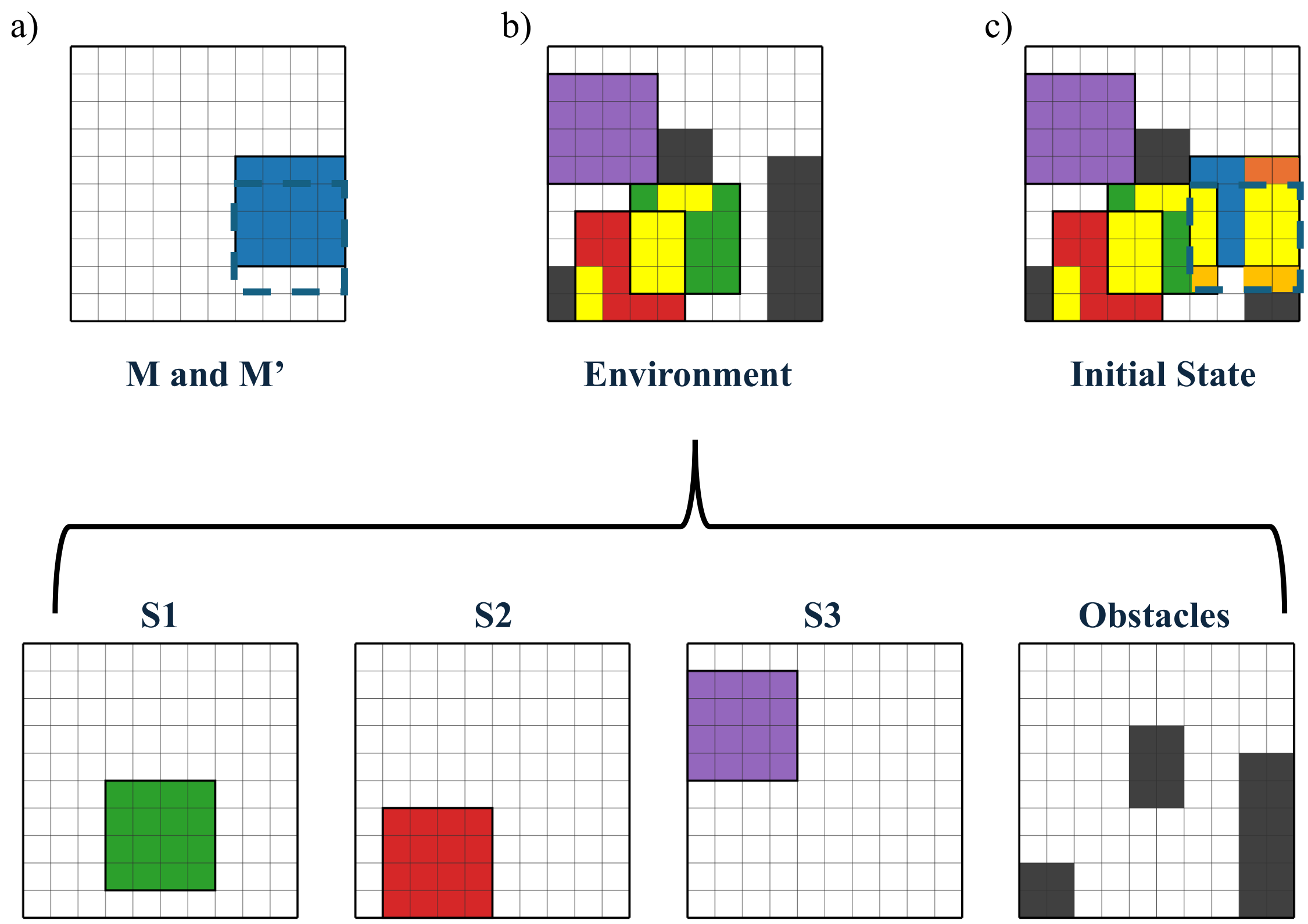

Fig. R2. Calculation of Environment. (a) *Map of the initial occupancy of the moving drone, M, and the proposed occupancy of the moving drone, M'. The solid blue box is representative of M, and the dashed blue line indicates M'.* ***(b)*** *Occupancy map of E and the individual occupancy maps of $S1$, $S2$, $S3$, and O that contribute to E.* ***(c)*** *initial state of the system combining M, M', and E. Here, overlap regions corresponding to H_{Prior} and H_{New} are shown in orange and gold, respectively.*

Fig. R2b shows a map of E and the individual occupancy maps that contribute to E. Subsequently, the overlap calculation submodules are used to quantify the system overlap unique to the prior solution, H_{Prior}, and the system overlap unique to the proposed solution, H_{New}, as seen in **Equation R2** and **Equation R3** below:

$$H_{Prior} = E * M * \overline{M'} \tag{R2}$$

$$H_{New} = E * \overline{M} * M' \tag{R3}$$

Within these equations, the terms $M * \overline{M'}$ and $\overline{M} * M'$ isolate the occupancy that is unique to the drone in the prior solution and the new solution, respectively, while multiplication with E identifies overlapping regions. **Fig. R2c** depicts the initial occupancy of the moving drone, the proposed occupancy of the moving drone given a random downward movement, and the environment seen by the moving drone. Here, overlap regions corresponding to H_{Prior} and H_{New} are shown in orange and gold, respectively. Overlap that is not unique to either H_{Prior} or H_{New} is shown in yellow.

Next, H_{Prior} and H_{New} are given as inputs to the summation module that computes **Equation R4** listed below:

$$\Delta H = H_{New} - H_{Prior} \qquad \text{(R4)}$$

This module sums the entire bitstream, compressing all information into a single-valued ΔH that is fed into the acceptance module. As seen, downward movement increases the total overlap of the system by 1, yielding a small positive ΔH. Depending on the temperature prescribed by the annealing schedule, downward movement may be accepted, allowing the algorithm to explore the solution space and potentially escape a local minimum. If the computed energy lies within the allowable threshold, the move is accepted, and the updated drone and obstacle maps are written back to memory. Conversely, if the threshold condition is violated, the move is rejected, and the previous state is retained. This process enables real-time, hardware-driven decision making, ensuring that all drone movements adhere to the defined physical and spatial constraints while optimizing energy efficiency through circuit-level computation. A more detailed explanation of the problem statement has been added to the revised manuscript.

1b. Is there a dataset available for this problem, and are there any other works that solve the same problem? If so, please also compare this work with former works

The constrained spatial optimization problem that we address can be broadly represented as a 2D packing and placement task. The formulation of this problem is similar in structure to very-large-scale integration (VLSI) floorplan design tasks where fixed-footprint objects must be positioned on a grid without overlap while respecting forbidden regions. Constraint satisfaction problems such as graph coloring, max-cut, eight queens, sudoku or the spatial optimization task described here do not generally have a fixed dataset for comparison. Rather, the problem is defined by the nature of the variables, system, and given constraints. For example, a sudoku board can vary in size, quantity of clues, and position of clues.

As discussed in our response to comment 2, the main contribution of our work is the hardware acceleration of the simulated annealing algorithm using complementary metal-oxide-semiconductor (CMOS) circuits based on 2D semiconductor materials. Therefore, we benchmark our implementation of simulated annealing in comparison to other implementations of simulated annealing algorithms using diverse hardware technologies. We would like to emphasize that due to the broad differences in hardware implementation, SA task, SA constraints, and SA system size, a strictly quantitative comparison of key efficiency metrics such as solution time or solution energy is not possible. The proposed benchmark compares five different types of hardware: graphic processing units (GPUs), field-programmable gate arrays (FPGAs), CMOS application specific integrated circuits (ASICs), emerging non-volatile memory (eNVM) ASICs based on resistive random-access memories (RRAMs) and conductive bridge random-access memories (CBRAMs),

and, finally, our work based on 2D CMOS circuits. While the first three represent mature, widely adopted technologies, the latter two are still in the early stages of development and remain far from large-scale industrial deployment.

Param \ Tech.	GPU [24]	FPGA [25]	CMOS ASIC [26]	RRAM & CBRAM [27]	2D Memtransistor (This work)
Problem	Max Cut	QUBO	Ising chip demo	Spin Glass	Spatial optimization
I_{max} [mA]	N.A.	N.A.	6	0.2	0.01
I_{min} [mA]	N.A.	N.A.	N.A.	1e-6	10e-12
V [V]	N.A.	(0,0.85)	(0,1.1)*	(-2,2)	(-3,3)
BEOL integration	No	No	No	Yes	Yes
Vertical integration	No	No	No	Yes	Yes
Radiation hardness	No	Medium	No	High	High
Scalability	Limited	Low	Very Low	High	Very High
Drawback	Cost Power	Limited Scalability	Limited Scalability	Process complexity IR-drop	Technology Maturity

Table R1. Benchmarking of SA hardware accelerator technologies. *Benchmarking with various combinatorial problems solved by hardware accelerators of different technology. 2D memtransistors demonstrate highest projected scalability at lowest instantaneous power consumption.*

When comparing mature technologies with emerging technologies such as memristive devices, the focus should primarily be on compatibility for the given task. For example, making a direct power comparison between conventional silicon CMOS technology and memtransistors is inherently difficult: the former is a well-established platform with decades of proven success, whereas the latter has only recently been explored in academic research. Nevertheless, by evaluating fundamental characteristics, such as electrical performance, scalability, and adaptability to different application scenarios, it becomes possible to assess which technology is better suited to the problem at hand. For these reasons, we assembled a comparison shown in **Table R1**. Although the specific optimization tasks differ across platforms, the fundamental operating ranges of their basic hardware elements, such as typical voltages and currents, can still be directly compared. Our devices, for instance, require higher operating voltages but exhibit currents more than an order of magnitude lower than all other technologies considered. Moreover, their operating voltage can be further reduced by decreasing the oxide thickness. Detailed voltage and current specifications for

the transistors used in FPGAs and GPUs are not publicly available. However, at the 20 nm technology node, silicon high-k transistors are known to operate at roughly 1 V with currents in the milliamp range [33]. Moreover, all silicon CMOS-based technologies share one inherent constraint: they are limited to planar integration. In contrast, emerging eNVM technologies, including memtransistors and RRAM/CBRAM, enable computation to scale into the third dimension. As summarized in **Table R1**, both 2D memtransistors (as demonstrated in our earlier works [21, 29–32, 34, 35]) and RRAM/CBRAM provide significant benefits such as back end of line (BEOL) compatibility, vertical integration, radiation tolerance, and lower per-device power consumption. Although RRAM and CBRAM are more technologically mature, they lack intrinsic logic functionality and serve only as fixed resistive elements once programmed. 2D memtransistors instead, while still in early development, offer the potential for monolithic vertical integration of both memory and logic, an essential capability for the applications considered here. The above discussion has been added to the revised manuscript.

2. Please clarify the contribution of this work. What is the main advantage of this work, compared with brute-force searching? Does the advantage mainly come from the algorithm or the circuit implementation? Can approaches based on the conventional CMOS circuits or other memtransistors achieve the same advantages?

The reviewer asks several important questions. We agree that it is crucial to identify and articulate the specific contribution of this work with respect to the algorithm formulation and circuit implementation. The hardware-based annealing accelerator proposed in this manuscript implements the standard simulated annealing algorithm operations of solution generation, energy computation, and solution evaluation following an annealing schedule. The primary advantage of our formulation of the SA algorithm in comparison to other works that utilize SA to solve similar 2D packing and placement tasks is the simplified logic equations that were created to calculate the energy of the prior solution and the proposed solution. These logic equations allow us to seamlessly transition the energy computation step of the SA algorithm into a low-power hardware-based system. We compare the efficiency of our algorithm to brute force searching by calculating the search acceleration, defined as the ratio between the number of SA iterations required to reach the optimal solution and the expected number of brute force trials required to reach the optimal solution. **Equation R5** below computes the expected number of brute force trials required to ensure that the optimal solution is found:

$$BFT = \frac{[(L_E - L_D + 1)^2]^{N_D}}{N_D!} \tag{R5}$$

Here, L_E is the side-length of the environment, L_D is the edge-length of the drone, and N_D is the number of drones. This equation accounts for the multiple global minima allowed by swapping identical drones throughout the solution space. Taking the four-drone (size 4x4), three obstacle 10x10 system evaluated in the submitted version of the manuscript as an example, 5,764,801 brute-

force solution evaluation trials would be required to explore the entire solution space. In comparison, our hardware-based SA accelerator converges in 134 iterations, achieving an ~1800-fold search acceleration. Hardware-realistic simulations of the annealing accelerator reveal that the degree of search acceleration scales favorably with both system size and agent count. We demonstrate enhanced efficiency in larger and more complex configurations with search accelerations up to 1.27×10^{15} in a 640×640 four drone, three obstacle system.

Next, we will elucidate the advantages of using 2D CMOS memtransistors in comparison to a more conventional approach with another material system such as silicon-based field-effect transistors (FETs) that employ floating gate memory. We will separately discuss the TRNG, energy computation, summation, and acceptance modules as each module of the hardware-based annealing accelerator has separate computational requirements. First, the peripheral-free generation of true random numbers enabled by our circuit comprising 21 2D memtransistors is highly energy- and area- efficient in comparison to prior works that exploit frequency collapse in multimodal ring oscillators [1, 2], jitter in ring oscillators [3], or metastability in latches [4]. Next, we would like to note that the energy computation and summation modules of our hardware-based annealing accelerator could be replicated using Si-based FETs. However, logic circuits created with transistors based on 2D transition metal dichalcogenides (TMDs) offer several compelling advantages when compared to logic circuits created with other material systems. Atomically thin TMD films enable excellent electrostatic control of the channel while supporting ultimate body-thickness scaling without mobility degradation [5, 6]. While challenges such as material growth, contact optimization, doping, and performance variability limit the commercial adoption of 2D TMD-based transistors, their increased potential for miniaturization could lead to improved performance and greater energy efficiency at advanced processing nodes. Finally, the thresholding module requires a programmable thresholding circuit. Here, the architecture of 2D TMD memtransistors with charge trapping memory differs from Si-based floating gate memtransistors primarily in the semiconducting channel material. Therefore, similar principles of increased potential for scalability, performance optimization, and energy efficiency apply. This commentary has been incorporated into the revised manuscript.

3. The circuits proposed in this work might require peripheral circuits to perform simulated annealing computation. How do the 2D CMOS circuits interact with off-chip peripheral circuits and the controlling modules?

The reviewer has raised a valid question regarding the interaction of 2D CMOS circuits with off-chip peripheral circuits and controlling modules. In our implementation of simulated annealing, all core operations including random number generation, energy computation, summation, and solution evaluation are performed on-chip by the TRNG, energy computation, summation, and acceptance modules, respectively. The only external peripheral required is a compact memory-

and-control unit (e.g., a microcontroller with local memory), which is inserted between the TRNG module and the energy computation module and is in feedback with the output of energy computation and acceptance modules as illustrated in **Fig. R3**. A representation of the memory configuration is shown in **Fig. R4a** where 10×10 occupancy maps are stored for all four drones (D1–D4) at addresses 00, 01, 10, and 11. One additional map is stored at address X that encodes all stationary obstacles in the environment.

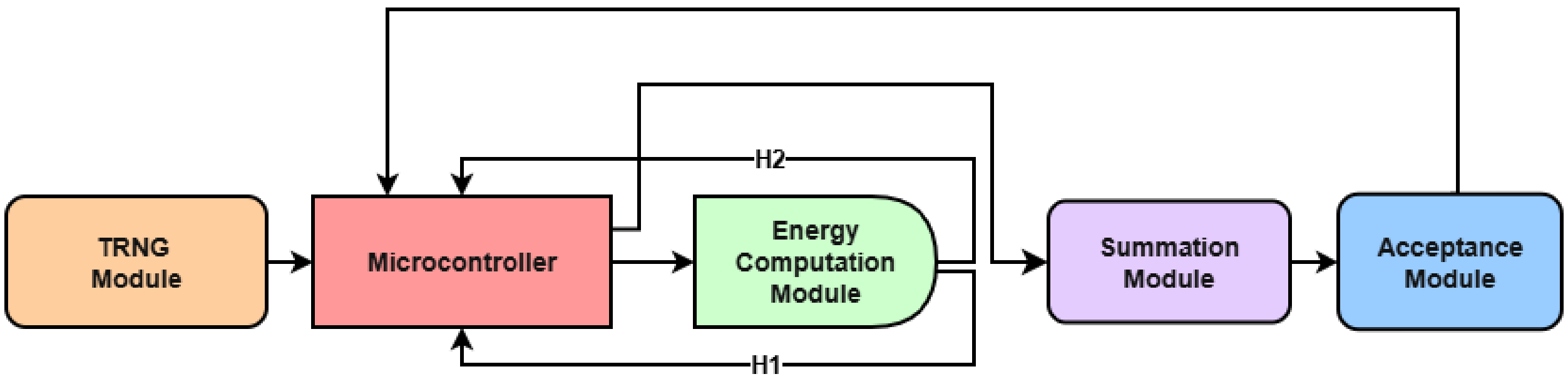

Fig. R3. Interaction of SA Accelerator modules with peripheral circuitry. *Block diagram of the SA accelerator core modules together with the peripheral microcontroller that provides memory and system-level control.*

At each annealing iteration, four bits are generated by the TRNG module and written into the microcontroller memory. The first two bits select the "moving" drone by addressing its stored map. **Fig. R4b** shows a representative example of this operation given random bits '01' where the map at address 01 corresponding to D2 is designated as the active map, M, and a copy is written into the next available memory location, Y. The remaining two bits encode the proposed motion of the selected drone (00 = up, 01 = down, 10 = left, 11 = right). This motion is applied to M to generate M′ which is stored in memory location Z, as illustrated in **Fig. R4c**. At this stage, six maps are streamed to the energy computation module: M and M′ for the original and proposed occupancy maps of the moving drone, D1, D3, and D4 for the three stationary drones (S1, S2, and S3), and O for the static obstacles. Then, two additional memory addresses are created to store the outputs of energy computation module, H_{Prior} and H_{New}. Next, H_{Prior} and -H_{New} vectors are sequentially given to the summation module. The output of the summation module is sent to the acceptance module. The resulting 1-bit acceptance signal is fed back to the microcontroller. **Fig. R4d** shows the resulting memory update that occurs if the acceptance signal is 1, indicating that proposed drone movement was accepted. In this case, the occupancy map at address 01 corresponding to D2 is updated with the approved movement, M′. **Fig. R4e** illustrates that if the acceptance module output signal is 0, the proposal is rejected and the occupancy map at address 01 corresponding to D2 remains unchanged. In this manner, the full simulated-annealing loop is realized with a single, well-defined memory/control peripheral tightly integrated around the existing modules. The proposed design requires only memory and simple control logic. These functionalities can be directly implemented on a custom printed circuit board with a basic microcontroller that minimizes additional energy overhead. Additional discussion regarding the interaction of 2D CMOS circuits with off-chip peripheral circuits and controlling modules has been added to the revised manuscript.

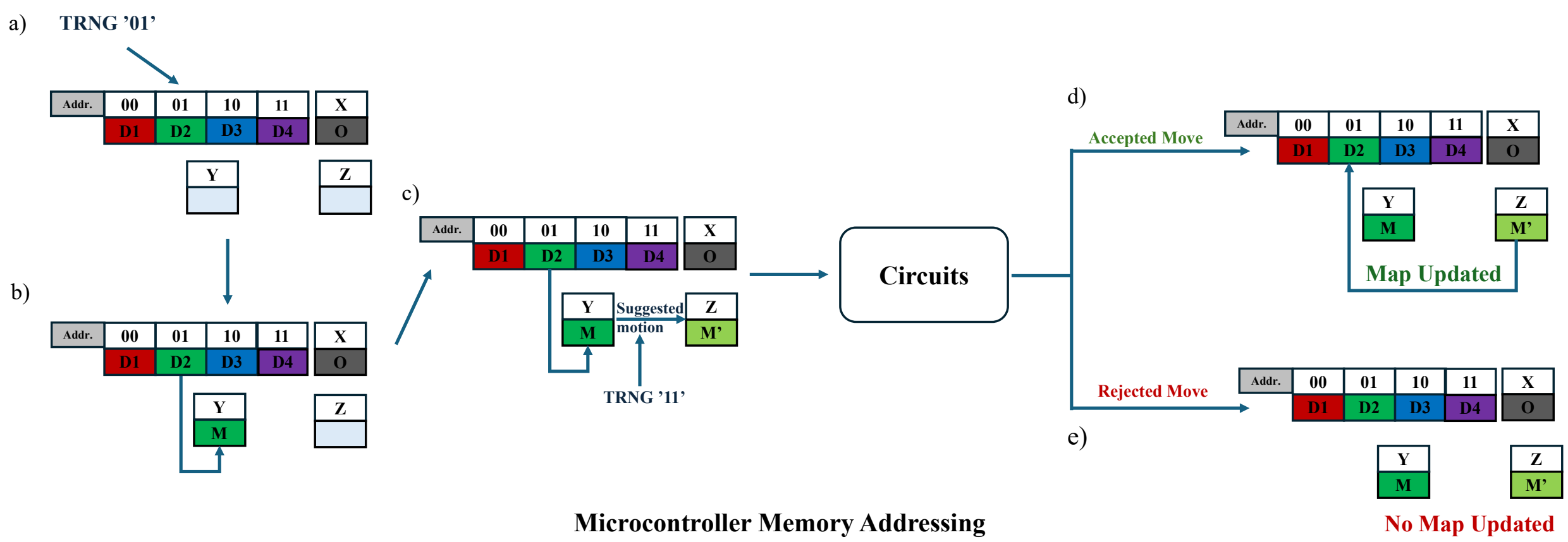

Fig. R4. Microcontroller Memory Addressing. *Memory configuration of the microcontroller given the **(a)** initial state of the system comprising 4 drones and 1 obstacle. **(b)** Microcontroller memory operation where a map at address 01 corresponding to D2 is designated as the active map, M, and a copy is written into the next available memory location, Y. **(c)** Storage of M' in memory location Z. Microcontroller memory operation **(d)** if the proposed move is accepted and **(e)** if the proposed move is rejected.*

4. This work presents the TRNG results in Figure 2. Could the TRNG module in this work pass the NIST test? How do these characteristics influence the simulated annealing results?

The TRNG bitstreams used in this work were generated by an architecture comprising two cascaded three-stage inverters (TSIs) connected to a XOR gate, as detailed in our prior work [12]. The TSIs and XOR gate were all implemented using monolayer MoS_2-based field-effect transistors (FETs) with atomic layer deposition (ALD) grown Al_2O_3 as the gate dielectric. The dual-sweep voltage transfer characteristics of a representative TSI are shown in **Fig. R5a**. Random temporal fluctuations can be observed by sampling the TSI at an appropriate input voltage, $V_{Sampling}$, within the hysteresis window between the forward and reverse voltage sweeps. A representative subset of 10^5 bits sampled at 5 ms intervals is shown in **Fig. R5b**. The observed fluctuations originate from the stochastic trapping and detrapping of charges at and/or near the Al_2O_3/MoS_2 interface. One of the key properties of our TRNG is the balanced distribution of output bits. The proportion of 0's and 1's for this representative subset of 10^5 bits is approximately equal, as shown in **Fig. R5c**. We sampled 10^6 random bits and evaluated their randomness using the National Institute of Standards and Technology (NIST) SP 800-22 statistical test suite. Each test in this suite generates a p-value which is evaluated at a 99% confidence level. Bitstreams with a p-value greater than 0.01 can be considered random. **Table R2** shows that the generated bit stream passes all 15 NIST SP 800-22 tests without any post-processing.

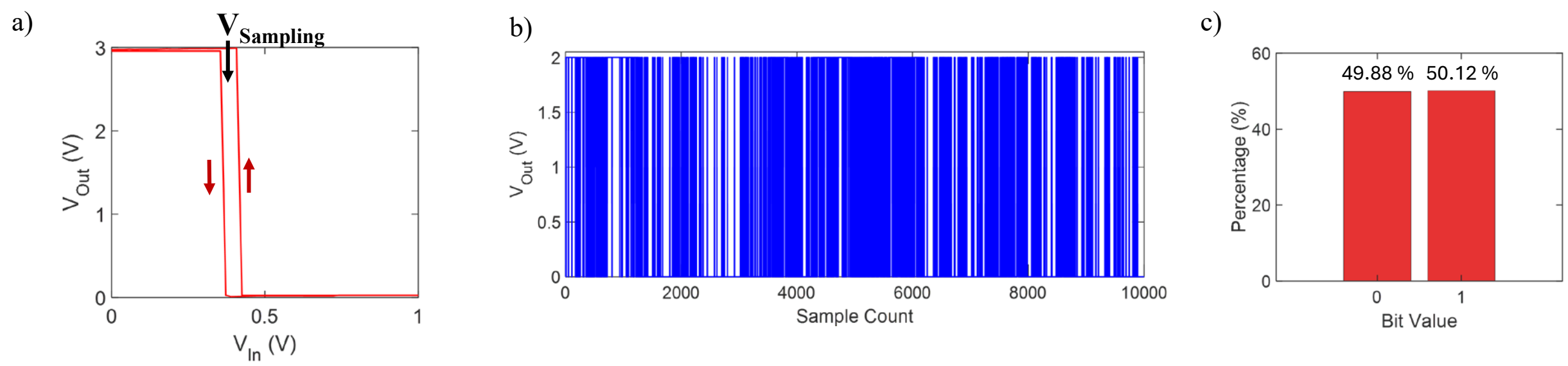

***Fig. R5. TSI TRNG Characterization. (a)** Output voltage, V_{Out}, measured as a function of input voltage, V_{In}, for a three-stage inverter. Random temporal fluctuations can be observed by sampling the three-stage inverter at an appropriate input voltage, $V_{Sampling}$, within the hysteresis window between the forward and reverse voltage sweeps. **(b)** A representative subset of 10^5 bits sampled at 5 ms intervals and **(c)** the resultant distribution of 0's and 1's.*

NIST SP 800-22		
Statistical test	Sequence 1	
	p-value	Result
Frequency monobit	0.321	PASS
Block frequency	0.579	PASS
Cumulative sums (forward)	0.526	PASS
Cumulative sums (backward)	0.397	PASS
Runs	0.626	PASS
Longest run of ones	0.412	PASS
Rank	0.349	PASS
FFT	0.103	PASS
Non-overlapping Template	0.511	PASS
Overlapping Template	0.116	PASS
Approximate entropy	0.519	PASS
Universal	0.107	PASS
Random excursion	0.469	PASS
Random excursion variant	0.550	PASS
Serial	0.314	PASS
Linear complexity	0.273	PASS

***Table R2. NIST SP 800-22.** NIST SP 800-22 comprises 16 tests that were used to evaluate a set of one million randomly generated bits. All tests result in a p-value greater than 0.01 which confirms the true randomness of the evaluated bits.*

We quantitatively assess the influence of deviations from ideal randomness on the annealing dynamics by artificially modifying the random bitstream used to guide drone selection and movement. First, we constructed eight non-ideal bitstream ensembles with proportions of ones ranging from 10% to 90%. These biased streams were generated by randomly flipping bits from the original balanced sequence with ~50% ones to achieve the desired imbalance. For each bias level, we performed simulated annealing on a representative 20×20 four drone, three obstacle system with 24 permissible solutions. For each of the nine bitstreams with varying proportions of ones, 30 different SA initializations were performed. Each SA algorithm followed an annealing schedule comprising 120 iterations per temperature at six descending temperatures for a total of 720 iterations. For a fair comparison, we have used identical initial drone positions across all different bitstreams such that only the random bits used for optimization change between initializations. We recorded the evolution of system overlap as a function of SA iterations. convergence trajectories illustrating the system overlap averaged across all 30 initializations for each bitstream variation are shown in **Fig. R6a**. The convergence rate, defined as the percentage of initializations that reach a permissible solution, is shown as a function of the proportion of ones in the random bitstream in **Fig. R6b**. As expected, the convergence rate is maximized when the random bitstream contains an ideal ~50% proportion of ones. These results demonstrate that an intrinsically balanced bit distribution provides the most effective source of stochasticity for the annealing circuit, thereby enhancing convergence behavior and overall algorithm efficiency.

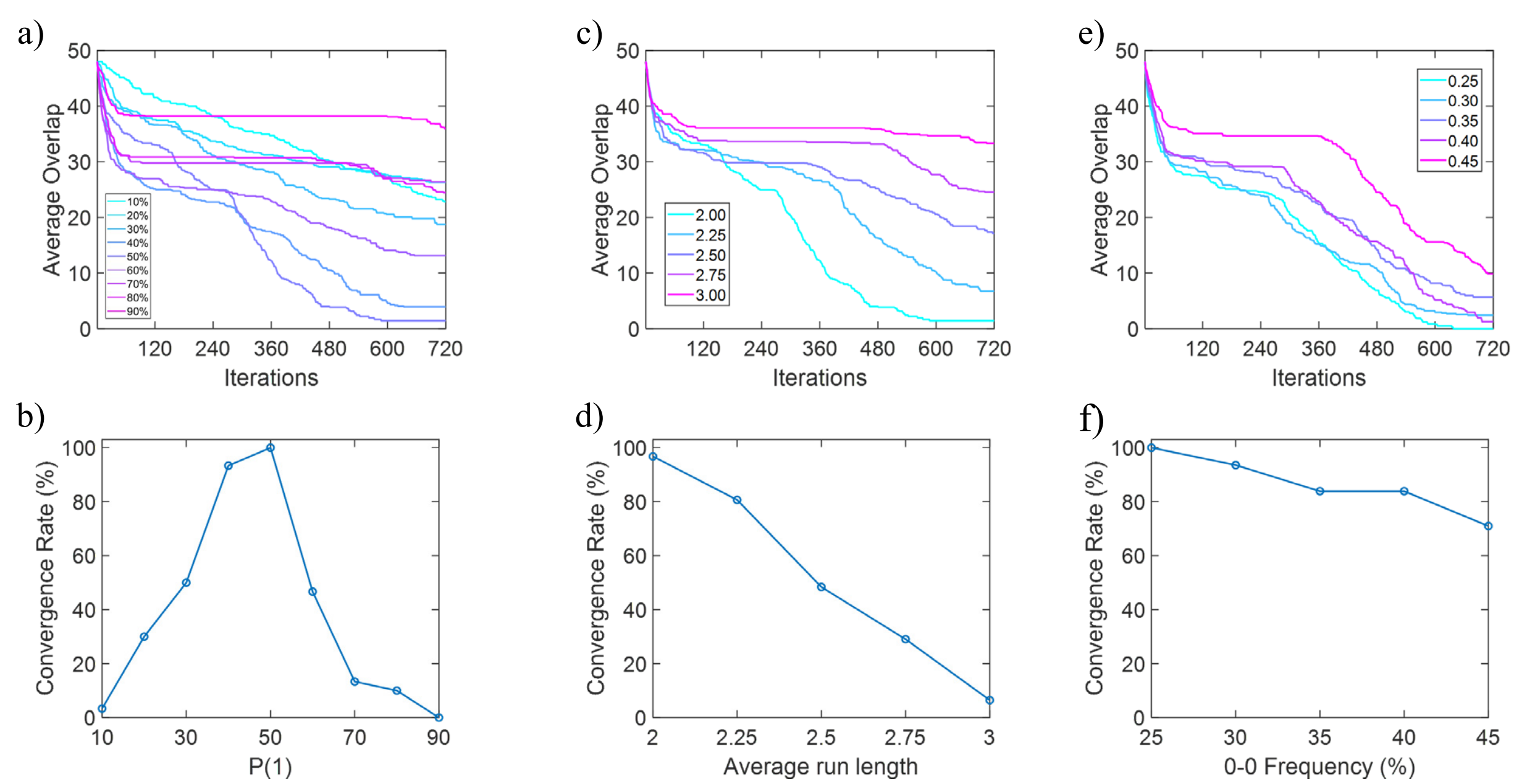

Fig. R6. Impact of non-ideal bitstream variations on SA convergence. *Average system overlap and convergence rate, respectively, for bitstream variants with* ***(a-b)*** *different proportions of 1's,* ***(c-d)*** *different average run lengths, and* ***(e-f)*** *different 0-0 combination frequencies. system overlap was averaged across 30 SA initializations for each bitstream variant.*

The average run length (or duration) of 0's and 1's in a bitstream is another important figure of merit for a random bitstream. For our TSI-based TRNG bitstream, the mean run length of both 0's and 1's was measured to be ~2. Starting from this experimentally obtained metric, we synthetically generated four additional bitstreams with controlled average run lengths of 2.25, 2.5, 2.75, and 3, thereby spanning five total bitstream configurations. For each of these run-length configurations, we repeated the same annealing performance analysis described above. **Fig. R6c** shows the overlap averaged across all 30 initializations as a function of iteration number for all five run-length bitstream configurations while **Fig. R6d** plots the convergence rate as a function of average run length. The bitstream with an average run length of 2, corresponding to our unadulterated TRNG bitstream, yields the largest number of converged solutions across 30 independent random initializations. Larger run lengths systematically reduce the convergence rate. These results highlight that introducing longer contiguous strings of 0's and 1's degrades the performance of the simulated annealing algorithm.

Finally, the serial test investigates the frequency of occurrence of the 2^m different m-bit sequences. When m=2, there are four 2-bit combinations: 0-0, 0-1, 1-0, and 1-1. For our TRNG bitstream, the frequency of occurrence of each 2-bit combination was nearly ideal at ~25%. We synthetically generated five additional modified bitstreams by varying the frequency of occurrence of the 0-0 combination to 30%, 35%, 40%, 45%, and 50%. These bitstreams were generated by randomly converting 0-1, 1-0, and 1-1 combinations into 0-0 combinations at equal proportions. For each of these 2-bit combination frequency configurations, we repeated the same annealing performance analysis described above. **Fig. R6e** shows the overlap averaged across all 30 initializations as a function of iteration number for all six bitstream configurations while **Fig. R6f** plots the convergence rate as a function of 0-0 combination frequency. The bitstream with an average 0-0 frequency of 25%, corresponding to our unadulterated TRNG bitstream, yields the largest number of converged solutions across 30 independent random initializations. Clearly, higher frequencies of 0-0 combinations yield reduced convergence rates. It is evident that true random numbers enable higher convergence rates than the biased bitstream variants. These results highlight the necessity of high quality TRNG bits. The revised manuscript incorporates **Table R2** and a detailed evaluation of the effect of TRNG variation on the performance of the annealing algorithm.

5. Please illustrate the bandwidth or generating speed of the TRNG module, since it could influence the computing speed of the system. It would also help if the authors could characterize the entropy source of this work.

The current generation speed of the TRNG is 200 Hz. **Fig. R7** shows a timing diagram of the TRNG module including the supply voltage, V_{DD}, the input voltage, V_{IN}, and the output signal, V_{OUT}. Here, V_{OUT} is sampled with $V_{\mathrm{IN}} = V_{\mathrm{DD}}/2$ for 300ms. Notably, the produced output values toggle randomly between the two states, 0 and 1. We characterize the entropy source of this work

using the NIST SP 800-90B statistical test suite. This test suite determines if an entropy source is independent and identically distributed (IID) or non-IID before estimating the minimum entropy.

The results of the test suite after non-IID classification are shown in **Table R3**. Here, the compression test calculates a minimum entropy of .8615. The TRNG module achieves an energy cost of only 30 pJ/bit and operates without any external peripherals or digital post-processing. **Table R3**, **Fig. R7**, and corresponding discussion regarding the entropy source of this work have been added to the revised manuscript.

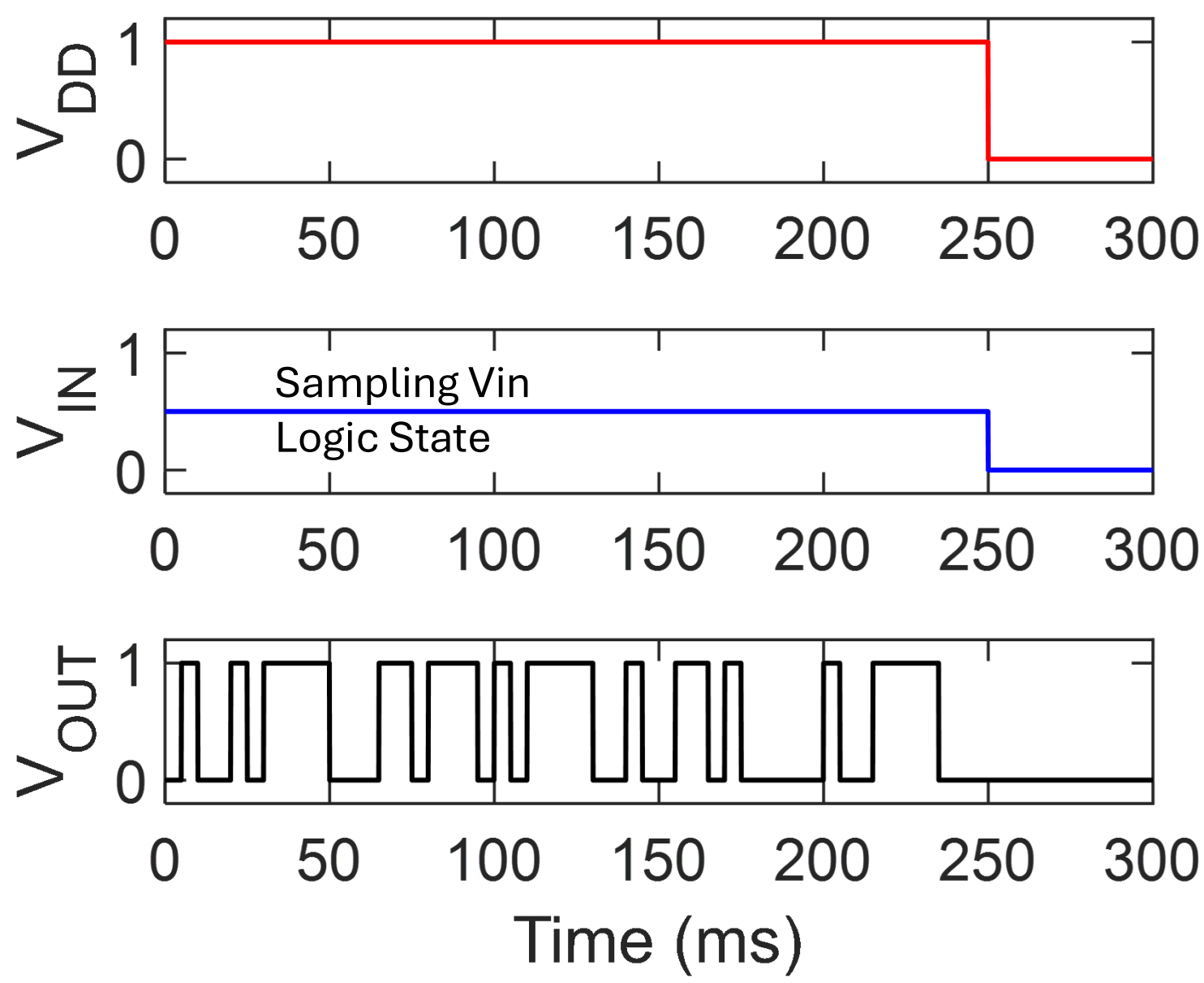

Fig. R7. Timing response of TRNG. *Timing diagram of the TRNG module including the supply voltage,* V_{DD}*, the input voltage,*V_{IN}*, and the output signal,* V_{OUT}.

NIST SP 800-90B	
Non-IID Test	Entropy Rate Sequence 1
Most common value	0.9949
Collision	0.9248
Markov	0.9979
Compression	0.8615
t-Tuple	0.9381
Longest repeated substring	0.9921
Multi most common	0.9943
Lag prediction	0.9951
Multi MMC prediction	0.9977
LZ78Y prediction	0.9988
Minimum Entropy	***0.8615***

Table R3. NIST SP 800-90B. *NIST SP 800-90B test suite used to characterize the entropy source.*

6. It requires a timing diagram that shows how TRNG and other circuit components are incorporated in the system. The bitstreams shown in Figure 3 might be confusing to the readers. It would be better if there were more explanations.

For ease of reference and discussion, we have separated this comment into two different sections.

6a. It requires a timing diagram that shows how TRNG and other circuit components are incorporated in the system.

We thank the reviewer for this suggestion. We agree that timing diagrams illustrating the operation of each circuit module would be a valuable addition to the revised manuscript. The circuit schematic of the entire hardware-based simulated annealing accelerator is shown in **Fig. R8** for ease of reference throughout the discussion on the timing diagrams of each individual circuit module.

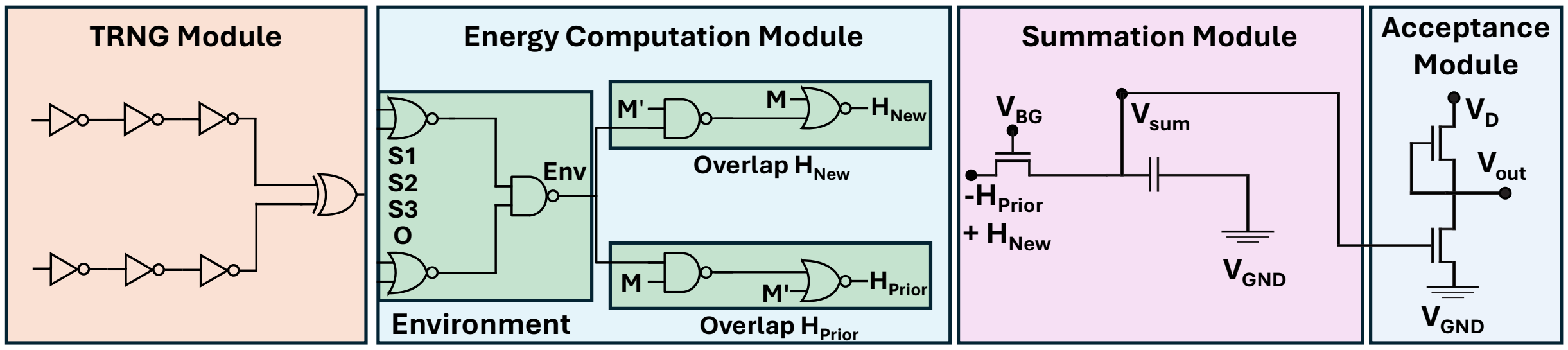

Fig. R8. Circuit Implementation of Hardware-based SA accelerator. *Circuit schematic of all hardware modules utilized to accelerate SA for constrained optimization including the TRNG module, the energy computation module, the summation module, and the acceptance module.*

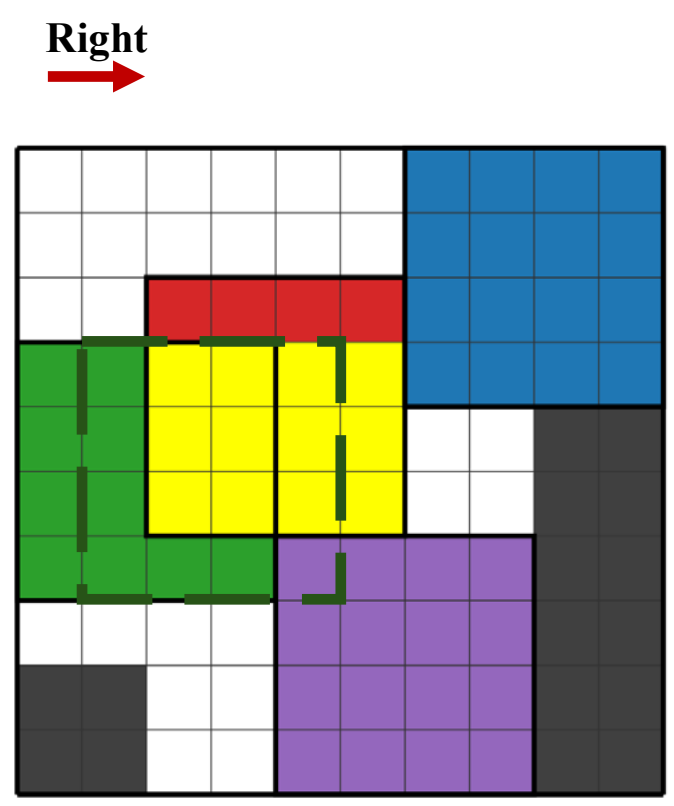

Fig. R9. Proposed motion of green drone used for timing analysis. *Map of the initial occupancy and proposed occupancy of the green moving drone. The solid green box is representative of the initial occupancy and the dashed green line indicates the proposed occupancy.*

The timing diagrams of the circuit will be shown in response to a representative rightward movement of the green drone from the system shown in **Fig. R9**. **Fig. R10a** shows the timing diagram of the TRNG module, where a random bit is sampled every 5 ms. Four total bits are required per simulated annealing iteration to select the drone and its proposed movement direction, resulting in a total of 20 ms per iteration for this module. In the example shown, the generated sequence is ‘0, 1, 0, 1’. For illustrative purposes we will assume that the first pair (‘0, 1’) selects the green drone, while the second pair (‘0, 1’) corresponds to a rightward proposed motion. Next, **Fig. R10b** presents the timing response of the energy computation module, which comprises three NAND and four NOR operations. First, the environment occupancy map, E, is computed as the sum of the stationary drones S1, S2, S3 and the obstacle, O, as shown in **Equation R6**:

$$E = S_1 + S_2 + S_3 + O \tag{R6}$$

We implement the above equation with combinational logic comprising two NOR operations and one NAND operation as follows:

$$A = \overline{S_1 + S_2} \tag{1}$$

$$B = \overline{S_3 + O} \tag{2}$$

$$E = \overline{A * B} \tag{3}$$

In this implementation, the first two NOR operations are evaluated in parallel to produce intermediate node voltages A and B within ~2 s operating at 20 ms per input over 100 inputs corresponding to the entire occupancy map. Intermediate voltages A and B are given to a final NAND gate which computes E with identical timing properties. Next, the overlap unique to the prior solution, H_{Prior}, and the overlap unique to the new solution, H_{New}, are computed with using fundamental logic gates following **Equation R7** and **Equation R8**:

$$H_{Prior} = E * M * \overline{M'} \tag{R7}$$

$$H_{New} = E * \overline{M} * M' \tag{R8}$$

the prior and new energies are calculated with combinational logic comprising cascaded NAND and NOR operations as follows:

$$C = \overline{E * M'} \tag{1}$$

$$D = \overline{E * M} \tag{2}$$

$$H_{Prior} = \overline{D + M'} \tag{3}$$

$$H_{New} = \overline{C + M} \tag{4}$$

Here, two parallel two-input NAND gates generate C and D in 2 s. Finally, two parallel NOR gates produce H_{Prior} and H_{New}. Thus, the total time for the energy computation module is approximately 2 s per iteration. Next, **Fig. R10c** shows the timing diagram of the summation module, which computes the net energy change by adding -H_{Prior} and H_{New}, yielding a positive V_{Sum} of ~1V in

the case shown due to the fact that H_{New} contains four high values and H_{Prior} does not contain any values. This circuit is sampled every 30 ms and processes 200 inputs per iteration, resulting in a total duration of 6 s for the summation step. **Fig. R10d** depicts the acceptance stage, implemented using an inverter operating at a 30 ms sampling rate. Assuming operation at a temperature corresponding to an inverter threshold of 750 mV, the input $V_{Sum} = 1\ V$ results in a logical output $V_{Out} = 0\ V$ indicating that the proposed move was rejected.

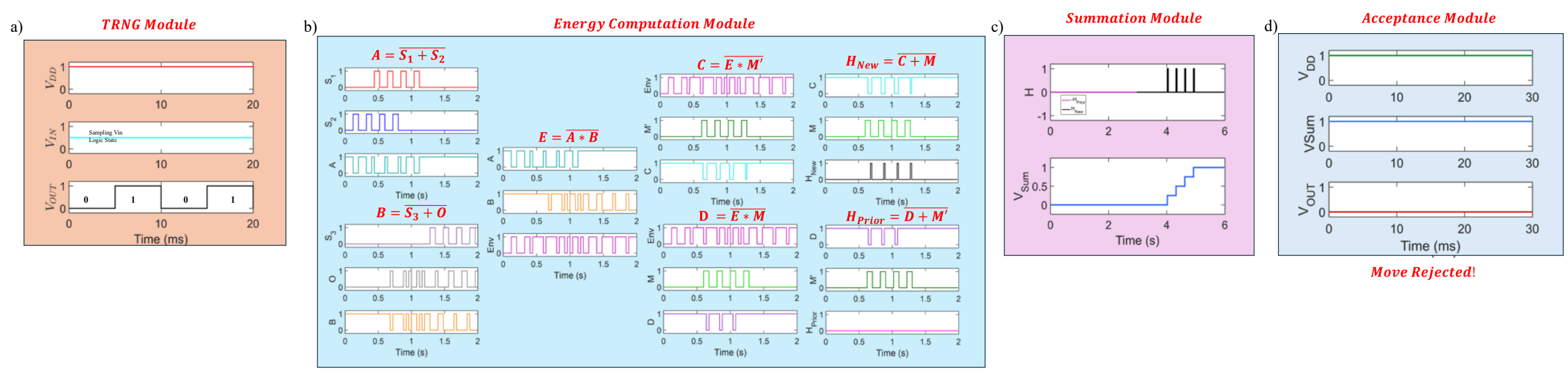

Fig. R10. Timing response of all circuit modules used for SA. *The timing responses for one proposed rightward movement of the green drone for the **(a)** TRNG module sampled at 5ms, **(b)** energy computation module sampled at 20ms, **(c)** summation module sampled at 30ms, and the **(d)** acceptance module sampled at 30ms.*

The time required for each module to complete its respective operations during each iteration is summarized in **Fig. R11**. At present, the overall computation speed is limited by the summation module, resulting in an effective computation rate of approximately 0.124 Hz per iteration. Although this speed is relatively low, it can be significantly improved by increasing the measurement speed. Our prior works have demonstrated logic operations at frequencies up to 25 kHz [13], which would reduce the energy computation time per iteration to 4 ms. Furthermore, the summation time can be lowered by sampling at 25 kHz with an appropriate capacitor choice in conjunction with the MoS_2 transistor, which would result in a summation time of approximately 8 ms per iteration. With these projected improvements, the per-iteration timing would be approximately 20 ms for the TRNG module, 4 ms for energy computation module, 8 ms for summation module, and 5 ms for the acceptance module, yielding a total of 37 ms per iteration (~27 Hz). For the system demonstrated experimentally in the submitted version of the manuscript requiring 150 iterations, this corresponds to a convergence time on the order of 5.6 s. Finally, we note that a typical microcontroller operates at MHz frequencies and therefore would have a negligible impact on the overall system speed. The circuit timing diagrams and related discussion have been added to the revised manuscript.

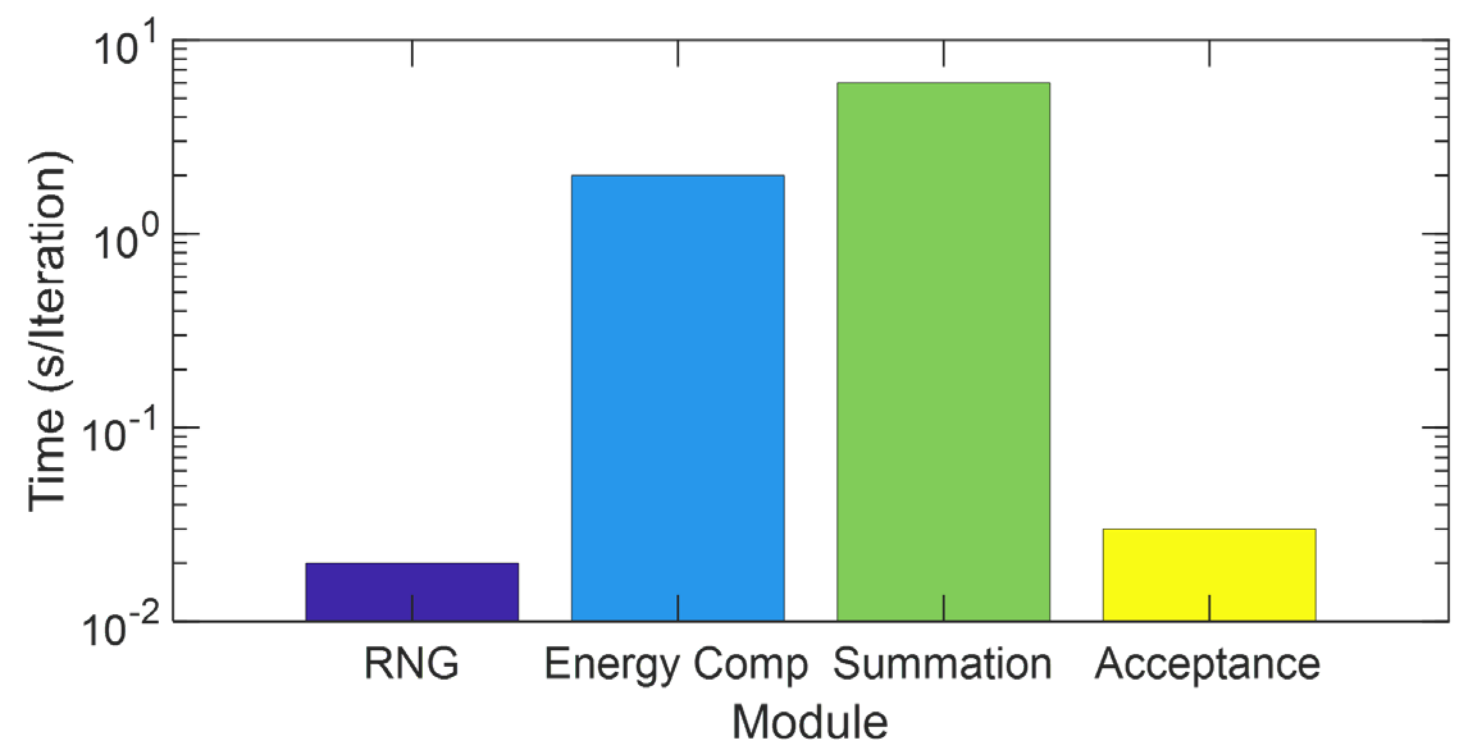

Fig. R11. Timing comparison of circuit modules used for SA. *Bar plot of total time taken per module for one iteration of the simulated annealing algorithm.*

6b. The bitstreams shown in Figure 3 might be confusing to the readers. It would be better if there were more explanations.

We thank the reviewer for this suggestion that will aid in the readability of the paper. **Fig. R12** illustrates the row-wise flattening procedure that was used to transform binary occupancy maps into flattened bitstreams. **Fig. R12** and additional discussion on the drone vectorization procedure has been added to the revised manuscript.

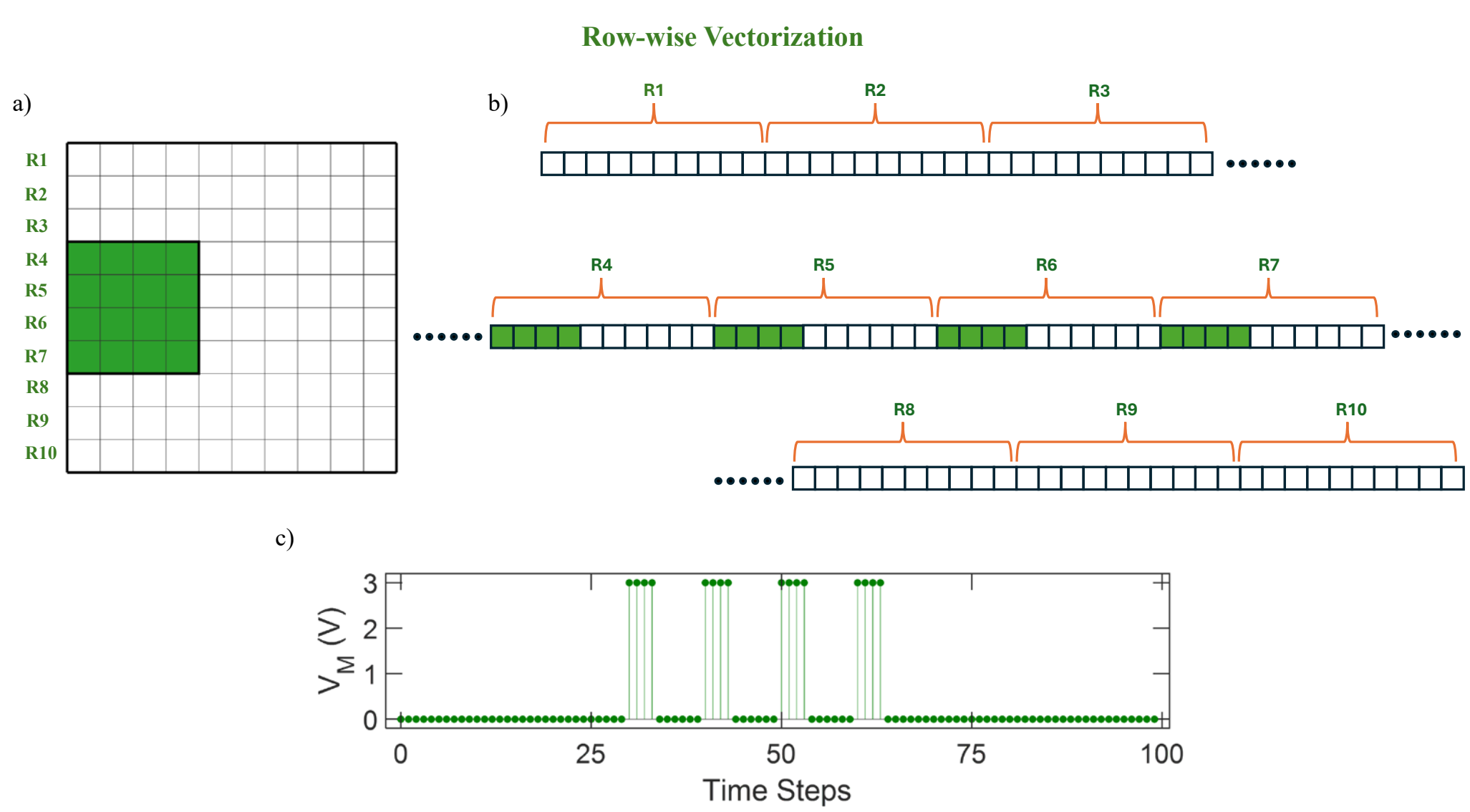

Fig. R12. Drone map vectorization. (a) *10×10 map of a representative 4×4 drone.* ***(b)*** *Row-wise vectorization of the 10x10 map.* ***(c)*** *Stem plots corresponding to row-wise vectorized drone map.*

7. Please also illustrate whether this work can generalize to other constraint satisfaction problems.

As discussed in the response to comment 1b, the constrained spatial optimization problem that we address can be broadly represented as a 2D packing and placement task. The formulation of this problem is similar in structure to very-large-scale integration (VLSI) floorplan design tasks where fixed-footprint objects must be positioned on a grid without overlap while respecting forbidden regions. The approach utilized in this work could potentially be generalized to other constraint satisfaction problems set in a discrete spatial domain with arbitrary constraints. For example, the eight queens problem could be solved by initializing agents of the shape described by the allowable movement regions of a queen chess piece. By minimizing overlap between agents, the system would potentially converge to a permissible solution. Nonetheless, the exact circuit implementation of our work cannot be generalized to constraint satisfaction problems such as graph coloring, max-cut, or sudoku. However, only the energy computation and summation modules of the hardware-based annealing accelerator would require modification to create a hardware-based annealing accelerator capable of solving these problems. The remaining TRNG and acceptance modules would not require modification as they perform general purpose annealing operations. This discussion has been added to the revised manuscript.

8. The authors show the evolution process in this work regarding the iterations or time steps. The readers may also be concerned about the real-time computing speed of the proposed circuits. It would be helpful if the authors could provide more characteristics of the proposed transistors and circuits on timing response.

We thank the reviewer for this suggestion and we agree that the readers may be concerned about the real-time computing speed of the proposed circuits. We have discussed the timing response of each module of the circuit in our response to comment 6a. Please let us know if any additional commentary or specific analysis regarding the timing response of the circuits or proposed transistors would be beneficial to the revised manuscript.

Reviewer #2 (Remarks to the Author):

This work demonstrates a hardware accelerator for simulated annealing (SA) using 2D-material-based CMOS circuits. It demonstrates spatial optimization for drone placement with obstacles problems efficiently. The system achieves ~1800x speedup over brute-force search with low energy consumption, leveraging stochasticity and programmability of 2D devices. However, there are still several critical comments that need to be addressed:

1. The key drawback of this work is that the implementation of SA for constraint satisfaction problems appears to be done by pure simulation without experimental verifications. It is unclear to what extent the actual device behaviors (including the non-ideal characteristics) and peripheral circuits are taken into consideration in the system-level benchmark.

We thank the reviewer for their detailed comments and suggestions. We would like to note that experimental verification of the entire hardware-based simulated annealing accelerator was performed on the four-drone, three-obstacle 10x10 system described in the introduction of the submitted manuscript. After establishing the validity of the hardware-based annealing accelerator over one SA iteration in **Fig. 3** of the submitted manuscript, the same drone system was evaluated in hardware with an annealing schedule comprising 30 iterations per temperature at five descending temperatures for a total of 150 iterations. **Fig. 4a-e** of the submitted manuscript show the state of the hardware-generated solution at 5 different iterations (1, 2, 3, 4, and 5) for all five temperatures. **Supplementary Video 1** in the revised manuscript shows the evolution of the system over all 150 iterations. Clearly, the annealing accelerator explores the solution space at higher temperatures before converging to an optimal solution by the 14th iteration of the final temperature.

Nonetheless, we believe that a thorough evaluation of device related non-idealities and their impact on the annealing operations would be a valuable addition to the revised manuscript. To quantify the deviation of each hardware block from its ideal value, we employ the hardware-realistic simulation. Ideal values for the output of the energy computation module, the summation module, and the acceptance module were determined utilizing an identical initialization of the four-drone, three-obstacle 10x10 system and the experimentally realized string of TRNG bits. The experimental and ideal values of H_{Prior} and H_{New} are shown for all 150 simulated annealing iterations in **Fig. R13a-b**, respectively, alongside the module errors. Note that 15,000 total time steps are shown corresponding to 100 time steps per iteration. Comparison of experimental and ideal values of H_{Prior} and H_{New} reveals that the 2D CMOS based logic circuits exhibit negligible error. Next, the experimental and ideal outputs of the summation module are shown alongside the module error for all 150 simulated annealing iterations in **Fig. R13c**. During each iteration, this module sums 200 values corresponding to -H_{Prior} and H_{New}. The mean absolute error (MAE) of this module is approximately 189.9 mV. Here, it is important to note that despite the MAE of 189.9 mV, the hardware-based annealing accelerator does not deviate from the ideal movement decisions determined by the hardware-realistic simulation. Subsequently, the experimental and ideal outputs of the acceptance module are shown alongside the module error for all 150 simulated annealing iterations in **Fig. R13d**. The acceptance module error is negligible and will not impact subsequent logic operations. Finally, we don't consider the micro-controller unit as it does not do any computation on the occupancy maps. Thus, the peripherals do not have an impact on the convergence of the algorithm. Discussion regarding device related non-idealities and their impact on the annealing operations has been presented in the revised manuscript.

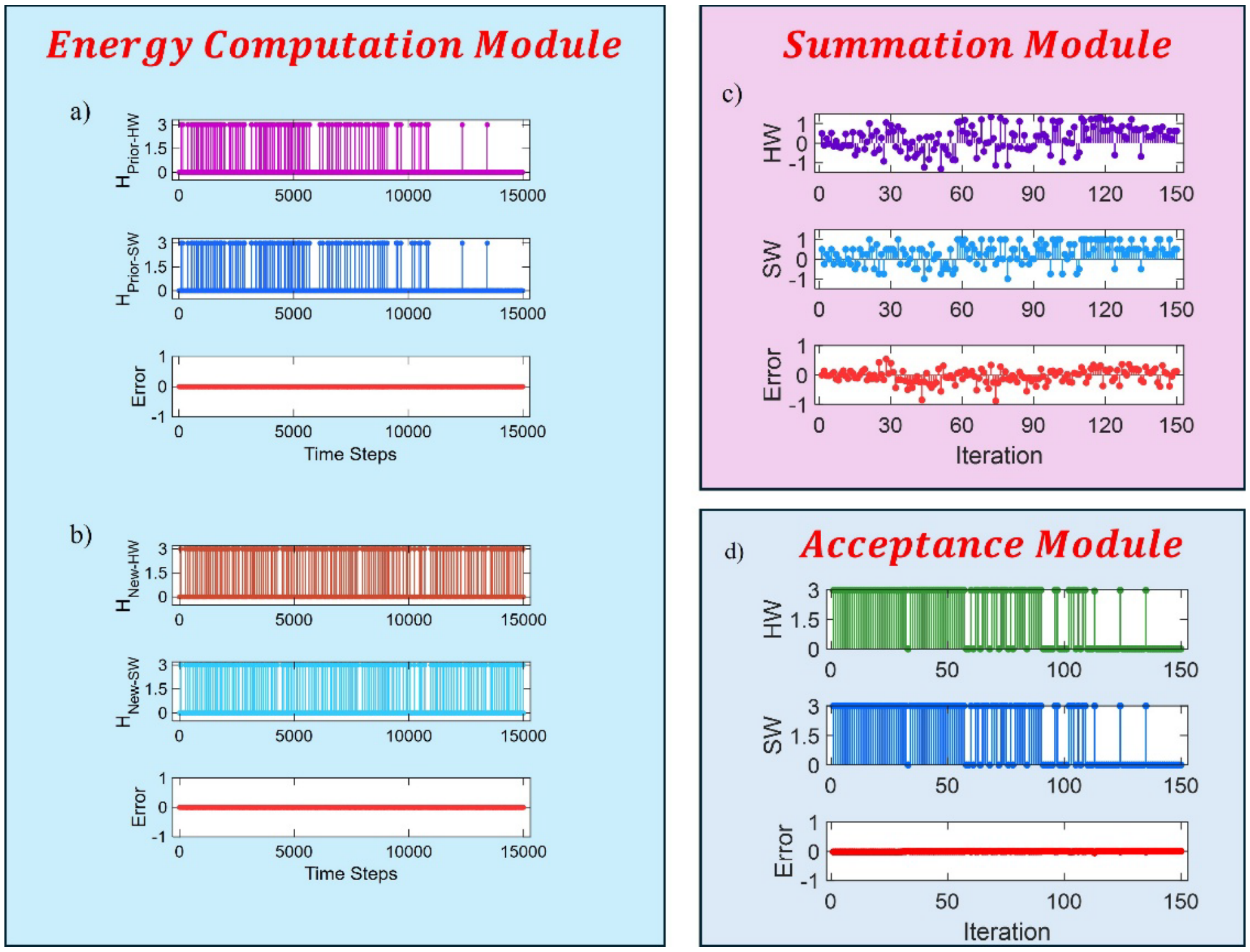

Fig. R13. Characterization of experimental error. *Experimental and ideal values of* ***(a)*** H_{Prior} *and* ***(b)*** H_{New} *are shown alongside the module errors for all 150 simulated annealing iterations. Experimental and ideal outputs of the* ***(c)*** *summation and* ***(d)*** *acceptance modules alongside the module errors for all 150 simulated annealing iterations.*

2. The Introduction mentions prior acceleration efforts using GPUs, FPGAs, and RRAM/CBRAM devices but remains largely qualitative. A quantitative comparison table summarizing key metrics (e.g., energy efficiency, processing speed, area efficiency) across these platforms would provide valuable context and highlight the advantages of the proposed approach more clearly.

We thank the reviewer for this suggestion. We agree that comparing different hardware approaches for accelerating simulated annealing would enrich the discussion in the revised manuscript. However, establishing a fair, quantitative comparison across distinct optimization problems is not feasible, especially when the underlying computation systems differ substantially in area and power consumption. Although a unified benchmarking framework is therefore difficult to construct, it is still important to discuss the advantages and drawbacks of using each technology for simulated annealing. Graphics processing units (GPUs) and field-programmable gate arrays (FPGAs) can be reconfigured to address multiple problem types, whereas application-specific integrated circuits (ASICs) generally cannot. Conversely, for any specific optimization task, ASICs will almost invariably outperform GPUs and FPGAs, which suffer from significantly higher power

consumption. It is also important to consider how each technology scales with problem dimensionality, that is, its capacity to handle optimization problems with a very large number of degrees of freedom. GPUs have shown promise in accelerating combinatorial optimization problems [14], yet their power efficiency remains poor compared with that of ASICs. FPGAs offer a high degree of reconfigurability [15-18], but their achievable clock frequency, available lookup tables (LUTs), and block RAM (BRAM) size impose practical limits on scaling to high-dimensional problems. CMOS ASICs, by contrast, can be engineered for substantially larger and more complex tasks [16], though their scalability is ultimately constrained by the lack of vertical integrability.

These limitations have contributed to recent interest in near-memory and in-memory computing with memristive devices [19]. One representative example employs resistive random-access memory (RRAM) and conductive-bridge random-access memory (CBRAM) to build an application-specific accelerator [17]. While this approach efficiently addresses a Spin Glass problem via in-memory matrix–vector multiplication (MVM) and stochastic switching, the relatively large operating currents of emerging non-volatile memories (eNVMs) lead to non-negligible voltage drops across crossbar parasitic resistances [20], reducing the effective conductance of the device and thus degrading computational accuracy and increasing power consumption. Combining two different eNVM technologies on the same chip also adds fabrication complexity and cost. In contrast, our work seeks to leverage the advantages of two-dimensional materials. These materials offer exceptional scalability, supporting semiconducting channels at atomic thickness while maintaining high carrier mobility. Their growth has been demonstrated to be both back-end-of-line compatible [21] and vertically integrable [22]. Taken together, these characteristics point toward the potential capability of 2D-material-based ASICs to address much larger optimization problems than current alternatives.

To better evaluate the strengths and limitations of the various technologies, we compiled a comparison table shown in **Table R4**. Although the underlying optimization tasks differ, making a direct energy or power comparison impractical, we can meaningfully contrast the typical voltage and current ranges of the corresponding hardware, since these two parameters strongly influence overall performance. Our system operates at higher voltages, but the associated currents are significantly lower than in the other platforms, effectively balancing the voltage requirement. Further reductions in operating voltage are also achievable by decreasing the oxide thickness of our devices. While detailed voltage and current ranges for the transistors used in FPGAs and GPUs are not publicly accessible, it is known that at the 20 nm technology node, silicon high-k transistors typically operate at approximately 1 V with currents on the order of milliamps [23]. Additionally, the GPU used in the referenced study has a theoretical maximum power consumption of 245 W [24] and a cost at the time of release of roughly eight thousand dollars, figures that are clearly prohibitive for scalable or widespread deployment. All silicon CMOS based technologies also share an intrinsic limitation: they are confined to 2D integration. In contrast, emerging eNVM

technologies, including memtransistors and RRAM/CBRAM, offer the ability to scale computation into the third dimension. As shown in **Table R4**, both 2D memtransistors (as demonstrated in our previous works [11, 19-22, 25, 26] and RRAM/CBRAM exhibit compelling advantages such as BEOL compatibility, vertical integration, radiation tolerance, and reduced per-device power consumption. While RRAM and CBRAM are more mature technologies, they lack intrinsic logic capabilities; once programmed, they function solely as fixed resistive elements. In comparison, 2D memtransistors are still far from industry level standards, but they will be able to vertically integrate both memory and logic which is an essential requirement for this class of applications. The above comparisons have been added to the revised manuscript.

Tech. / Param	GPU [24]	FPGA [25]	CMOS ASIC [26]	RRAM & CBRAM [27]	2D Memtransistor (This work)
Problem	Max Cut	QUBO	Ising chip demo	Spin Glass	Spatial optimization
I_{max} [mA]	N.A.	N.A.	6	0.2	0.01
I_{min} [mA]	N.A.	N.A.	N.A.	1e-6	10e-12
V [V]	N.A.	(0,0.85)	(0,1.1)*	(-2,2)	(-3,3)
BEOL integration	No	No	No	Yes	Yes
Vertical integration	No	No	No	Yes	Yes
Radiation hardness	No	Medium	No	High	High
Scalability	Limited	Low	Very Low	High	Very High
Drawback	Cost Power	Limited Scalability	Limited Scalability	Process complexity IR-drop	Technology Maturity

Table R4. Benchmarking of SA hardware accelerator technologies. *Benchmarking with various combinatorial problems solved by hardware accelerators of different technology. 2D memtransistors demonstrate highest projected scalability at lowest instantaneous power consumption.*

3. The manuscript highlights the performance advantage of the hardware accelerator over an exhaustive brute-force search. However, a comparison with a software-based SA implementation running on a general-purpose processor would be highly informative. It is recommended to supplement the analysis, for instance by adding a cumulative distribution curve for a software SA baseline in Figure 5f, comparing metrics like the median number of iterations to convergence and failure rates on identical problem instances.

We agree that comparison of the hardware-based annealing accelerator with a software-based SA implementation running on a general-purpose processor would be informative. To that end, we analyze the performance of our hardware-realistic simulation of the annealing accelerator on the same 4x4 drone system of size 10x10 with three obstacles that was evaluated in hardware. The initial positions of all drones were kept constant across hardware and software initializations, but the hardware-realistic simulation utilized pseudo-random bits from the python 'random' module rather than bits generated by our TRNG module. We assess the convergence behavior of this system in software by evaluating 100 different initializations from the same starting position with an identical annealing schedule. The system overlap as a function of iteration is shown for all 100 random initializations in **Fig. R14a**. We found that 66 out of the 100 initializations converged to an ideal solution which corresponds to a failure rate of 34%. **Fig. R14b** shows a cumulative distribution plot of the number of iterations required to reach an optimal solution for the 100 random initializations. Here the median number of iterations to convergence was 113.5 amongst those iterations that converged to an ideal solution. These results and corresponding discussion have been added to the revised manuscript.

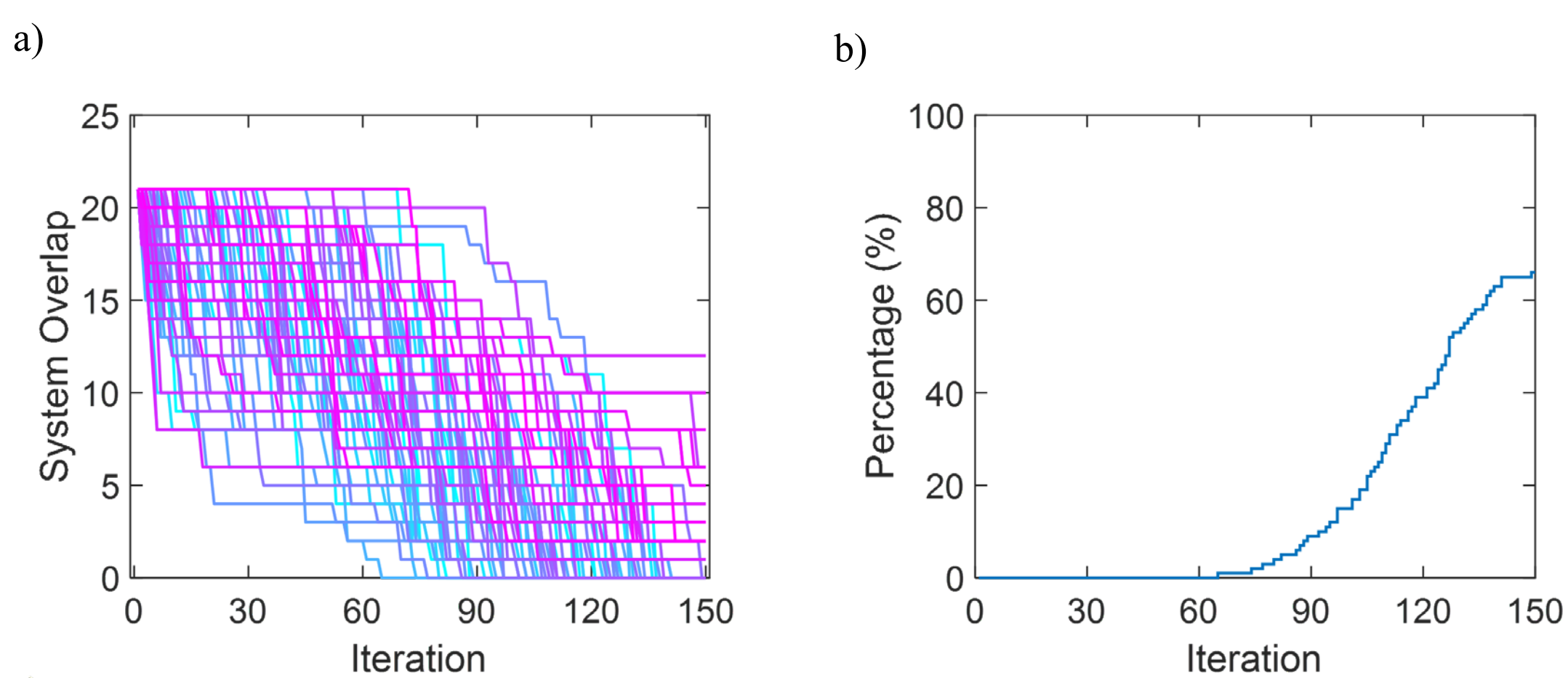

Fig. R14. 100 initializations of experimental drone system. (a) *System overlap as a function of iteration for 100 SA initializations.* ***(b)*** *Cumulative distribution plot illustrating the percentage of converged initializations at each iteration.*

4. Fig. 5f shows that a small subset of initializations failed to reach the global optimum. An analysis of the underlying reasons for these failures would be insightful. Furthermore, beyond the TRNG, it would be valuable to discuss whether non-idealities in the 2D devices (e.g., variability, fluctuations) may influence convergence behavior, especially as the system scales in the number of drones or grid size. Do these hardware characteristics help escape local minima or impede convergence?

We thank the reviewer for this suggestion. **Fig. 5f** of the submitted manuscript shows a cumulative distribution plot of the number of iterations required to reach an optimal solution for 50 random initializations of a four 4x4 drone system of size 10x10 with three obstacles. The obstacle locations were randomly assigned during each of the 50 SA initializations. In this experiment, 46 out of 50 initializations converged within the total number of iterations allowed by the prescribed annealing schedule. **Supplementary Video 2** in the revised manuscript shows the evolution of the generated solution for 10 of the random initializations. In four random initializations, the algorithm concluded its annealing schedule of 360 iterations without reaching one of the global minima. **Supplementary Video 3** in the revised manuscript shows the system evolution over all iterations for the four initializations where the SA algorithm did not converge to an optimal solution. In the first two cases, the system was close to an ideal solution. Here, extended iterations at the lowest temperature would eventually allow the system to converge to an ideal solution. In the third and fourth cases, the system is clearly in a local minimum and must traverse high energy states to reach an optimal solution. Here, a repeated annealing schedule that includes higher temperatures would be required for the system to reach an optimal solution. As discussed in our response to comment 1, experimental variability in the hardware modules did not alter the convergence behavior of the annealing accelerator. This discussion has been added to the revised manuscript.

5. The acceptance module relies on programmable memtransistors whose retention was shown for 200 seconds (Extended Data Fig. 8). Since the annealing process involves 5 times temperature changes and 50 on-chip experiments, it is important to evaluate endurance through repeated programming cycles (e.g., >250 cycles) and measure retention properties after endurance testing.

The reviewer has raised an important point regarding the stability and retentiveness of the programmed resistive states through repeated programming cycles. Since our acceptance module relies on repeated programming of the thresholding circuit, it is critical to verify that endurance cycling does not degrade the device characteristics. To address this, endurance of a representative MoS_2 memtransistor was evaluated. We define a single endurance cycle as the sequential pulsing of −10 V and 10 V to the back-gate of the memtransistor for a time duration of $1\mu s$. These positive and negative voltage pulses program the memtransistor into a low-resistance state (LRS) and a

high-resistance State (HRS), respectively. This procedure was repeated for 10^5 cycles. The LRS and HRS were recorded at a back-gate voltage of -2 V and a drain volage of 1 V after 10, 100, 1,000, and 10,000 programming cycles.

Fig. R15a shows the evolution of the LRS and HRS as a function of the cycle count. Both states remain well separated across the full endurance experiment, demonstrating stable programmability. **Fig. R15b** presents the retention characteristics at four programmed analog memory states measured after the prior endurance experiment. The retention of each analog memory state was monitored over 200 s per state following the endurance experiment. These results confirm that the stability and retention of programmed memory states are not significantly compromised by 10^5 programming cycles. These results and associated discussion have been added to the revised manuscript.

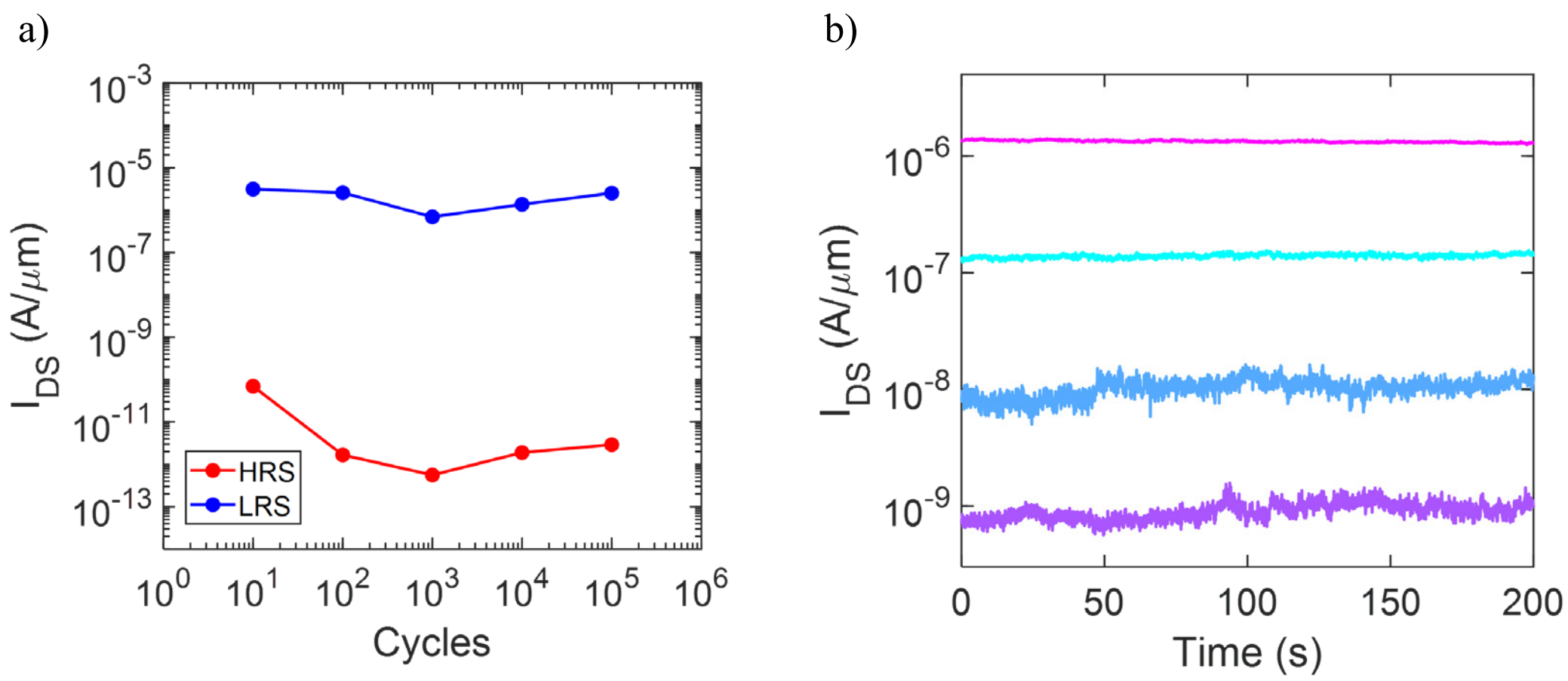

Fig. R15. Endurance and post-endurance retention of programmed states in MoS_2 Memtransistors. (a) *Evolution of low-resistance state (LRS) and a high-resistance State (HRS) over 10^5 programming cycles.* ***(b)*** *Source-to-drain current of a representative MoS_2 memtransistor measured at a back-gate voltage of 0 V for 200 s after it was programmed into four different conductance states. Retention of memory states was measured after the endurance experiment.*

6. The current work focuses on a specific coverage-overlap model with an energy functon(H) implemented via relatively simple logic operations. The authors should comment in the Conclusion or Discussion on the generalizability of this architecture to other optimization problems (e.g., TSP), including any necessary modifications at the hardware or algorithm level.

The constrained spatial optimization problem that we address can be broadly represented as a 2D packing and placement task. The formulation of this problem is similar in structure to very-large-

scale integration (VLSI) floorplan design tasks where fixed-footprint objects must be positioned on a grid without overlap while respecting forbidden regions. The approach utilized in this work could potentially be generalized to other optimization problems set in a discrete spatial domain with arbitrary constraints. For example, the eight queens problem could be solved by initializing agents of the shape described by the allowable movement regions of a queen chess piece. By minimizing overlap between agents, the system would potentially converge to a permissible solution. Nonetheless, the exact circuit implementation of our work cannot directly be generalized to optimization problems such as graph coloring, max-cut, sudoku, or the traveling salesman problem. However, only the energy computation and summation modules of the hardware-based annealing accelerator would require modification to create a hardware-based annealing accelerator capable of solving these problems. The remaining TRNG and acceptance modules would not require modification as they perform general purpose annealing operations. This discussion has been added to the revised manuscript.

7. There are some labeling issues in the figures. For example, the y-axis in Fig. 2c is missing partial labels (numbers 2 and 3), and the y-axis label in Fig. 5d should be "Number of Drones".

We thank the reviewer for bringing these labeling issues to our attention. They have been fixed in the revised version of the manuscript.

Reviewer #3 (Remarks to the Author):

The title of this paper initially led me to believe that the authors had developed a complete system to solve the constraint satisfaction problem using quantum annealing. However, it appears that they merely simulated a potential hardware implementation without combining a few basic circuits they report into a system. The simulations are relatively straightforward, and the experimental aspect feels incomplete. Consequently, I do not think this work is ready for publication in Nature Communications. So, my recommendation is to reject.

We would like to note that 'quantum annealing' was not listed in the title or anywhere in the main text of the submitted manuscript. Therefore, it is somewhat unclear how this misconception arose. Simulated annealing (SA) explores an energy landscape using a process inspired by physical annealing where movements are evaluated with a temperature-dependent probability that helps the system escape local minima. In comparison, quantum annealing (QA) allows tunneling through barriers in the energy landscape. While SA relies on a temperature schedule to guide the search, QA encodes the problem into a Hamiltonian whose ground state corresponds to the optimal solution [27]. We have added discussion in the introduction section regarding quantum annealing

and explicitly compared it to the simulated annealing approach that we utilize to solve constraint satisfaction problems. Furthermore, we would like to clarify that the scope of our hardware implementation extends beyond the circuit demonstrations shown in **Fig. 2** of the submitted version of the manuscript. The hardware-based simulated annealing accelerator discussed in the submitted version of the manuscript was utilized to find the solution to a four-drone, three-obstacle system in a 10x10 spatial domain. Experimental results shown in **Fig. 4** of the submitted version of the manuscript illustrate the progression of the hardware-generated solution throughout the annealing procedure. Hardware-realistic simulations are then introduced to evaluate the performance of our formulation of the SA algorithm in larger drone systems.

References

[1] R. Serrano *et al.*, "A fully digital true random number generator with entropy source based in frequency collapse," *IEEE Access,* vol. 9, pp. 105748–105755, 2021.

[2] R. Serrano *et al.*, "A robust and healthy against PVT variations TRNG based on frequency collapse," *IEEE Access,* vol. 10, pp. 41852–41862, 2022.

[3] Y. Lu *et al.*, "Jitter-quantizing-based TRNG robust against PVT variations," *IEEE Access,* vol. 8, pp. 108482–108490, 2020.

[4] S. Tao and E. Dubrova, "TVL-TRNG: Sub-microwatt true random number generator exploiting metastability in ternary valued latches," in *2017 IEEE 47th International Symposium on Multiple-Valued Logic (ISMVL)*, 2017: IEEE, pp. 130–135.

[5] D. Akinwande *et al.*, "Graphene and two-dimensional materials for silicon technology," *Nature,* vol. 573, no. 7775, pp. 507–518, 2019.

[6] S. Das *et al.*, "Transistors based on two-dimensional materials for future integrated circuits," *Nature Electronics,* vol. 4, no. 11, pp. 786–799, 2021.

[7] K. P. O'Brien *et al.*, "Process integration and future outlook of 2D transistors," *nature communications,* vol. 14, no. 1, p. 6400, 2023.

[8] S. Ghosh *et al.*, "Monolithic and heterogeneous three-dimensional integration of two-dimensional materials with high-density vias," *Nature Electronics,* vol. 7, no. 10, pp. 892–903, 2024.

[9] T. F. Schranghamer *et al.*, "Large-scale crossbar arrays based on three-terminal MoS2 memtransistors," *Nature Communications,* vol. 16, no. 1, p. 9518, 2025.

[10] M. U. K. Sadaf *et al.*, "Enabling static random-access memory cell scaling with monolithic 3D integration of 2D field-effect transistors," *Nature communications,* vol. 16, no. 1, p. 4879, 2025.

[11] D. Jayachandran *et al.*, "Three-dimensional integration of two-dimensional field-effect transistors," *Nature,* vol. 625, no. 7994, pp. 276–281, 2024.

[12] H. Ravichandran *et al.*, "A peripheral-free true random number generator based on integrated circuits enabled by atomically thin two-dimensional materials," *ACS nano,* vol. 17, no. 17, pp. 16817–16826, 2023.

[13] S. Ghosh *et al.*, "A complementary two-dimensional material-based one instruction set computer," *Nature,* vol. 642, no. 8067, pp. 327–335, 2025.

[14] C. Cook, H. Zhao, T. Sato, M. Hiromoto, and S. X.-D. Tan, "GPU-based ising computing for solving max-cut combinatorial optimization problems," *Integration,* vol. 69, pp. 335–344, 2019.

[15] H. Kagawa *et al.*, "Fully-pipelined architecture for simulated annealing-based QUBO solver on the FPGA," in *2020 Eighth International Symposium on Computing and Networking (CANDAR)*, 2020: IEEE, pp. 39–48.

[16] M. Yamaoka, C. Yoshimura, M. Hayashi, T. Okuyama, H. Aoki, and H. Mizuno, "24.3 20k-spin Ising chip for combinational optimization problem with CMOS annealing," in *2015 IEEE International Solid-State Circuits Conference-(ISSCC) Digest of Technical Papers*, 2015: IEEE, pp. 1–3.

[17] J. H. Shin, Y. J. Jeong, M. A. Zidan, Q. Wang, and W. D. Lu, "Hardware acceleration of simulated annealing of spin glass by RRAM crossbar array," in *2018 IEEE International Electron Devices Meeting (IEDM)*, 2018: IEEE, pp. 3.3. 1–3.3. 4.

[18] D. Shin, N. Onizawa, W. J. Gross, and T. Hanyu, "Memory-efficient FPGA implementation of stochastic simulated annealing," *IEEE Journal on Emerging and Selected Topics in Circuits and Systems,* vol. 13, no. 1, pp. 108–118, 2023.

[19] P. Mannocci *et al.*, "In-memory computing with emerging memory devices: Status and outlook," *APL Machine Learning,* vol. 1, no. 1, 2023.

[20] N. Lepri, A. Glukhov, P. Mannocci, M. Porzani, and D. Ielmini, "Compact modeling and mitigation of parasitics in crosspoint accelerators of neural networks," *IEEE Transactions on Electron Devices,* vol. 71, no. 3, pp. 1900–1906, 2024.

[21] J. Zhu *et al.*, "Low-thermal-budget synthesis of monolayer molybdenum disulfide for silicon back-end-of-line integration on a 200 mm platform," *Nature Nanotechnology,* vol. 18, no. 5, pp. 456–463, 2023.

[22] H.-L. Tang *et al.*, "Demonstration of Conformal MoS 2 on High-Aspect-Ratio Structures up to 40: 1 and Exploration of Manufacturability in a 300mm Fab for 3D NAND applications," in *2025 IEEE International Memory Workshop (IMW)*, 2025: IEEE, pp. 1–4.

[23] C. Wu *et al.*, "High performance 22/20nm FinFET CMOS devices with advanced high-K/metal gate scheme," in *2010 International Electron Devices Meeting*, 2010: IEEE, pp. 27.1. 1–27.1. 4.

[24] "Tech Power Up - NVIDIA Tesla K40c." https://www.techpowerup.com/gpu-specs/tesla-k40c.c2505 (accessed 12/4/2025, 2025).

[25] A. Sebastian, S. Das, and S. Das, "An Annealing Accelerator for Ising Spin Systems Based on In-Memory Complementary 2D FETs," *Advanced Materials,* vol. 34, no. 4, p. 2107076, 2022.

[26] T. F. Schranghamer *et al.*, "Radiation resilient two-dimensional electronics," *ACS Applied Materials & Interfaces,* vol. 15, no. 22, pp. 26946–26959, 2023.

[27] N. Odelius and I. Reinholdsson, "Comparing Quantum Annealing and Simulated Annealing when Solving the Graph Coloring Problem," ed, 2023.

Reviewer #1 (Remarks to the Author):

The authors have significantly expanded the results and have made substantial improvements to the manuscript overall. Most of the responses seem to be satisfactory, while I still remain one concern:

In the response to comment 5, the authors indicate that the TRNG speed is 200 Hz. This suggests that the random number generation rate of the 2D memtransistors in this work could become a bottleneck for hardware acceleration.

Existing works have shown that RRAM could be integrated in monolithic 3D technology and this technology supports certain extent of logic capabilities. Moreover, RRAM-based TRNGs have been reported to achieve bandwidths at the Mbps level. Therefore, it is important for the authors to clarify the reason of choosing 2D memtrasistors over RRAM, in light of the 3D integration capability and the fast TRNG of RRAM.

It is understandable that 2D memtransistor technology is still at an early stage. It would be valuable if the authors could discuss the operating speed comparison on RRAM and this work. An explanation on the potential advantages of 2D memtransistors would also be helpful.

We thank the reviewer for their positive assessment of the revised manuscript and for raising an important system-level concern regarding throughput. We agree that a true random number generation rate that is intrinsically limited to the few-hundred Hz regime would become a bottleneck in many hardware acceleration applications. In our previously reported results, the TRNG output is sampled once every 5 ms (200 samples/s). However, we would like to clarify that this 200 Hz reflects the sampling rate used during measurements rather than a fundamental limit of the entropy source. **Fig. R1** shows a stem plot of 1,000 random bits sampled at a rate of 10 kHz. This measurement rate corresponds to a 50× improvement relative to the present demonstration. Further improvements in TRNG speed are achievable at the circuit level by operating multiple TRNG modules concurrently or at the device level with defect/interface engineering that aims to accelerate the underlying stochastic dynamics at the semiconductor–dielectric interface [1-6]. While extensive TRNG optimization is beyond the scope of this manuscript, we view it as an active research direction.

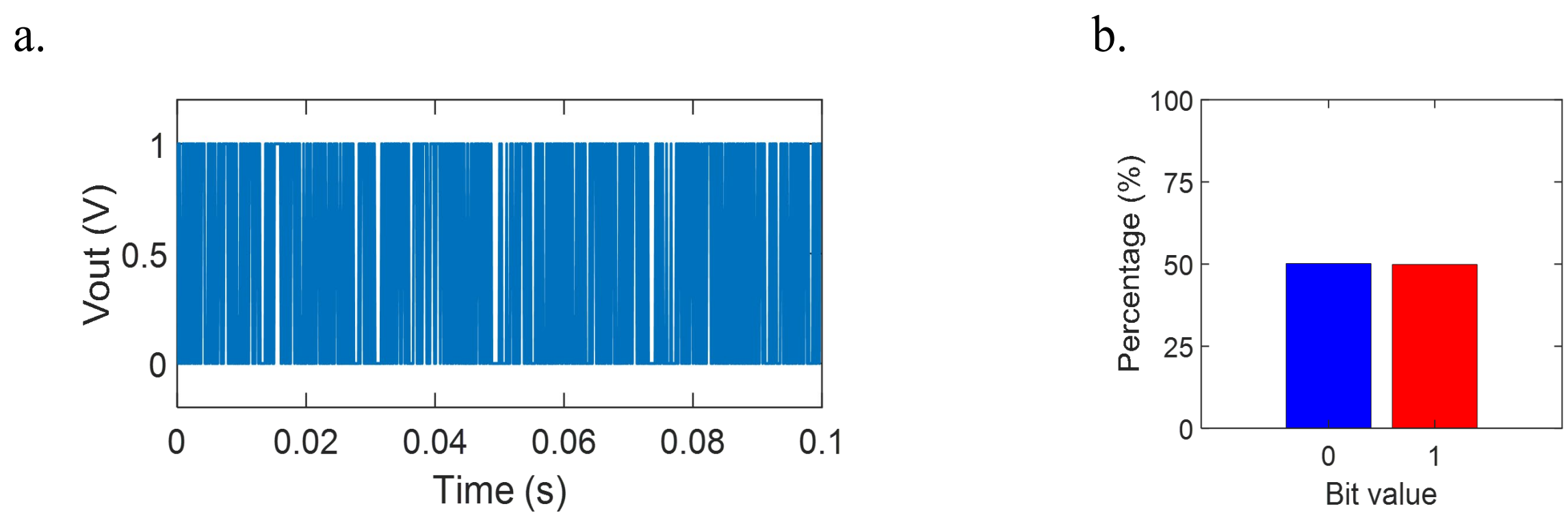

Figure R1: Random bits sampled at 10 kHz. (a) *Representative sampled of a generated random bitstream comprising 1,000 random bits sampled at 10 kHz.* ***(b)*** *Histogram of 100,000 generated bits showing an approximately equal distribution of 0's and 1's.*

Additionally, we agree with the reviewer that discussion justifying the use of 2D memtransistor-based TRNGs rather than RRAM-based TRNGs should be included in the manuscript. While RRAM-based TRNGs have been reported with higher raw bit generation bandwidths (often kbps–Mbps depending on the scheme and readout [7-9], our selection of 2D memtransistors for this application is motivated by the need for system level co-integration of circuits with varying functionality. Specifically, beyond random number generation, the proposed annealing accelerator also requires circuits that are capable of logic computation, summation, and tunable acceptance. Our 2D memtransistor-based platform enables random number generation, CMOS logic, and programmable acceptance within a single materials system, whereas RRAM-based approaches would typically require separate CMOS/peripheral circuitry for Boolean evaluation and acceptance control. Although non-volatile and reconfigurable logic gates based on RRAMs have been demonstrated [10, 11], each RRAM cell requires a dedicated transistor for programming. Without this transistor, it would not be possible to properly regulate the current needed to induce the transition to the desired memory state. This requirement entails additional metal routing, resulting in increased pitch and parasitic effects. In addition, RRAM/CBRAM operation involves larger currents and greater sensitivity to parasitic voltage drops (e.g., in scaled arrays/crossbars), while our 2D CMOS circuits operate at much lower currents [12]. The annealing acceptance criterion is also naturally realized via programmable threshold tuning (e.g., a programmable inverter threshold), which maps directly onto memtransistor behavior. Implementing an equivalent compact, tunable acceptance element with RRAM generally introduces additional mixed-signal/peripheral overhead, which can erode the benefit of high raw TRNG bandwidth when targeting a tightly integrated, low-overhead annealing loop. In conclusion, although RRAM-based TRNGs are well suited for applications that demand extremely high random-bit throughput, other applications that require co-integration of low power circuits with varying functionality inherently favor the use of 2D memtransistor devices.

The ‘Introduction’ section of the revised manuscript was modified to include the following commentary that further explains the choice to utilize 2D memtransistors rather than RRAM in our hardware-based annealing accelerator. (Pg. 5-6): It is also important to note that the SA algorithm requires a source of random numbers that are used for the generation and probabilistic acceptance of proposed solutions. True random number generators based on 2D memtransistors and RRAMs [13, 14] are highly energy- and area- efficient in comparison to true random number generators based on Si CMOS that exploit frequency collapse in multimodal ring oscillators [15, 16], jitter in ring oscillators [17], or metastability in latches [18]. Although RRAM and CBRAM are mature technologies, they lack intrinsic logic capabilities. Non-volatile and reconfigurable logic gates based on RRAMs have been demonstrated[10, 11], but each RRAM cell requires a dedicated transistor for programming. This dedicated transistor necessitates additional metal routing, resulting in increased pitch and parasitic effects. 2D memtransistors, on the other hand, are still far from industry level standards, however they will be able to vertically integrate random number generation, memory, and logic, an essential requirement for this class of applications.

The ‘Drone placement in a constrained environment’ section of the revised manuscript was modified to include the following commentary that provides additional context explaining the TRNG sampling time and compares the speed of 2D memtransistor-based TRNGs and RRAM-based TRNGs. (Pg. 23): ‘Additionally, the 5 ms/bit TRNG module sampling time does not reflect the fundamental sampling limit of the entropy source. In comparison, TRNGs based on RRAM technology have been reported with higher raw bit generation bandwidths in the range of kbps–Mbps depending on the scheme and readout [19]. Higher bitrates for TRNGs based on MoS_2 memtransistors are achievable at the circuit level by operating multiple TRNG modules concurrently or at the device level with defect/interface engineering that aims to accelerate the underlying stochastic dynamics at the semiconductor–dielectric interface [1-6].

Reviewer #2 (Remarks to the Author):

I appreciate the efforts that the authors have made to address the previous comments, and the revisions have improved the manuscript. However, there are still several critical concerns that need to be addressed:

We would like to thank the reviewer for providing constructive feedback that has enhanced the quality and clarity of the manuscript. We hope that the following commentary and associated manuscript revisions address the remaining concerns.

1) The authors should explicitly specify the exact locations where revisions have been incorporated in the revised manuscript. Vague statements such as "The above comparisons have been added to the revised manuscript" are insufficient, as they place an undue burden on reviewers to find the changes.

We understand the concern raised by the reviewer and apologize for failing to specify the exact location of revisions to the revised manuscript in our prior submission. Within this rebuttal document, we have explicitly stated the location of the added commentary and figures within the revised manuscript.

2) While the authors provide images of individual circuit components (e.g., TRNG and inverters), there is a lack of experimental evidence for the integrated system. Specifically, a physical photograph of the complete annealing accelerator (Fig. 1a) is essential to validate the hardware implementation. This should include visible interconnections between modules (e.g., TRNG, energy computation, summation, and acceptance units) to demonstrate practical integration and scalability.

The reviewer raises a valid concern. We would like to note that all measurements were performed modularly, i.e., module by module. An image of the two chips utilized for experimentation inside our Lake Shore CRX-VF probe station is shown in **Fig. R2**. The left chip contains the true random number generator and acceptance modules while the right chip contains the energy computation module and the transistor utilized for the summation module. The capacitor utilized for the summation module was connected externally using a breadboard. We chose to perform the experiments in this manner because the flexible setup allowed us to measure different combinations of intermediate circuit nodes more readily than what would be possible using a more integrated system. Moreover, the usage of our Keysight B1500A parameter analyzer allowed us to measure the low currents that arose in the 2D transistor-based circuits and accurately estimate the power consumption throughout each circuit module during annealing experiments.

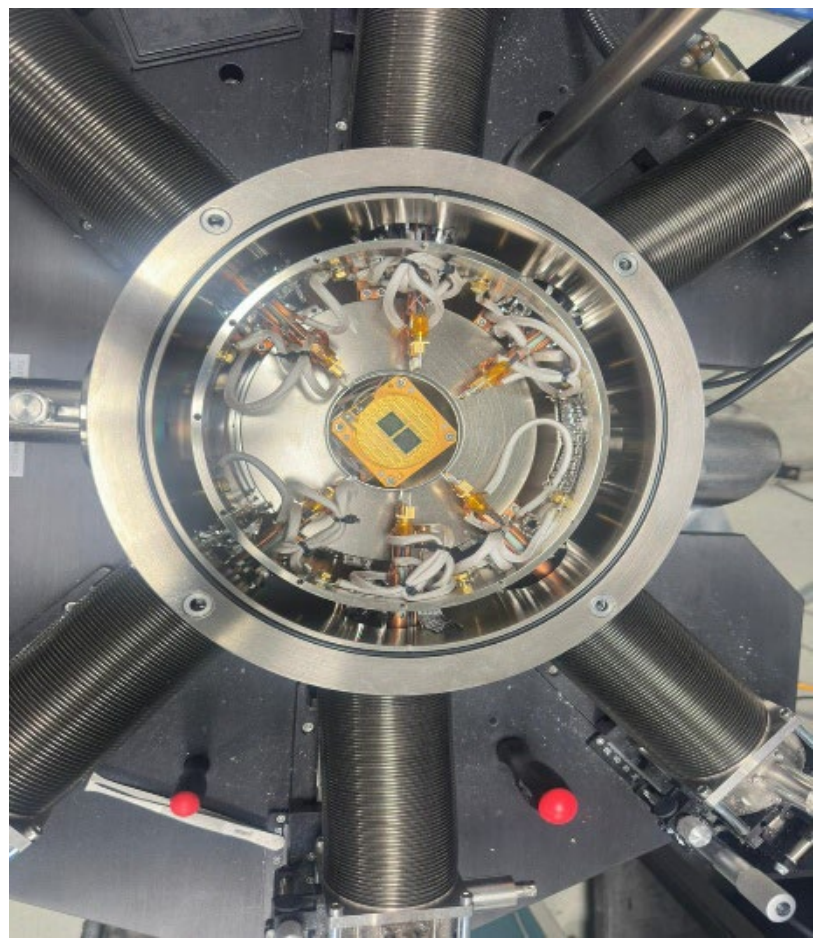

Figure R2: Image of experimental setup: *The left chip contains the true random number generator and acceptance modules while the right chip contains the energy computation module and the transistor utilized for the summation module. Both chips are shown inside the Lake Shore CRX-VF probe station that was utilized for annealing experiments. The capacitor was connected externally through a breadboard.*

3) The term "hardware-realistic simulation" is used ambiguously throughout the manuscript. The authors must clearly delineate which results are derived from experimental measurements and which are based on simulations. Explicit evidence should be provided.

The reviewer raises a valid concern regarding the ambiguous usage of 'hardware-realistic simulation' throughout the manuscript. We decided instead to utilize 'simulation of the annealing accelerator' and briefly describe it in the '2D material-based annealing accelerator' section as follows (Pg. 9): A simulation of the annealing accelerator was created to model the operations performed by each circuit module given an arbitrary system configuration and annealing schedule. More information on the simulation of the annealing accelerator can be found in the **methods** section.

As stated, this brief description is followed by a more detailed description in the methods section. We have renamed the 'simulation details' methods section to 'simulation of annealing accelerator' and added the following commentary (Pg. 39-40): *Simulation of annealing accelerator:* The SA accelerator simulation models the operations performed by each circuit module. First, pseudo-random numbers dictating the moving drone and its movement direction are generated to replicate the TRNG module. Next, logical Equations 1-3 are used to perform the energy computation. Subsequently, Equation 4 is used to represent the summation module. Finally, a sigmoid function is used to mimic the acceptance criterion implemented by the acceptance module. The output of the acceptance module, V_{AM} is modeled using Equation 6 below:

$$V_{AM} = \frac{V_{DD}}{1+e^{-a(V_{IN}-V_{SW})}} \quad [6]$$

Here, V_{DD} is the supply voltage, V_{IN} is input voltage, V_{SW} is the switching threshold of the inverter, and a is a fitting parameter that is modified to emulate the approximate gain of a three-stage inverter.

We have also ensured that all results presented in the revised manuscript are accompanied by a statement describing either that they were experimental results or results based on simulation. Statements were added throughout the revised manuscript that explicitly delineate the source of presented results in the following locations:

- Pg. 9 - This simulation was used to illustrate the progression of solution quality throughout the annealing process for the 10×10 system with 4 agents of size 4×4. The annealing schedule comprises 120 iterations per temperature at five descending temperatures for a total of 600 iterations. **Fig. 1c-d** show the occupancy map of the agents at iterations 72 and 254, respectively, while **Fig. 1e** shows the final optimized state of the system that was reached at iteration 395. **Fig. 1f** depicts the total system overlap as a function of iterations with progressive reductions in temperature illustrated by the color of the underlying pane. Arrows 2,3, and 4 denoting the system energy corresponding to iterations 72, 254 and 395, respectively. Clearly, iterations 72 and 254 correspond to local minima where the agents are required to move across high overlap solutions to eventually reach an optimal zero overlap solution.
- Pg. 16 - Stem plots depicting the experimental output of the environment calculation submodule are shown for each movement direction in **Fig. 3g**.
- Pg. 17 - Stem plots of H_{Prior} and H_{New} depicting the experimental output of the overlap calculation submodules are shown for each movement direction in **Fig. 3h** and **Fig. 3i**, respectively.
- Pg. 18 - The annealing accelerator simulation was utilized to quantify the error introduced by each hardware module. Ideal values for the output of the energy computation module, the summation module, and the acceptance module were determined through simulation utilizing an identical initialization of the four-drone, three-obstacle 10x10 system and the experimentally realized string of TRNG bits.
- Pg. 25 - Next, the scale of the spatial optimization problem was varied to evaluate the performance of the SA accelerator simulation in sequentially larger solution spaces.

Note that results in the 'Accelerator modules created with two-dimensional transistors' section should be reasonably inferred as experimental results and as such we did not modify this section. For reference, this section includes transistor transfer characteristics, sample experimental outputs from the TRNG, summation, and acceptance modules, and experimental demonstration of the universal logic gates that comprise the energy computation module. We thank the reviewer for this comment and we expect that these changes will increase the clarity of the manuscript for future readers.

4) The claimed 1800-fold acceleration over brute-force search requires clearer context. The authors should clarify whether this improvement stems primarily from the simulated annealing algorithm itself or the hardware implementation. If the acceleration is algorithmic, the novelty of the hardware implementation must be emphasized without overstating its role.

We would like to reiterate our prior statement that the main contribution of our work is the hardware acceleration of the simulated annealing algorithm using complementary metal-oxide-semiconductor (CMOS) circuits based on 2D semiconductor materials. The claimed 1800-fold acceleration over brute force search can be attributed to the annealing algorithm. Nonetheless, our annealing accelerator executes the simulated annealing algorithm operations to realize this 1800-fold acceleration in a low-power hardware system using only 54 transistors, one capacitor, and an external controller. We agree with the reviewer that the role of both the SA algorithm and the hardware implementation of the annealing accelerator should be clearly articulated.

We have modified the following statement in the abstract to emphasize that the hardware is utilizing the SA algorithm to achieve the 1800-fold search acceleration. The energy consumption claim was also added here to specify a benefit of the hardware implementation. (Pg. 2): For a representative drone placement task, our hardware implementation of the SA algorithm consumes 76 nJ of energy per iteration and achieves an 1800-fold search acceleration relative to exhaustive brute-force exploration of the solution space.

To provide further clarity, we have also modified the following statements in the 'introduction' section regarding search acceleration to emphasize the distinction between the contributions made by the algorithm and the hardware implementation (Pg. 6): In a 10×10 discrete spatial domain with three obstacles and four drones, our annealing accelerator converged to a solution at a minimal energy expenditure of approximately 76 nJ per iteration while utilizing the SA algorithm to provide a search acceleration of 1800× relative to a brute-force search method.

5) The median convergence iteration count for software-based SA (113.5) differs from the hardware-reported value. The authors should explain this discrepancy.

We thank the reviewer for this suggestion. We would like to provide some additional information to help explain this discrepancy. The reported median convergence iteration of 113.5 represents the median convergence iteration out of the 66 simulated SA initializations that converged. For this subset of SA initializations, the mean and standard deviation of the convergence iteration were 112.98 iterations and 18.54 iterations, respectively. The hardware-based annealing accelerator converged at iteration 134, which is slightly slower than the average software initialization by approximately one standard deviation. This discrepancy in convergence speed can be attributed to the inherent variability of the simulated annealing algorithm.

We have included the following additional commentary within the 'Simulation of annealing accelerator' section (Pg. 25): For the subset of simulation initializations that converged, the mean

and median number of iterations required to reach an optimal solution were 112.98 and 113.5, respectively, with a standard deviation of 18.54 iterations. The hardware-based annealing accelerator converged at iteration 134, which is slightly slower than the average software initialization by approximately one standard deviation. This discrepancy in convergence speed can be attributed to the inherent variability of the simulated annealing algorithm.

6) Although hardware errors are shown to be negligible in small-scale experiments, their cumulative effect in larger systems (e.g., higher-dimensional grids or more drones) must be evaluated. The authors should provide simulations or theoretical estimates of how error propagation could affect convergence reliability, along with potential mitigation strategies.

We thank the reviewer for this suggestion. We agree that the cumulative effect of hardware errors should be evaluated in larger systems. As demonstrated in the revised version of the manuscript (**Extended Data Fig. 14** and association discussion on pg. 18-20), the TRNG, energy computation, and acceptance modules exhibit negligible error in small-scale experiments. We do not expect the error in the TRNG and energy computation modules to increase in larger systems because these circuits are entirely digital. While the acceptance module receives analog inputs, the gain of the three-stage inverter is sufficiently large that at a given switching threshold, the output of this module should exhibit little-to-no error as the system size increases. As previously discussed, the analog summation module is the largest source of computation error in our proposed annealing accelerator. For that reason, we have augmented our prior results with a detailed analysis describing the operating principles and intrinsic limitations of the summation circuit. The SA accelerator simulation was utilized to analyze the impact of experimental summation error on SA convergence in systems ranging from size 10×10 to 360×360. Finally, we suggest strategies that can be used to mitigate detrimental effects introduced by error in the summation circuit.

The summation circuit, shown for reference in **Fig. R3a**, consists of a single n-type MoS_2 transistor and one capacitor. The transistor operates as a switch, controlling the charging and discharging path of the capacitor. When a positive back-gate voltage (V_{BG}) is applied biasing the transistor in its ON-state, the capacitor will charge or discharge depending on the polarity and magnitude of the voltage applied at the input terminal (V_{IN}). Application of negative V_{BG} biases the transistor in its off-state, thereby preventing undesired capacitor discharge over time. The summation is performed by sequentially applying H_{New} and $-H_{Prior}$ bit streams to the input terminal of the summation circuit. Non-zero H_{New} and $-H_{Prior}$ inputs are accompanied by the simultaneous application of a positive V_{BG} to allow addition or subtraction while zero-valued inputs are accompanied by the simultaneous application of a negative V_{BG} to prevent discharge. During the addition operation, the input terminal acts as the drain of the transistor. As the capacitor charges, the effective gate-to-source voltage (V_{GS}) progressively decreases. Conversely, during subtraction, the application of a negative voltage causes the input terminal to act as the source, resulting in a fixed V_{GS}. As a

consequence, addition and subtraction do not occur symmetrically for fixed back-gate voltages and input voltage magnitudes.

To more thoroughly investigate the circuit dynamics and the error involved in the system, we have simulated the summation circuit using LT Spice. Here, three time phases are considered corresponding to the addition of H_{New} (t_1), the subtraction of H_{Prior} (t_2), and a final capacitor discharge that resets the circuit (t_3). During the addition phase, the input terminal is driven by a pulsed waveform with a peak voltage of 5 V, which charges the capacitor. During each 5 V input pulse, a simultaneous pulse with a peak voltage of 5V is applied to the back-gate terminal. Between pulses, both the drain and back-gate voltages are set to 0 V, thereby turning the transistor off and

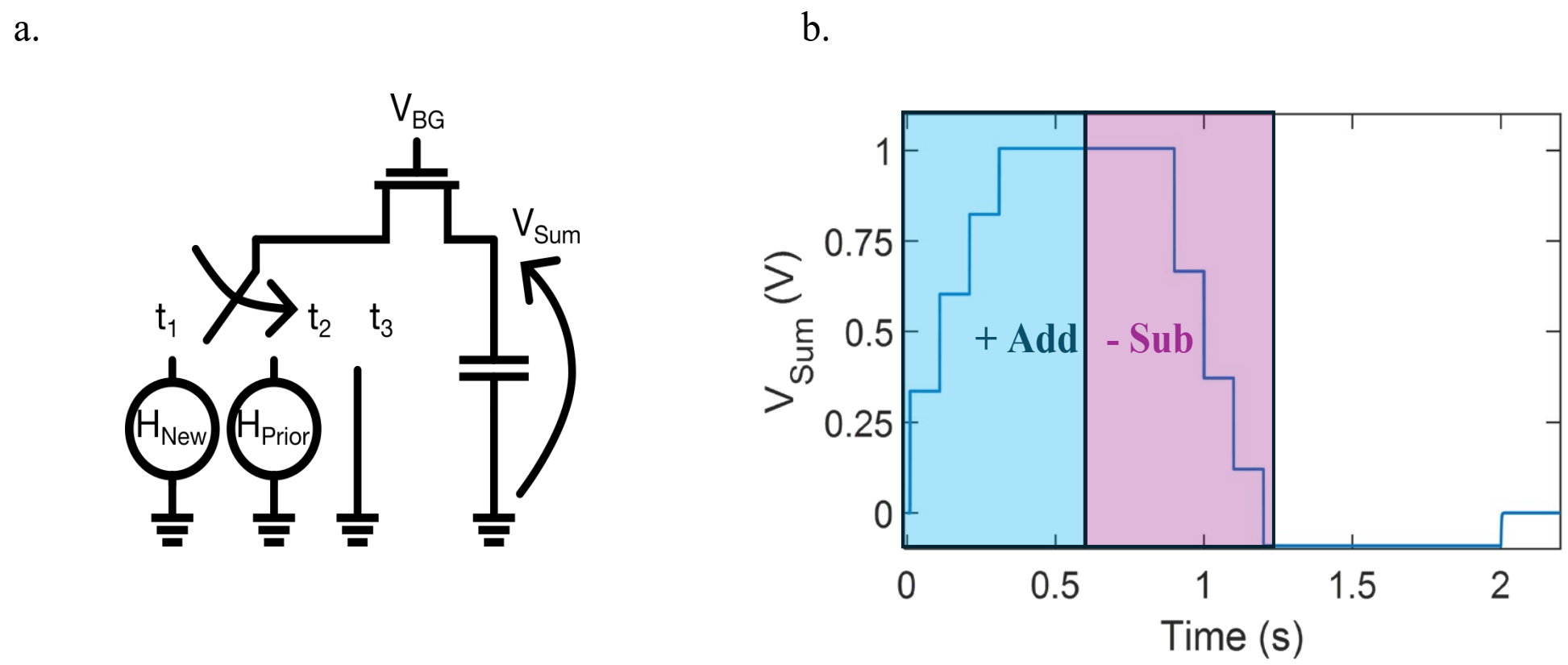

Figure R3: LT Spice simulation of the summation circuit. (a) *Circuit schematic of the summation module comprising a single n-type MoS_2 transistor and one capacitor. Three time phases are depicted corresponding to the addition of H_{New} (t_1), the subtraction of H_{Prior} (t_2), and a final capacitor discharge that resets the circuit (t_3).* ***(b)*** *Simulated output characteristics of the summation module which adds four pulses in the first 550ms and subtracts four pulses in the next 550ms. After summation, the capacitor is discharged to prevent error in subsequent summation operations.*

preventing any unintended discharge path through it. The overall pulse period is 10 ms, while the on-period T_{ON} of each pulse is 420 µs, corresponding to the effective addition time. The capacitance of the capacitor was set to $10\mu F$. As shown in **Fig. R3b**, the first 550 ms of the output waveform corresponds to four consecutive addition operations, resulting in an output voltage (V_{SUM}) of 1 V. During the addition phase, the transistor operates in saturation and therefore behaves as a current source. As a result, the initial additions produce larger voltage steps, while the incremental charge added to the capacitor decreases with each subsequent pulse. For the subtraction phase, the circuit employs pulse sequences at the gate and drain that are similar to those used for addition, but with a shorter T_{ON} of 300 µs. During subtraction, the transistor operates in

the triode region, behaving as a variable resistor that discharges the capacitor. As a result, the discharge process exhibits a variable time constant and occurs more rapidly than the charging process.

The pulse timings are chosen such that the resulting voltage reduction slightly undershoots the target subtraction value. For example, as shown around 900 ms in **Fig. R3b,** the expected output voltage after subtraction is 750 mV, yet the measured value is somewhat lower. This non-ideal summation behavior can influence the solution progression by causing the annealing accelerator to incorrectly accept or reject proposed solutions. For example, if the annealing temperature corresponds to an inverter switching threshold (V_{SW}) of 700 mV and the expected summation output (V_{SUM}) is 750 mV, the move should be rejected. However, if V_{SUM} drops below 700 mV, the move may be incorrectly accepted. The impact of summation error on the solution acceptance process largely depends on the ideal ΔH and the annealing temperature. In some cases, the ideal ΔH value is not close to the V_{SW} corresponding to the temperature prescribed by the annealing schedule. For example, if V_{SW} is 500 mV and the ideal ΔH is -250 mV, then significant summation error of 750 mV would be required to alter the acceptance decision. More generally, in cases where the ideal ΔH is negative, error is not likely to affect the decision process. However, if the ideal ΔH is positive, it is more likely that the ideal ΔH and V_{SW} are similar in magnitude. Consequently, this increases the possibility that a proposed solution is incorrectly accepted or rejected.

We utilize the SA accelerator simulation to further investigate the impact of experimental summation error on solution acceptance and SA convergence. We consider summation error in the SA accelerator simulation by adding a summation variability term to the ideal ΔH. In our previously discussed simulation results, the probability of acceptance ($P_{Acceptance}$) for a proposed solution was ~100% if $V_{SW} > \Delta H$ and ~0% if $V_{SW} \leq \Delta H$ following the thresholding function applied by the acceptance module. Considering finite summation error, $P_{Acceptance}$ is now a function of ΔH, the system temperature, and the summation variability term which is sampled from a normal distribution with a mean and standard deviation of μ_{Sum} and σ_{Sum}, respectively. **Fig. R4** shows $P_{Acceptance}$ as a function ΔH for five different temperatures (T_0 to T_4) with $\mu_{Sum} = -0.041\,V$ and $\sigma_{Sum} = 0.24\,V$. Note that $P_{Acceptance}$ at each ΔH and system temperature was determined numerically by collecting 10,000 samples from the variability distribution. $P_{Acceptance}$ represents the proportion of solutions that satisfy $V_{SW} > \Delta H + N(\mu_{Sum},\ \sigma_{Sum}^2)$. As expected, $P_{Acceptance}$ reduces with increasing ΔH and changing the system temperature causes a shift in the $P_{Acceptance}$ curve.

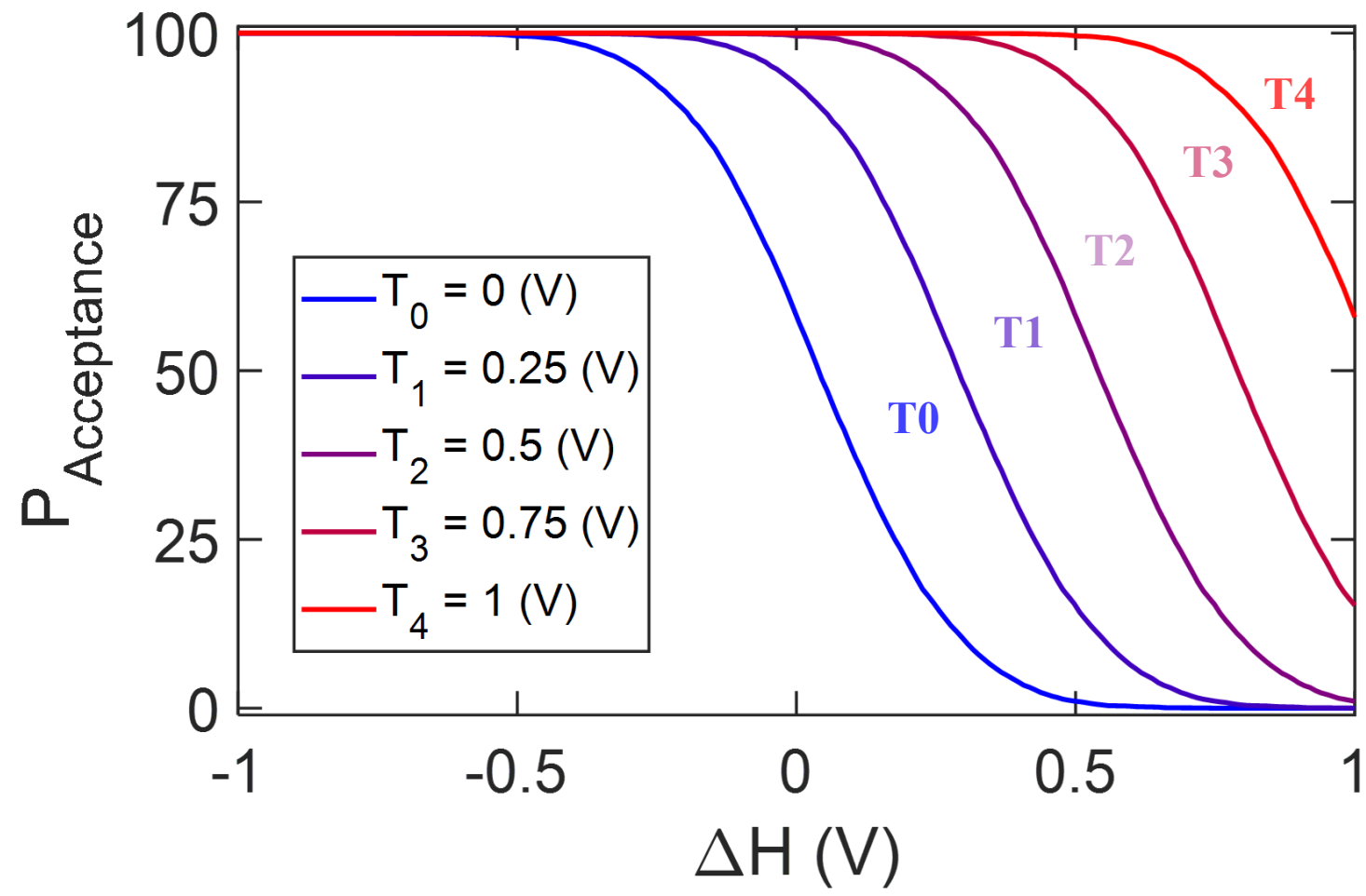

Figure R4: Probability of solution acceptance with summation error. *The probability of acceptance function of summation output at several annealing temperatures with a* σ_{Sum} *of 0.2 V. System temperatures* T_0*,* T_1*,* T_2*,* T_3*, and* T_4 *correspond to acceptance module switching thresholds of 0 V, 0.25 V, 0.5 V, 0.75 V, and 1 V, respectively.*

The modified SA accelerator simulation was evaluated in a variety of system sizes (10 ×10, 20 × 20, 40 × 40, 80 × 80, 160 × 160, and 320 × 320) with different magnitudes of σ_{Sum} ranging from 0 V to 1 V. The convergence rate, i.e., the proportion of SA initializations that converge to an ideal solution, was determined for a set of 100 SA initializations at each system size and σ_{Sum} combination. **Fig. R5a** shows the convergence rate as a function of σ_{Sum}. Clearly, the convergence rate decreases rapidly when σ_{Sum} surpasses ~ 0.3 V. Interestingly, there is modest variation in convergence rate across system sizes at a given σ_{Sum}. In our hardware summation circuit, the experimental σ_{Sum} was 0.24 V, indicating that the experimental summation error should not significantly degrade the SA convergence rate. However, increased levels of summation error beyond ~0.3 V effectively degrade the convergence rate for all system sizes. The system coverage, defined as the max possible system coverage divided by the highest realized system coverage, is a metric that can be utilized to evaluate the solution quality for a given SA initialization. The mean system coverage of the 100 SA initializations is shown for each system size and σ_{Sum} in **Fig. R5b**. Here, increasing σ_{Sum} has a modest effect on mean system coverage. Mean system coverage also exhibits a weak dependence on system scale, remaining above ~95% across all grid sizes. Although large summation error can prevent convergence to the global minimum energy, the best solution generated by the SA accelerator simulation is still likely to achieve high coverage.

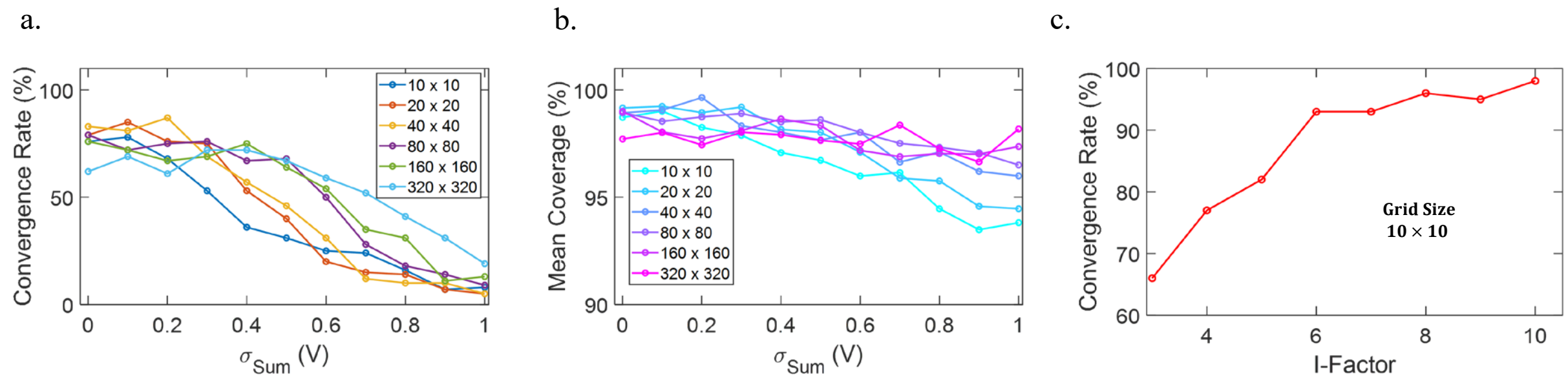

Figure R5: Effect of summation error on SA convergence. ***(a)*** *Convergence rate as a function of* σ_{Sum} *for 100 SA initializations at grid sizes of 10×10, 20×20, 40×40, 80×80, 160×160, and 320×320.* ***(b)*** *Mean coverage across 100 SA initializations as a function of* σ_{Sum}*. Coverage is above 92% for all system size and* σ_{Sum} *combinations.* ***(c)*** *Convergence rate as a function of the iterations per temperature factor (I) for 100 SA initializations of the 10×10 four drone, three obstacle system.*

Despite the minimal impact of experimental summation error on convergence rate, we believe that discussion of potential error mitigation techniques that may increase SA convergence rate would be a valuable addition to the revised manuscript. Here, we discuss an algorithmic error mitigation strategy before considering a circuit-level mitigation strategy. First, the annealing schedule can be modified to increase the convergence rate. For an $(A \times A)$ system, we set the number of iterations per temperature to $A * I$, where I is a multiplicative factor. The 10×10 four-drone system with three obstacles that was evaluated in hardware was utilized to investigate the impact of I on convergence rate. The initial positions of all drones were kept constant across hardware and software initializations. The convergence rate is shown as a function of I in **Fig. R3c**. The previously reported median convergence rate of 66% was obtained using $I = 3$, corresponding to 30 iterations per temperature. Clearly, the convergence rate increases monotonically with I, indicating that higher convergence rates can be achieved by increasing the number of iterations per temperature. However, this increase in convergence rate comes at the expense of additional computation time and resources. Also note that the set of temperatures prescribed by the annealing schedule is another critical SA parameter. We utilized five descending temperatures for this analysis. Different temperature sets would likely yield varying results. At the circuit level, a digital counter could be utilized as a more robust and scalable solution. However, a digital counter would require an increase in transistor count relative to the current summation circuit.

The 'Simulation of annealing accelerator' section of the revised manuscript was modified to include the following discussion regarding the operating principles and intrinsic limitations of the summation circuit, the modified SA accelerator simulation, and potential strategies that can be used to mitigate detrimental effects introduced by error in the summation circuit. (Pg. 32-35): Finally, we evaluate the impact of experimental error on SA convergence in systems ranging from

size 10×10 to 360×360. As demonstrated previously, the TRNG, energy computation, and acceptance modules exhibit negligible error in small-scale experiments. We do not expect the error in the TRNG and energy computation modules to increase in larger systems because these circuits are entirely digital. While the acceptance module receives analog inputs, the gain of the three-stage inverter is sufficiently large that at a given switching threshold, the output of this module should exhibit little-to-no error as the system size increases. The largest source of computation error in the annealing accelerator is the analog summation module. During the addition operation, the input terminal acts as the drain of the transistor. As the capacitor charges, the effective gate-to-source voltage (V_{GS}) progressively decreases. Conversely, during subtraction, the application of a negative voltage causes the input terminal to act as the source, resulting in a fixed V_{GS}. As a consequence, addition and subtraction do not occur symmetrically for fixed back-gate voltages and input voltage magnitudes.

The impact of summation error on the solution acceptance process largely depends on the ideal ΔH and the annealing temperature. In some cases, the ideal ΔH value is not close to the V_{SW} corresponding to the temperature prescribed by the annealing schedule. For example, if V_{SW} is 500 mV and the ideal ΔH is -250 mV, then significant summation error of 750 mV would be required to alter the acceptance decision. More generally, in cases where the ideal ΔH is negative, error is not likely to affect the decision process. However, if the ideal ΔH is positive, it is more likely that the ideal ΔH and V_{SW} are similar in magnitude. Consequently, this increases the possibility that a proposed solution is incorrectly accepted or rejected.

We utilize the SA accelerator simulation to further investigate the impact of experimental summation error on solution acceptance and SA convergence. We consider summation error in the SA accelerator simulation by adding a summation variability term to the ideal ΔH. In our previously discussed simulation results, the probability of acceptance ($P_{Acceptance}$) for a proposed solution was ~100% if $V_{SW} > \Delta H$ and ~0% if $V_{SW} \leq \Delta H$ following the thresholding function applied by the acceptance module. Considering finite summation error, $P_{Acceptance}$ is now a function of ΔH, the system temperature, and the summation variability term which is sampled from a normal distribution with a mean and standard deviation of μ_{Sum} and σ_{Sum}, respectively. **Extended Data Fig. 22** shows $P_{Acceptance}$ as a function ΔH for five different temperatures (T_0 to T_4) with $\mu_{Sum} = -0.041\,V$ and $\sigma_{Sum} = 0.24\,V$. Note that $P_{Acceptance}$ at each ΔH and system temperature was determined numerically by collecting 10,000 samples from the variability distribution. $P_{Acceptance}$ represents the proportion of solutions that satisfy $V_{SW} > \Delta H + N(\mu_{Sum}, \sigma_{Sum}^2)$. As expected, $P_{Acceptance}$ reduces with increasing ΔH and changes in the system temperature cause a shift in the $P_{Acceptance}$ curve.

The modified SA accelerator simulation was evaluated in a variety of system sizes (10 ×10, 20 × 20, 40 × 40, 80 × 80, 160 × 160, and 320 × 320) with different magnitudes of σ_{Sum} ranging from 0 V to 1 V. The convergence rate, i.e., the proportion of SA initializations that converge to an ideal solution, was determined for a set of 100 SA initializations at each system size and σ_{Sum}

combination. **Extended Data Fig. 23a** shows the convergence rate as a function of σ_{Sum}. Clearly, the convergence rate decreases rapidly when σ_{Sum} surpasses ~ 0.3 V. Interestingly, there is modest variation in convergence rate across system sizes at a given σ_{Sum}. In our hardware summation circuit, the experimental σ_{Sum} was 0.24 V, indicating that the experimental summation error should not significantly degrade the SA convergence rate. However, increased levels of summation error beyond ~0.3 V effectively degrade the convergence rate for all system sizes. The system coverage, defined as the max possible system coverage divided by the highest realized system coverage, is a metric that can be utilized to evaluate the solution quality for a given SA initialization. The mean system coverage of the 100 SA initializations is shown for each system size and σ_{Sum} in **Extended Data Fig. 23b**. Here, increasing σ_{Sum} has a modest effect on mean system coverage. Mean system coverage also exhibits a weak dependence on system scale, remaining above ~95% across all grid sizes. Although large summation error can prevent convergence to the global maximum coverage, the best solution generated by the SA accelerator simulation is still likely to achieve high coverage.

Despite the minimal impact of experimental summation error on convergence rate, error mitigation techniques can be employed that may increase SA convergence rate. Here, we discuss an algorithmic error mitigation strategy before considering a circuit-level mitigation strategy. First, the annealing schedule can be modified to increase the convergence rate. For an $(A \times A)$ system, we set the number of iterations per temperature to $A * I$, where I is a multiplicative factor. The 10×10 four-drone system with three obstacles that was evaluated in hardware was utilized to investigate the impact of I on convergence rate. The initial positions of all drones were kept constant across hardware and software initializations. The convergence rate is shown as a function of I in **Extended Data Fig. 23c**. The previously reported median convergence rate of 66% was obtained using $I = 3$, corresponding to 30 iterations per temperature. Clearly, the convergence rate increases monotonically with I, indicating that higher convergence rates can be achieved by increasing the number of iterations per temperature. However, this increase in convergence rate comes at the expense of additional computation time and resources. Also note that the set of temperatures prescribed by the annealing schedule is another critical SA parameter. We utilized five descending temperatures for this analysis. Different temperature sets would likely yield varying results. For very large systems, a digital counter could be utilized as a more robust and scalable summation circuit. However, a digital counter would require an increase in transistor count relative to the current summation circuit.

References

[1] H. Ravichandran *et al.*, "A stochastic encoder using point defects in two-dimensional materials," *Nature communications,* vol. 15, no. 1, p. 10562, 2024.

[2] T. Knobloch *et al.*, "Improving stability in two-dimensional transistors with amorphous gate oxides by Fermi-level tuning," *Nature Electronics,* vol. 5, no. 6, pp. 356–366, 2022.

[3] N. Fang, K. Nagashio, and A. Toriumi, "Experimental detection of active defects in few layers MoS2 through random telegraphic signals analysis observed in its FET characteristics," *2D Materials,* vol. 4, no. 1, p. 015035, 2017.

[4] M. Ghorbani-Asl, S. Kretschmer, D. E. Spearot, and A. V. Krasheninnikov, "Two-dimensional MoS2 under ion irradiation: from controlled defect production to electronic structure engineering," *2D Materials,* vol. 4, no. 2, p. 025078, 2017.

[5] A. Dodda, *Novel Materials and Devices for Low-Power Sensing and Security Applications*. The Pennsylvania State University, 2022.

[6] O. Canpolat, A. G. Yağlıkçı, G. F. Oliveira, A. Olgun, O. Ergin, and O. Mutlu, "Understanding the security benefits and overheads of emerging industry solutions to dram read disturbance," *arXiv preprint arXiv:2406.19094,* 2024.

[7] S. Song, P. Huang, W. Shen, L. Liu, and J. Kang, "A 3.3-Mbit/s true random number generator based on resistive random access memory," *Science China Information Sciences,* vol. 66, no. 11, p. 219402, 2023.

[8] Z. Wei *et al.*, "True random number generator using current difference based on a fractional stochastic model in 40-nm embedded ReRAM," in *2016 IEEE International Electron Devices Meeting (IEDM)*, 2016: IEEE, pp. 4.8. 1–4.8. 4.

[9] K. Schnieders *et al.*, "Exploiting read noise of filamentary VCM ReRAM for robust TRNG," *IEEE Transactions on Electron Devices,* 2025.

[10] S. Balatti, S. Ambrogio, and D. Ielmini, "Normally-off logic based on resistive switches—Part I: Logic gates," *IEEE transactions on Electron Devices,* vol. 62, no. 6, pp. 1831–1838, 2015.

[11] S. Balatti, S. Ambrogio, and D. Ielmini, "Normally-off logic based on resistive switches—Part II: Logic circuits," *IEEE Transactions on Electron Devices,* vol. 62, no. 6, pp. 1839–1847, 2015.

[12] N. Lepri, M. Baldo, P. Mannocci, A. Glukhov, V. Milo, and D. Ielmini, "Modeling and compensation of IR drop in crosspoint accelerators of neural networks," *IEEE Transactions on Electron Devices,* vol. 69, no. 3, pp. 1575–1581, 2022.

[13] H. Ravichandran *et al.*, "A peripheral-free true random number generator based on integrated circuits enabled by atomically thin two-dimensional materials," *ACS nano,* vol. 17, no. 17, pp. 16817–16826, 2023.

[14] S. Balatti *et al.*, "Physical unbiased generation of random numbers with coupled resistive switching devices," *IEEE Transactions on Electron Devices,* vol. 63, no. 5, pp. 2029–2035, 2016.

[15] R. Serrano *et al.*, "A fully digital true random number generator with entropy source based in frequency collapse," *IEEE Access,* vol. 9, pp. 105748–105755, 2021.

[16] R. Serrano *et al.*, "A robust and healthy against PVT variations TRNG based on frequency collapse," *IEEE Access,* vol. 10, pp. 41852–41862, 2022.

[17] Y. Lu *et al.*, "Jitter-quantizing-based TRNG robust against PVT variations," *IEEE Access,* vol. 8, pp. 108482–108490, 2020.

[18] S. Tao and E. Dubrova, "TVL-TRNG: Sub-microwatt true random number generator exploiting metastability in ternary valued latches," in *2017 IEEE 47th International Symposium on Multiple-Valued Logic (ISMVL)*, 2017: IEEE, pp. 130–135.
[19] R. Carboni and D. Ielmini, "Stochastic memory devices for security and computing," *Advanced Electronic Materials,* vol. 5, no. 9, p. 1900198, 2019.

Reviewer #2 (Remarks to the Author):

I have carefully reviewed the authors' rebuttal and the revised manuscript. The authors have addressed some of the previous concerns, particularly regarding error analysis and terminology clarification. However, several critical issues related to the hardware implementation and its characterization remain unresolved.

We thank the reviewer for carefully assessing our rebuttal and revised manuscript. We have prepared the following document to respond to their remaining comments. we look forward to any further communications that will allow us to strengthen the clarity and quality of the manuscript.

1) The authors have provided an image of the two chips in the probe station (Fig. R2). However, the manuscript still lacks detailed optical images of the key functional modules (Energy Computation, Summation, and Acceptance modules) as physically implemented on these chips. Currently, only the TRNG module and isolated logic gates (NAND, NOR and Inverter) are shown. More importantly, the authors confirm that "all measurements were performed modularly" with external components. This acknowledges that a fully integrated, single-chip, end-to-end hardware demonstration was not achieved. I would recommend that the revised manuscript should at least provide images of the actual Energy Computation and Acceptance circuit blocks as fabricated. Furthermore, to avoid any misleading, the manuscript should explicitly describe the modular hardware implementation and discuss the limitation of such modular demonstration.

We thank the reviewer for this recommendation. The energy computation module comprises one environment computation submodule that determines the surrounding environment from the perspective of the moving drone and two overlap computation submodules that calculate the overlap unique to the proposed solution (H_{New}) and the overlap unique to the prior solution (H_{Prior}). **Fig. R1a** shows an optical image of the environment computation submodule that consists of two NOR gates and one NAND gate. This submodule receives bitstreams corresponding to the occupancy of the 3 stationary drones ($S_1, S_2,$ and S_3) and the obstacles (O) as its inputs and generates the environment variable (Env) as its output. **Fig. R1b** and **Fig. R1c** show optical images of the overlap computation submodules that compute H_{New} and H_{Prior}, respectively. Both overlap computation submodules consist of one NAND gate and one NOR gate. The new-overlap submodule takes the bitstream corresponding to the proposed occupancy of the moving drone (M') and Env as inputs and computes H_{New} while the prior-overlap submodule takes the bitstream corresponding to the initial occupancy of the moving drone (M) and Env as inputs and computes H_{Prior}. We would like to note that an optical image of the acceptance module is already present in the previously submitted version of the manuscript (**Fig. 2n**). Furthermore, we agree that the modular hardware implementation and its inherent limitations should be explicitly discussed.

The 'Accelerator modules created with two-dimensional transistors' section of the revised manuscript was modified to include the following commentary and figures that present optical images of the energy computation module. (Pg. 12): After determination of the moving agent and its movement direction, the proposed agent location is updated and given to the energy computation module as a binary bitstream. This module is separated into three submodules; one environment computation submodule that determines the surrounding environment from the perspective of the moving drone and two overlap computation submodules that calculate the overlap unique to the prior solution and the overlap unique to the proposed solution. A transistor level circuit diagram depicting the entire energy computation module is shown in **Extended Data Fig. 8**. Optical images of the environment computation submodule and two overlap computation submodules are shown in **Extended Data Fig. 9a-c**, respectively, with relevant input and output nodes labeled. The environment computation submodule comprises one NAND gate and two NOR gates while the overlap computation modules comprise one NAND gate and one NOR gate. A representative NAND gate and its corresponding logic output are shown in **Fig. 2h** and **Fig. 2i**, respectively. Similarly, an optical image of a representative NOR gate and its corresponding logic output are shown in **Fig. 2j** and **Fig. 2k**, respectively. The Boolean logic functions implemented by these submodules will be discussed in the following section.

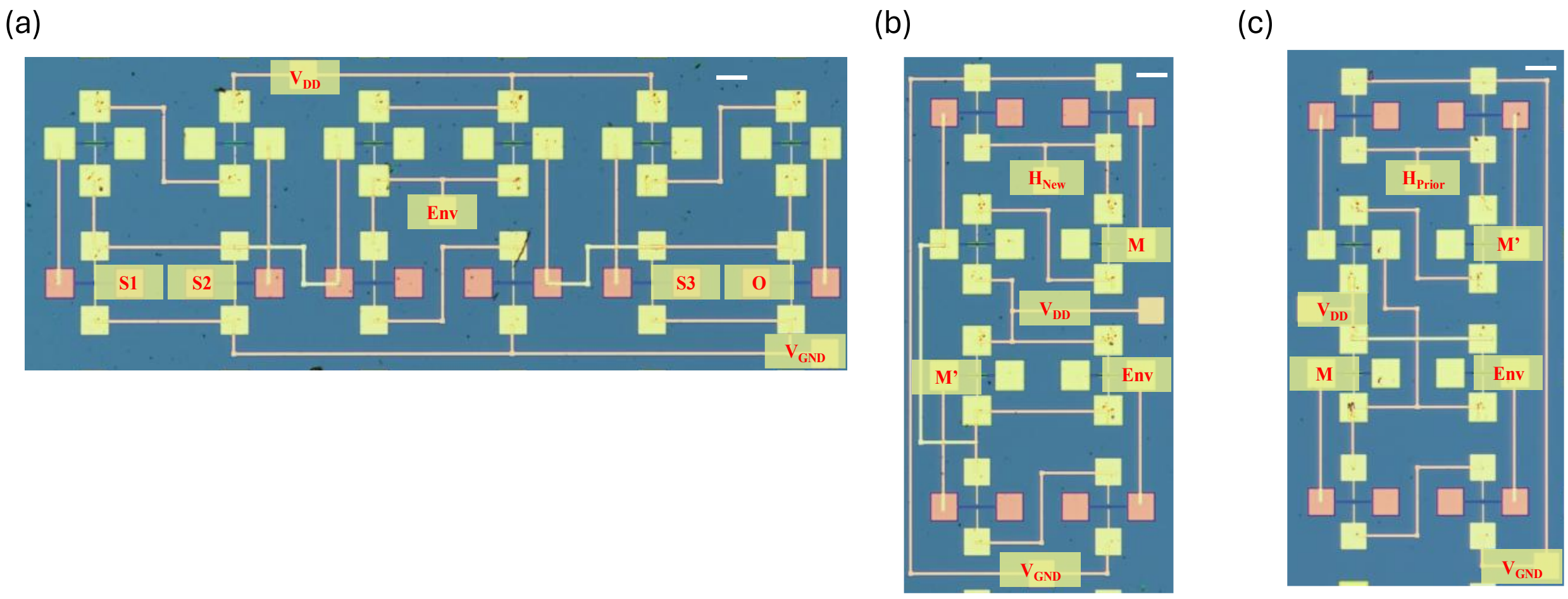

Fig. R1 Optical images of environment and overlap computation submodules. (a) *An optical image of the environment computation submodule that consists of two NOR gates and one NAND gate which receives* S_1, S_2, S_3, *and* O *as its inputs and generates* Env *as its output. (scale bar: 40 μm). Optical images of the* ***(b)*** *new and* ***(c)*** *prior overlap computation submodules both consisting of one NAND and one NOR gate. The new-overlap submodule takes* M' *and* Env *as inputs and computes* H_{New} *while the prior-overlap submodule takes* M *and* Env *as inputs and computes the prior overlap* H_{Prior} *(scale bar: 40 μm).*

The 'Drone placement in a constrained environment' section of the revised manuscript was modified to explicitly state that measurements were performed modularly and discuss the limitation of a modular demonstration. (Pg. 24): In our implementation of simulated annealing, all core operations including random number generation, energy computation, summation, and solution evaluation are performed on-chip by the TRNG, energy computation, summation, and

acceptance modules, respectively. For this hardware demonstration, each module was measured separately in a probe station. Additional details regarding the measurement setup are present in the **Methods** section. While this modular hardware implementation serves as proof-of-concept, further development is required to create a fully integrated hardware-based annealing accelerator.

2) The reported energy consumption of ~76 nJ per iteration is a key metric. However, it is still unclear if this final figure includes all relevant energy overheads, such as these from the external microcontroller (Extended Data Fig. 18).

The reviewer raises a valid concern. The hardware-based annealing accelerator modules demonstrated in this work perform random number generation, energy computation, summation, and solution evaluation. These modules perform operations that would normally be carried out by an arithmetic logic unit (ALU) within a central processing unit (CPU). In addition to computation, the CPU fetches instructions from memory, interprets them, and generates the control signals required to coordinate the operation of the input/output (I/O) modules. A larger microcontroller will also contain memory and general-purpose I/O ports that enable communication with the external environment. Given that our annealing accelerator modules specifically replace the computation that would occur in an ALU, it does not seem pertinent to list energy consumption that arises from external control signals, I/O modules, and memory units.

Beyond annealing computation, the energy overhead due to external controllers will vary significantly based on the type of control and memory units. Microcontrollers offer a high degree of flexibility, making them especially valuable in academic research, where rapid prototyping and proof-of-concept demonstrations are often more important than developing a fully optimized system. For instance, in the field of in-memory computing, printed circuit boards (PCBs) equipped with microcontrollers are commonly used to supply input signals to memory arrays and to automatically read back the resulting data.

To achieve a fully integrated annealing system, it would be necessary to design a complete application-specific integrated circuit (ASIC) incorporating a control unit, a non-volatile memory block for instruction storage, and a volatile memory block for storage of the bitstreams that represent the drone positions. The annealing accelerator modules would serve as the core computational engine of such an ASIC. Since an ASIC can be custom designed for a specific task, it would include only the necessary electronic components/digital gates, resulting in a significantly (orders of magnitude) lower energy consumption compared to a general-purpose processor or microcontroller performing the same function. Although such an ASIC would be significantly simpler than common microcontrollers, featuring fewer registers, a simplified ALU limited to basic Boolean operations, less complex communication protocols, and substantially reduced area and power consumption, its design and implementation lie far beyond the scope of the proof-of-concept targeted in this work.

Nonetheless, it is important to clearly define the system components that contribute to our final energy consumption metric. The abstract of the revised manuscript was modified to more clearly articulate that we specifically consider the energy consumption of the hardware modules. (Pg. 2): For a representative drone placement task, our hardware-based annealing

accelerator circuit modules consume 76 nJ of energy per iteration and achieve an 1800-fold search acceleration relative to exhaustive brute-force exploration of the solution space.

The 'Drone placement in a constrained environment' section of the revised manuscript also explicitly states that the energy consumption metric corresponds to the energy consumption of the hardware modules. (Pg. 21): To perform this optimization task, the hardware modules in the annealing accelerator consume approximately 76.44 nJ of energy per iteration depending on the nature of the prior and proposed solutions.

3) The authors note a discrepancy between the hardware convergence iteration (134) and the median software simulation convergence (113.5), attributing it to algorithmic stochasticity. To perform a more controlled and insightful comparison, I would recommend the authors run their software simulations using the exact sequence of random bits generated and used by the physical TRNG module during the hardware experiment.

We thank the reviewer for this recommendation. However, we believe that the revised version of the manuscript already incorporates an analysis that compares the experimental results with the output of the annealing accelerator simulation under identical conditions, i.e., with the string of experimental TRNG bits, the experimentally utilized annealing schedule, and matching initial drone positions. This segment of text discusses the error introduced by each hardware module during the hardware-based annealing experiment and concludes stating that the hardware-based annealing accelerator does not deviate from the ideal movement decisions determined by the annealing accelerator simulation: (Pg. 19): The annealing accelerator simulation was utilized to quantify the error introduced by each hardware module. Ideal values for the output of the energy computation module, the summation module, and the acceptance module were determined through simulation utilizing an identical initialization of the four-drone, three-obstacle 10x10 system and the experimentally realized string of TRNG bits. The experimental and ideal values of H_{Prior} and H_{New} are shown for all 150 simulated annealing iterations in **Extended Data Fig. R15a-b**, respectively, alongside the module errors. Note that 15,000 total time steps are shown corresponding to 100-time steps per iteration. Comparison of experimental and ideal values of H_{Prior} and H_{New} reveals that the 2D CMOS based logic circuits exhibit negligible error. Next, the experimental and ideal outputs of the summation module are shown alongside the module error for all 150 simulated annealing iterations in **Extended Data Fig. R15c**. During each iteration, this module sums 200 values corresponding to $-H_{Prior}$ and H_{New}. The mean absolute error (MAE) of this module is approximately 189.9 mV. Here, it is important to note that despite the MAE of 189.9 mV, the hardware-based annealing accelerator does not deviate from the ideal movement decisions determined by the annealing accelerator simulation. Subsequently, the experimental and ideal outputs of the acceptance module are shown alongside the module error for all 150 simulated annealing iterations in **Extended Data Fig. R15d**. The acceptance module error is negligible and will not impact subsequent logic operations.

Please let us know if we have misunderstood the comment or if this analysis is insufficient.

4) While the authors state they have replaced "hardware-realistic simulation" with "simulation of the annealing accelerator", I note that the term "hardware-realistic" still appears in the revised manuscript (e.g., Page 27 Figure title: " Hardware-realistic simulation of SA algorithm."). The authors should ensure consistent terminology throughout the manuscript, replacing all instances with the clarified description.

We thank the reviewer for highlighting this oversight. We have ensured that the revised version of the manuscript uses consistent terminology by removing all remaining instances of ‘Hardware-realistic.’
